# The ethylene-responsive transcription factor PpERF9 represses *PpRAP2.4* and *PpMYB114* via histone deacetylation to inhibit anthocyanin biosynthesis in pear

**DOI:** 10.1093/plcell/koad077

**Published:** 2023-03-14

**Authors:** Junbei Ni, Simai Wang, Wenjie Yu, Yifei Liao, Chen Pan, Manman Zhang, Ruiyan Tao, Jia Wei, Yuhao Gao, Dongsheng Wang, Songling Bai, Yuanwen Teng

**Affiliations:** Department of Horticulture, Zhejiang University, Hangzhou, Zhejiang 310058, People’s Republic of China; Zhejiang Provincial Key Laboratory of Integrative Biology of Horticultural Plants, Hangzhou 310058, People’s Republic of China; The Key Laboratory of Horticultural Plant Growth, Development and Quality Improvement, the Ministry of Agriculture of China, Hangzhou 310058, People’s Republic of China; Department of Horticulture, Zhejiang University, Hangzhou, Zhejiang 310058, People’s Republic of China; Zhejiang Provincial Key Laboratory of Integrative Biology of Horticultural Plants, Hangzhou 310058, People’s Republic of China; The Key Laboratory of Horticultural Plant Growth, Development and Quality Improvement, the Ministry of Agriculture of China, Hangzhou 310058, People’s Republic of China; Department of Horticulture, Zhejiang University, Hangzhou, Zhejiang 310058, People’s Republic of China; Zhejiang Provincial Key Laboratory of Integrative Biology of Horticultural Plants, Hangzhou 310058, People’s Republic of China; The Key Laboratory of Horticultural Plant Growth, Development and Quality Improvement, the Ministry of Agriculture of China, Hangzhou 310058, People’s Republic of China; Department of Horticulture, Zhejiang University, Hangzhou, Zhejiang 310058, People’s Republic of China; Zhejiang Provincial Key Laboratory of Integrative Biology of Horticultural Plants, Hangzhou 310058, People’s Republic of China; The Key Laboratory of Horticultural Plant Growth, Development and Quality Improvement, the Ministry of Agriculture of China, Hangzhou 310058, People’s Republic of China; Hainan Institute of Zhejiang University, Sanya 572000, People’s Republic of China; Department of Horticulture, Zhejiang University, Hangzhou, Zhejiang 310058, People’s Republic of China; Zhejiang Provincial Key Laboratory of Integrative Biology of Horticultural Plants, Hangzhou 310058, People’s Republic of China; The Key Laboratory of Horticultural Plant Growth, Development and Quality Improvement, the Ministry of Agriculture of China, Hangzhou 310058, People’s Republic of China; Hainan Institute of Zhejiang University, Sanya 572000, People’s Republic of China; Department of Horticulture, Zhejiang University, Hangzhou, Zhejiang 310058, People’s Republic of China; Zhejiang Provincial Key Laboratory of Integrative Biology of Horticultural Plants, Hangzhou 310058, People’s Republic of China; The Key Laboratory of Horticultural Plant Growth, Development and Quality Improvement, the Ministry of Agriculture of China, Hangzhou 310058, People’s Republic of China; Hainan Institute of Zhejiang University, Sanya 572000, People’s Republic of China; Department of Horticulture, Zhejiang University, Hangzhou, Zhejiang 310058, People’s Republic of China; Zhejiang Provincial Key Laboratory of Integrative Biology of Horticultural Plants, Hangzhou 310058, People’s Republic of China; The Key Laboratory of Horticultural Plant Growth, Development and Quality Improvement, the Ministry of Agriculture of China, Hangzhou 310058, People’s Republic of China; Department of Horticulture, Zhejiang University, Hangzhou, Zhejiang 310058, People’s Republic of China; Zhejiang Provincial Key Laboratory of Integrative Biology of Horticultural Plants, Hangzhou 310058, People’s Republic of China; The Key Laboratory of Horticultural Plant Growth, Development and Quality Improvement, the Ministry of Agriculture of China, Hangzhou 310058, People’s Republic of China; Department of Horticulture, Zhejiang University, Hangzhou, Zhejiang 310058, People’s Republic of China; Zhejiang Provincial Key Laboratory of Integrative Biology of Horticultural Plants, Hangzhou 310058, People’s Republic of China; The Key Laboratory of Horticultural Plant Growth, Development and Quality Improvement, the Ministry of Agriculture of China, Hangzhou 310058, People’s Republic of China; Institute of Horticulture, Henan Academy of Agricultural Sciences, Zhengzhou 450002, People’s Republic of China; Department of Horticulture, Zhejiang University, Hangzhou, Zhejiang 310058, People’s Republic of China; Zhejiang Provincial Key Laboratory of Integrative Biology of Horticultural Plants, Hangzhou 310058, People’s Republic of China; The Key Laboratory of Horticultural Plant Growth, Development and Quality Improvement, the Ministry of Agriculture of China, Hangzhou 310058, People’s Republic of China; Department of Horticulture, Zhejiang University, Hangzhou, Zhejiang 310058, People’s Republic of China; Zhejiang Provincial Key Laboratory of Integrative Biology of Horticultural Plants, Hangzhou 310058, People’s Republic of China; The Key Laboratory of Horticultural Plant Growth, Development and Quality Improvement, the Ministry of Agriculture of China, Hangzhou 310058, People’s Republic of China; Hainan Institute of Zhejiang University, Sanya 572000, People’s Republic of China

## Abstract

Ethylene induces anthocyanin biosynthesis in most fruits, including apple (*Malus domestica*) and plum (*Prunus* spp.). By contrast, ethylene inhibits anthocyanin biosynthesis in pear (*Pyrus* spp.), but the underlying molecular mechanism remains unclear. In this study, we identified and characterized an ethylene-induced ETHYLENE RESPONSE FACTOR (ERF) transcription factor, PpETHYLENE RESPONSE FACTOR9 (PpERF9), which functions as a transcriptional repressor. Our analyses indicated PpERF9 can directly inhibit expression of the MYB transcription factor gene *PpMYB114* by binding to its promoter. Additionally, PpERF9 inhibits the expression of the transcription factor gene *PpRELATED TO APETALA2.4* (*PpRAP2.4*), which activates *PpMYB114* expression, by binding to its promoter, thus forming a PpERF9-PpRAP2.4-PpMYB114 regulatory circuit. Furthermore, PpERF9 interacts with the co-repressor PpTOPLESS1 (PpTPL1) via EAR motifs to form a complex that removes the acetyl group on histone H3 and maintains low levels of acetylated H3 in the *PpMYB114* and *PpRAP2.4* promoter regions. The resulting suppressed expression of these 2 genes leads to decreased anthocyanin biosynthesis in pear. Collectively, these results indicate that ethylene inhibits anthocyanin biosynthesis by a mechanism that involves PpERF9-PpTPL1 complex-mediated histone deacetylation of *PpMYB114* and *PpRAP2.4*. The data presented herein will be useful for clarifying the relationship between chromatin status and hormone signaling, with implications for plant biology research.

## Teaser

The ethylene-activated PpERF9-TOPLESS complex suppresses pear anthocyanin biosynthesis by inhibiting *PpMYB114* and *PpRAP2.4* expression via histone deacetylation.

IN A NUTSHELL

**Background:** The red coloration of many fruits is the result of anthocyanin accumulation. The biosynthesis of anthocyanins is influenced by internal and environmental factors, including phytohormones. Ethylene induces anthocyanin biosynthesis in most fruits, including apples, grapes, and plums. However, ethylene inhibits anthocyanin biosynthesis in pears with different genetic backgrounds. Preliminary studies suggest that the transcription factors PpETHYLENE RESPONSE FACTOR9 and PpRELATED TO APETALA2.4 (PpRAP2.4) act upstream of *PpMYB114*, which encodes a key transcription factor in anthocyanin biosynthesis in pears. PpERF9 and PpRAP2.4 play important roles in ethylene-mediated inhibition of anthocyanin biosynthesis, and PpERF9 function is associated with histone modifications.

**Question:** What is the relationship between ethylene and the PpERF9 and PpRAP2.4 transcription factors? How do PpERF9 and PpRAP2.4 regulate anthocyanin biosynthesis in pear fruit? How does PpERF9 regulate anthocyanin biosynthesis in pear fruit through histone modification?

**Findings:** Our 4 major findings are the following: (i) ethylene induces the expression of *PpERF9* while inhibiting the expression of *PpRAP2.4* in pear fruit. (ii) PpERF9 directly inhibits *PpMYB114* expression by binding to its promoter, thereby repressing anthocyanin biosynthesis in pear fruit. (iii) PpRAP2.4 activates *PpMYB114* expression by binding to its promoter, thereby inducing anthocyanin biosynthesis in pear fruit, while PpERF9 directly represses the expression of *PpRAP2.4*, thus forming a PpERF9-PpRAP2.4-PpMYB114 regulatory circuit. (iv) PpERF9 interacts with the co-repressor PpTOPLESS1 via EAR motifs to form a complex that removes the acetyl group on histone H3, maintaining low levels of acetylated histone H3 in the promoter regions of *PpMYB114* and *PpRAP2.4.* Acetylated H3 is associated with the suppressed expression of these 2 genes and decreased anthocyanin biosynthesis in pear fruit.

**Next step:** It will be interesting to explore how ethylene inhibits the expression of *PpMYB10*, which encodes another important transcription factor regulating anthocyanin biosynthesis in pear fruit.

## Introduction

Anthocyanins are important specialized metabolites responsible for the development of different-colored (e.g. red, purple, and blue) flowers, fruits, leaves, seeds, and other plant tissues (Tanaka et al. 2008). Anthocyanins play various roles in plants, including aiding pollination and seed dispersal as well as protecting against environmental stresses (Tanaka et al. 2008; He and Giusti 2010). The anthocyanin biosynthesis pathway is conserved among seed plants. Anthocyanins are synthesized by a series of enzymes, including chalcone synthase (CHS), chalcone isomerase (CHI), flavanone 3′-hydroxylase (F3′H), dihydroflavonol 4-reductase (DFR), anthocyanin synthase (ANS), and UDP-glucose:flavonoid 3-glucosyltransferase (UFGT) (Winkel-Shirley 2001). The genes encoding these enzymes are anthocyanin biosynthetic structural genes.

Anthocyanin biosynthetic structural genes are transcriptionally regulated by a conserved MYB-bHLH-WDR (MBW) complex. The MBW complex comprises an R2R3-MYB transcription factor (TF), a bHLH TF, and a WD-repeat protein (Quattrocchio et al. 1999; Winkel-Shirley 2001; Gonzalez et al. 2008). The R2R3-MYB TFs are the core regulators of the MBW components and have been well studied in many plant species, including petunia (*Petunia hybrida*) (Quattrocchio et al. 1999), Arabidopsis (*Arabidopsis thaliana*) (Gonzalez et al. 2008; Dubos et al. 2010), and apple (Takos et al. 2006; Espley et al. 2007; Espley et al. 2009). In pear (*Pyrus* spp.), PpMYB10 and PpMYB114 are 2 major R2R3-MYB TFs that promote anthocyanin biosynthesis (Feng et al. 2010; Yao et al. 2017). In contrast to the highly conserved core activation complex (MBW), many anthocyanin repressors have been identified, including an R3-MYB (AtMYBL2) (Matsui et al. 2008), the subgroup 4 R2R3-MYB repressors (Dubos et al. 2010), the *miR156-SPL* module (Gou et al. 2011; Qian et al. 2017), and some HD-ZIP proteins (e.g. MdHB1) (Jiang et al. 2017).

Anthocyanin biosynthesis is regulated by various environmental factors (e.g. light, temperature, and nutrients) and plant hormones (Jaakola 2013). Most plant hormones, including jasmonate (JA), ethylene, gibberellins, and abscisic acid (ABA), are involved in the regulation of anthocyanin biosynthesis (Shan et al. 2009; Qi et al. 2011; Das et al. 2012; An et al. 2018a; An et al. 2018b). In many fruits, such as apple (*M. domestica*), grape (*Vitis vinifera*), plum (*Prunus* spp.), and strawberry (*Fragaria* × *ananassa*), ethylene usually induces anthocyanin biosynthesis (Faragher and Brohier 1984; El-Kereamy et al. 2003; Villarreal et al. 2010; Cheng et al. 2016). In apple, the MdEIN3-LIKE1 (MdEIL1) TF activates *MdMYB1* expression by binding to its promoter. Additionally, MdMYB1 induces ethylene production by activating the expression of *MdERF3*, which encodes a positive regulator of ethylene biosynthesis (An et al. 2018b). Thus, the EIL1-MYB1-ERF3 regulatory loop synergistically modulates ethylene and anthocyanin biosynthesis. In contrast, ethylene inhibits anthocyanin biosynthesis in pear (Ni et al 2020) and Arabidopsis (Jeong et al. 2010), but the molecular mechanism underlying this phenomenon in Arabidopsis remains unknown.

In pear, ethylene induces the expression of *PpERF105*, leading to the upregulated expression of *PpMYB140*, which encodes a repressive R2R3-MYB TF. A recent study revealed that PpMYB140 inhibits the expression of anthocyanin biosynthetic structural genes and competes with PpMYB114 to interact with PpbHLH3, thereby inhibiting anthocyanin biosynthesis and the development of red pear fruit (Ni et al. 2021). These findings partly clarify the molecular mechanism underlying the ethylene-inhibited anthocyanin biosynthesis in pear fruit, but cannot explain the observation that *PpMYB10* and *PpMYB114* are transcriptionally repressed by ethylene (Ni et al. 2021). This suggests there are additional repressive regulatory processes involved in the ethylene-inhibited anthocyanin accumulation in pear.

Transcriptional repression mechanisms are important in plants. Many TFs containing a typical repressive domain, the ERF-associated amphiphilic repression (EAR) motif, can inhibit the transcription of the downstream target genes (Fujimoto et al. 2000; Ohta et al. 2001). The EAR motif, identified as a conserved LxLxL or DLNxxP sequence in the C-terminal region, is the predominant transcriptional repression motif identified in plants (Kagale and Rozwadowski 2011). The EAR motif has been detected in various TF families, including ERF, AUX/IAA, and MYB (Ohta et al. 2001; Kagale and Rozwadowski 2011). The EAR motif-containing TFs interact with SWI-independent 3 (SIN3), SIN3-associated polypeptide of 18 kDa (SAP18), and TOPLESS (TPL), which are believed to establish a physical link between histone deacetylases (HDACs) and DNA-bound active repressors (Pazin and Kadonaga 1997; Hiratsu et al. 2002; Song et al. 2005; Song and Galbraith 2006; Kagale and Rozwadowski 2011), resulting in histone deacetylation, which is important for active transcriptional repression.

Specifically, EAR motif-containing TFs are involved in regulating anthocyanin biosynthesis, and considerable evidence indicates that anthocyanin biosynthetic structural genes are targeted for histone deacetylation. In Arabidopsis, MYB75 interacts with an EAR motif-containing HAT1 (i.e. HD-ZIP TF) and disrupts the formation of the MBW complex by recruiting the TPL/TPR co-repressors to form a complex that represses anthocyanin biosynthesis via histone H3 deacetylation at the transcription start sites of *DFR*, *LEUCOANTHOCYANIDIN DIOXYGENASE* (*LDOX*), and *UFGT* genes (Zheng et al. 2019). Moreover, a JA-responsive protein, EAR motif-containing adaptor protein (ECAP), links JAZ6/8 with a co-repressor, TPR2, to form a JAZ-ECAP-TPR2 (JET) complex that represses the expression of *ANS* via histone H3K9 deacetylation, which inhibits anthocyanin biosynthesis in Arabidopsis (Li et al. 2020). Thus, EAR motif-related histone deacetylation is crucial for inhibiting anthocyanin biosynthesis through the transcriptional regulation of anthocyanin biosynthetic structural genes in plants.

However, it remains unclear whether anthocyanin biosynthetic regulatory genes are negatively regulated by histone deacetylation. In this study, we focused on the repressive effect of ethylene on *PpMYB114* expression and constructed a PpERF9/PpTPL1-(PpRAP2.4)-PpMYB114 transcriptional regulatory cascade involving histone deacetylation. Our results reveal a regulatory pattern of ethylene-inhibited anthocyanin biosynthesis in pear, which provides insight into the molecular basis of hormone-regulated anthocyanin biosynthesis in plants.

## Results

### Identification of the ethylene-inducible transcriptional repressor PpERF9 and its inhibitory effect on *PpMYB114* transcript levels

We recently showed that ethylene inhibits anthocyanin biosynthesis in red pear fruit with different genetic backgrounds (Ni et al. 2020); this finding was further confirmed in this study by treating “Hongzaosu” pear fruit with ethephon or 1-methylcyclopropene (1-MCP, an ethylene inhibitor). The results showed that ethephon treatment inhibited “Hongzaosu” red coloration while 1-MCP induced coloration (Supplemental Fig. S1).

The expression levels of anthocyanin biosynthetic structural genes (*PpCHI*, *PpCHS*, *PpF3H*, *PpDFR*, *PpANS*, and *PpUFGT*) were significantly upregulated 3 d after a 1-MCP treatment, while the expression levels of *PpANS* and *PpUFGT* were repressed 3 d after ethephon treatment compared with control (Supplemental Fig. S1C). Furthermore, ethephon treatment slightly induced and then repressed the expression levels of *PpCHI*, *PpCHS*, and *PpF3H* compared with control. Notably, expression levels of *PpMYB*10** and *PpMYB114* increased more than 400-fold and 30-fold after the 1-MCP treatment (relative to the control level), respectively, whereas their expression levels were repressed significantly following the ethephon treatment compared with control (Supplemental Fig. S1D). These results indicate that ethylene inhibits red pear anthocyanin biosynthesis by reducing *PpMYB114* and *PpMYB10* mRNA levels. On the basis of these observations, we speculated that ethylene might inhibit *PpMYB114* and *PpMYB10* expression through the following 2 pathways. First, ethylene signaling might activate some repressors that directly inhibit *PpMYB114* and *PpMYB10* expression. Second, ethylene signaling might repress some activators that induce *PpMYB114* and *PpMYB10* expression.

We subsequently screened for potential *cis*-elements of *PpMYB114* and *PpMYB10* in anthocyanin regulatory gene promoters and detected a GCC-box and a dehydration-responsive element (DRE) motif (i.e. the binding sites of ERF TFs) in the *PpMYB114* and *PpMYB10* promoter, respectively, indicating that ethylene might regulate *PpMYB114* and *PpMYB10* expression through ERF TFs. From 21 previously identified ERF TFs (Ni et al. 2020), which were positively or negatively correlated with anthocyanin content as well as *PpMYB10* and *PpMYB114* expression, we further screened 2 ERF TFs (Pbr000396.1 and Pbr000398.1) that positively responded to ethylene signaling and were clustered into Class II with EAR motif (Supplemental Figs. S2 and S3).

**Figure 1.**
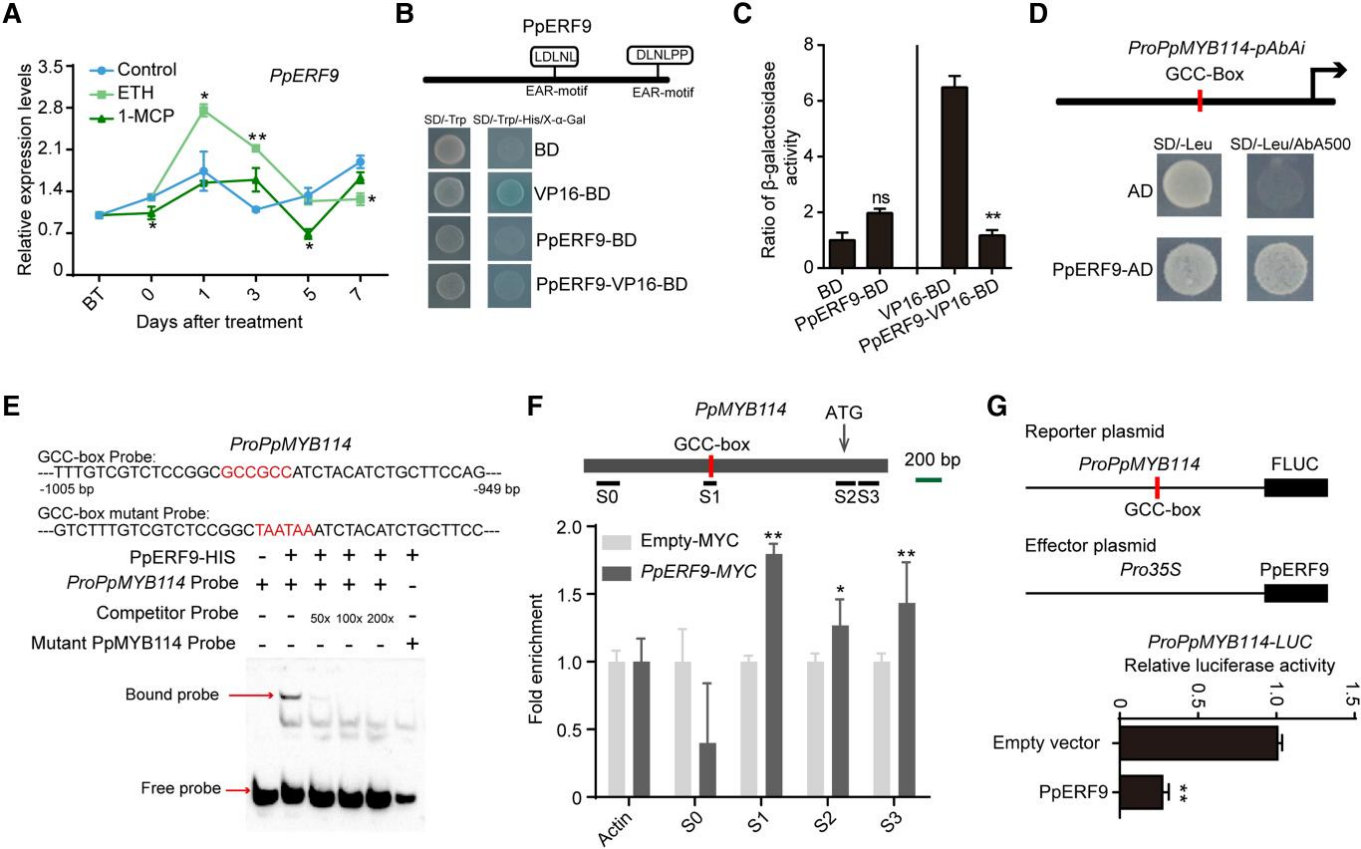
Ethylene-induced PpERF9 represses *PpMYB114* transcription. **A)** Expression pattern of *PpERF9* in “Hongzaosu” pear fruit following ethephon and 1-MCP treatments. **B)** Transcriptional repression associated with PpERF9 in yeast cells. **C)** The β-galactosidase activities reflect the transcriptional repression by PpERF9. **D)** A Y1H assay indicated that PpERF9 can bind directly to the *PpMYB114* promoter containing a GCC-box. **E)** An EMSA confirmed that PpERF9 binds directly to the GCC-box in the *PpMYB114* promoter. The hot probe was a biotin-labeled fragment of the *PpMYB114* promoter containing the GCC-box, whereas the competitor probe was an unlabeled probe (50-, 100-, and 200-fold molar excess). The mutant cold probe was the same as the labeled hot probe but with GCCGCC mutated to TAATAA. The His-tagged PpERF9 protein was purified. **F)** ChIP-qPCR assay results reflected the in vivo binding of PpERF9 to the *PpMYB114* promoter. Cross-linked chromatin samples were extracted from *PpERF9-MYC*-overexpressing pear calli with 3 biological replicates and precipitated with an anti-MYC antibody. Eluted DNA was used to amplify the sequences neighboring the GCC-box by qPCR. Four regions (S0 to S3) were examined. The bar indicates the length of 200 bp. Pear calli overexpressing the MYC sequence were used as a negative control. Three biological replicates of pear calli were used in the ChIP assay. **G)** A dual-luciferase assay demonstrated that PpERF9 directly inhibits *PpMYB114* promoter activity. The promoter of PpMYB114 was cloned into the pGreenII 0800-LUC (firefly luciferase) vector, and the full-length CDS of *PpERF9* was cloned into the pGreenII 0029 62-SK vector. The empty vector of SK was used as control. The relative luciferase activity was analyzed. Error bars represent the standard deviation of 3 biological replicates. Asterisks indicate significantly different values (*P < 0.05 and **P < 0.01).

When we cloned the coding sequences of these 2 ERF genes, we found that Pbr000396.1 had a 41-bp deletion compared with the genome sequence (Supplemental Fig. S4), which led to a frameshift mutation as well as the lack of the AP2 superfamily domain and EAR motif (Supplemental Fig. S5A). In addition, there are 2 EAR motifs in Pbr000398.1 (i.e. PpERF9), one of which (DLNxxP-type) is located in the C-terminal region, whereas the other (LxLxL-type) is located in the middle part of the protein (Fig. 1B and Supplemental Fig. S5B), suggesting that PpERF9 is a transcriptional repressor.

We analyzed *PpERF9* expression in “Hongzaosu” pear fruit treated with ethephon and 1-MCP. The *PpERF9* expression level peaked at 1 d after the ethephon treatment and then decreased, whereas it decreased slightly in response to the 1-MCP treatment (Fig. 1A). Thus, *PpERF9* expression is positively regulated by ethylene signaling. A transcriptional activity assay confirmed that PpERF9 is a repressive TF (Fig. 1, B and C). An examination of subcellular localization indicated that PpERF9 is present in the nucleus (Supplemental Fig. S6). Accordingly, ethylene-induced PpERF9 is a nuclear localized transcriptional repressor.

We then investigated the transcriptional repression of *PpMYB114* and *PpMYB10* by PpERF9. The results of a yeast one-hybrid (Y1H) assay and an electrophoretic mobility shift assay (EMSA) showed that PpERF9 binds to the GCC-box in the *PpMYB114* promoter in vitro (Fig. 1, D and E). A chromatin immunoprecipitation (ChIP)-qPCR assay indicated that immunoprecipitation of PpERF9 substantially enriched the S1 region of the *PpMYB114* promoter containing the GCC-box, reflecting the binding of PpERF9 to the *PpMYB114* promoter in vivo (Fig. 1F). A dual-luciferase assay confirmed that PpERF9 represses the activity of the *PpMYB114* promoter (Fig. 1G). However, a dual-luciferase assay indicated that PpERF9 did not transcriptionally regulate *PpMYB10* (Supplemental Fig. S7). These results suggest that PpERF9 inhibits *PpMYB114* transcription by binding to the GCC-box of the promoter.

### PpERF9 negatively regulates anthocyanin biosynthesis in pear

**Figure 2.**
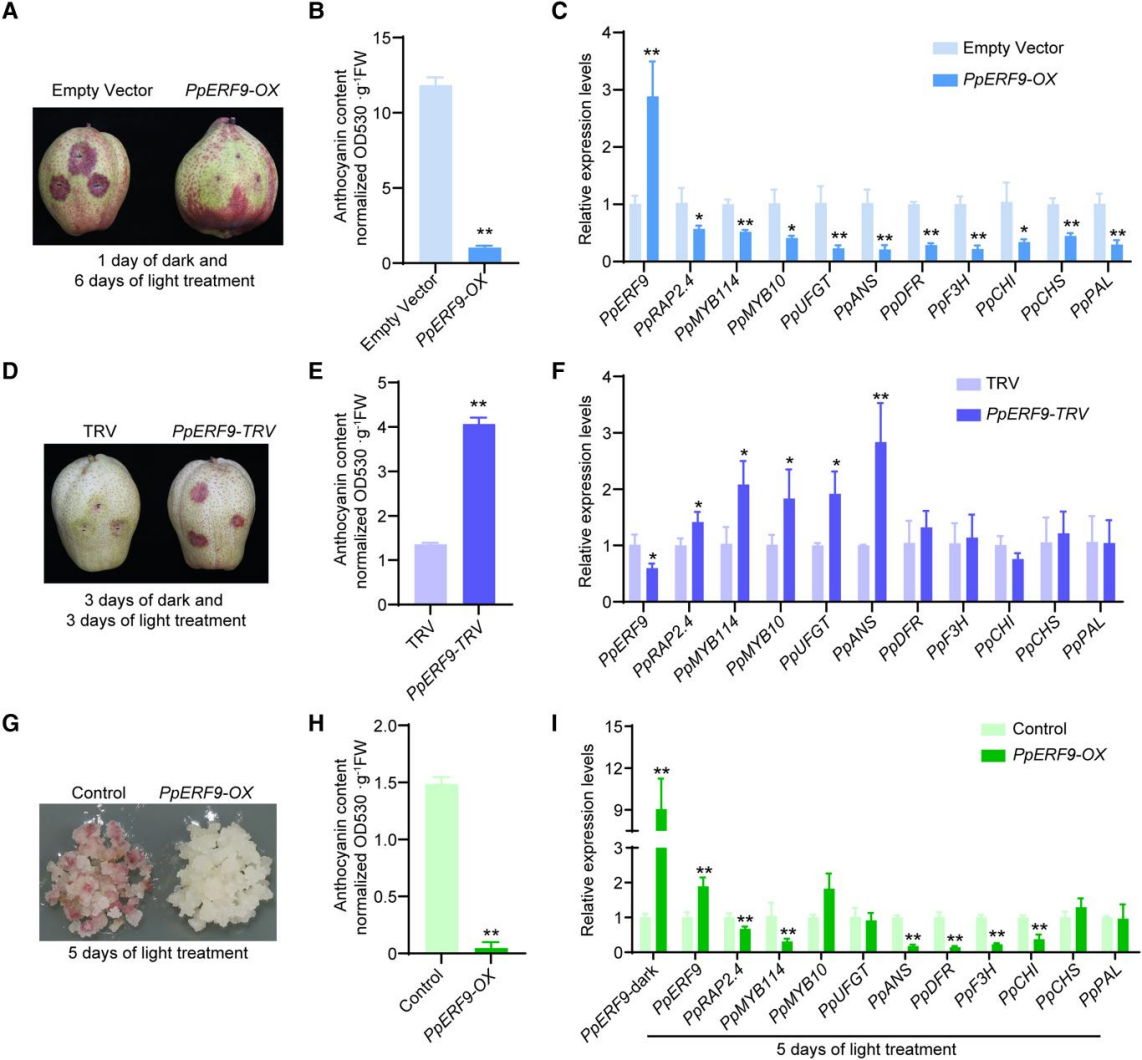
Functional analysis of PpERF9 in “Hongzaosu” pear fruit and “Clapp's Favorite” pear calli. **A)** Transient overexpression of *PpERF9* in fruit. The full-length CDS of *PpERF9* was inserted into the pCAMBIA1301 vector for the subsequent expression under the control of the 35S promoter. Pear fruits were infiltrated with *A. tumefaciens* EHA105 cells containing the recombinant plasmid using a needle-free syringe. Fruits infiltrated with an empty pCAMBIA1301 vector were used as a control. The phenotypes were examined after a 1-d dark and 6-d light treatment. **B)** Total anthocyanin content in fruit transiently overexpressing *PpERF9*. **C)** Expression patterns of genes related to anthocyanin biosynthesis in fruit transiently overexpressing *PpERF9*. **D)** Transient silencing of *PpERF9* in fruit. The empty vector (pTRV1 + pTRV2) was used as the negative control. Pear fruits were placed in darkness for 3 d and then treated with strong light for 3 d. **E)** Total anthocyanin content in fruit in which *PpERF9* was transiently silenced. **F)** Expression patterns of genes related to anthocyanin biosynthesis in fruit in which *PpERF9* was transiently silenced. **G)** Overexpression of *PpERF9* inhibited anthocyanin accumulation in pear calli. The calli transformed with the empty vector (pCAMBIA1301) were used as the negative control. Pear calli were incubated under strong light at 17°C for 5 d. **H)** Anthocyanin contents in *PpERF9*-OX pear calli after the light treatment. **I)** Expression levels of the anthocyanin-related genes, *PpRAP2.4* and *PpERF9* in *PpERF9*-OX pear calli after the light treatment. Error bars represent the standard deviation of 3 biological replicates. Asterisks indicate significantly different values (*P < 0.05 and **P < 0.01).

To clarify whether PpERF9 represses anthocyanin biosynthesis, we used bagged mature “Hongzaosu” pear fruit (with no anthocyanin biosynthesis) for transient overexpression analysis and virus-induced gene silencing (VIGS) assays. After a 6-d exposure to strong light, the peel of fruit overexpressing *PpERF9* (*PpERF9*-OX) was red, with the exception of the injection site (Fig. 2A). The peel anthocyanin content was much lower in the *PpERF9*-OX fruit than in the control fruit (Fig. 2B). The *PpRAP2.4*, *PpMYB114*, *PpMYB10*, *PpUFGT*, *PpANS*, *PpDFR*, *PpF3H*, *PpCHI*, *PpCHS*, and *PpPAL* expression levels were significantly lower in the *PpERF9-OX* pear fruit peel than in the control fruit peel (Fig. 2C). In contrast, the silencing of *PpERF9* promoted anthocyanin accumulation and the red coloration of pear fruit peel around the injection site (Fig. 2, D and E). The silencing of *PpERF9* significantly induced the expression of *PpMYB114*, *PpMYB10*, *PpUFGT*, and *PpANS* (Fig. 2F). These results indicate that PpERF9 inhibits the anthocyanin accumulation and coloration of pear fruit.

To further verify the effect of PpERF9 on anthocyanin biosynthesis, *PpERF9*-OX transgenic pear calli were generated. The control (empty vector) and *PpERF9*-OX pear calli were then treated with strong white light with UV-B (Fig. 2G), which induces anthocyanin biosynthesis in pear. The control pear calli turned red and accumulated large amounts of anthocyanin after a 5-d light treatment, whereas the *PpERF9*-OX pear calli remained pale, with relatively little accumulated anthocyanins (Fig. 2, G and H). The overexpression of *PpERF9* downregulated the expression of *PpMYB114*, *PpANS*, *PpDFR*, *PpF3H*, and *PpCHI* (Fig. 2I). Considered together, these results imply that PpERF9 negatively affects anthocyanin biosynthesis and the red coloration of pear fruit by inhibiting *PpMYB114* expression.

### Identification of the ethylene-repressive TF PpRAP2.4 and its effects on *PpMYB114* expression

Because *PpERF9* expression did not decrease significantly in the first 3 d after 1-MCP treatment (Fig. 1A), the rapid upregulation of *PpMYB114* expression and anthocyanin accumulation in pear after the 1-MCP treatment could not be explained. Thus, we speculated that there might be another regulatory pathway involved in ethylene-inhibited anthocyanin biosynthesis. A co-expression network analysis using our previous transcriptome data (Ni et al. 2020) identified 4 AP2/ERF TF genes, Pbr009056.1, Pbr016224.1, Pbr002023.1, and Pbr016049.2, which had expression patterns that were positively related to *PpMYB114* and *PpMYB10* expression (Supplemental Fig. S2).

A phylogenetic analysis indicated that Pbr009056.1 and Pbr016224.1 were Class II repressors, while Pbr002023.1 and Pbr016049.2 were homologous genes of AT2G41710.1 (AtSMOS1) and AT1G22190.1 (AtRAP2.4), respectively (Supplemental Fig. S3). AtSMOS1 and AtRAP2.4 were reported to be transcriptional activators in previous studies (Li et al. 2022; Nomoto et al. 2022). Thus, Pbr002023.1 and Pbr016049.2 might also be transcriptional activators. However, Pbr002023.1 was expressed at very low levels in all samples (Supplemental Fig. S2). Thus, Pbr016049.2, which was named *PpRAP2.4* according to the name of the homologous gene in Arabidopsis, was selected for further analyses. Furthermore, Pbr016049.2 showed no close relationship with the reported ERF TFs which regulated anthocyanin biosynthesis in apple and strawberry in the phylogenetic tree, indicating that PpRAP2.4 might be a nonconserved regulator of anthocyanin biosynthesis.

**Figure 3.**
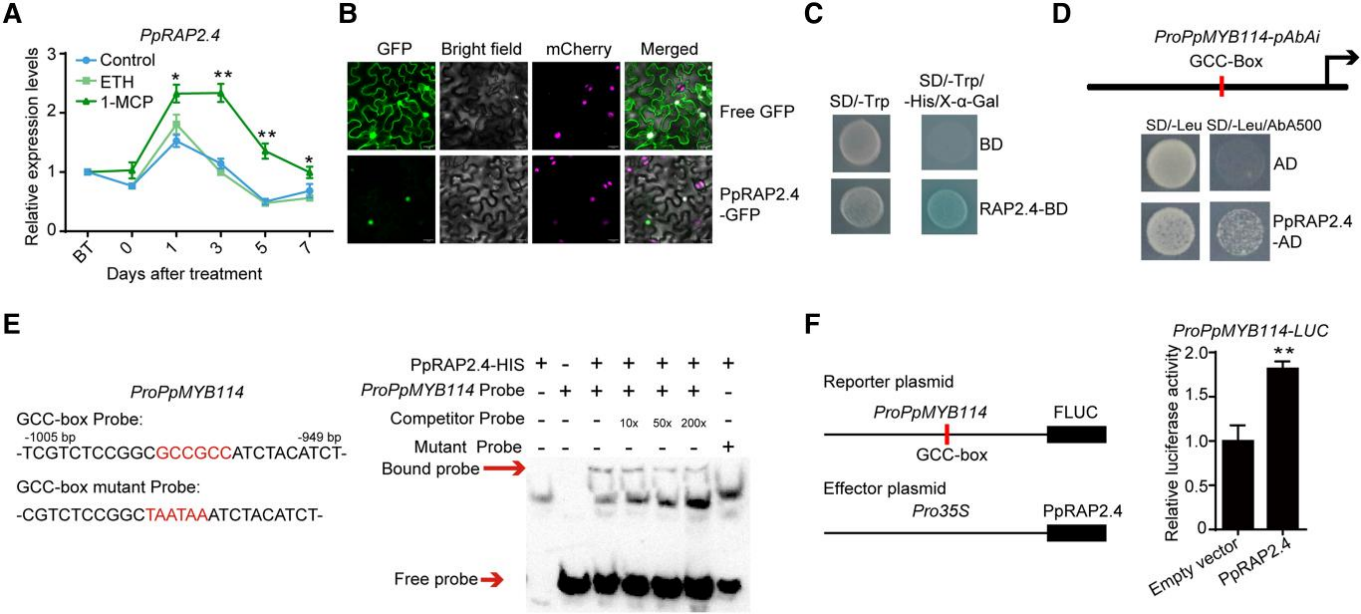
Ethylene-repressed PpRAP2.4 induces *PpMYB114* transcription. **A)** Expression pattern of *PpRAP2.4* in “Hongzaosu” pear fruit following ethephon and 1-MCP treatments. **B)** Subcellular localization of PpRAP2.4 in *N. benthamiana* leaf cells. **C)** Transcriptional activation associated with PpRAP2.4 in yeast cells. **D)** A Y1H assay indicated that PpRAP2.4 can bind to the *PpMYB114* promoter containing a GCC-box in vitro. **E)** An EMSA confirmed PpRAP2.4 binds to the GCC-box in the *PpMYB114* promoter in vitro. The probe was a biotin-labeled fragment of the *PpMYB114* promoter containing the GCC-box, whereas the competitor probe was an unlabeled probe (10-, 50-, and 200-fold molar excess). The mutant cold probe was the same as the labeled hot probe but with GCCGCC mutated to TAATAA. The His-tagged PpRAP2.4 protein was purified and 3 µg PpRAP2.4-His protein was used for EMSA. **F)** A dual-luciferase assay indicated that PpRAP2.4 activates the *PpMYB114* promoter in vivo. The promoter of *PpMYB114* was cloned into the pGreenII 0800-LUC (firefly luciferase) vector, and the full-length CDS of *PpRAP2.4* was cloned into the pGreenII 0029 62-SK vector. The empty vector of pGreenII 0029 62-SK was used as control. The relative luciferase activity was analyzed. Error bars represent the standard deviation of 3 biological replicates. Asterisks indicate significantly different values (*P < 0.05 and **P < 0.01).

The expression of *PpRAP2.4* was upregulated by the 1-MCP treatment, but not by exposure to ethephon (Fig. 3A). Additionally, PpRAP2.4 was localized in the nucleus (Fig. 3B). A transcriptional activity assay confirmed that PpRAP2.4 is a transcriptional activator (Fig. 3C). We investigated the effects of PpRAP2.4 on *PpMYB114* and *PpMYB10* promoter activity. The Y1H assay and EMSA proved that PpRAP2.4 binds to the GCC-box of the *PpMYB114* promoter (Fig. 3, D and E). A dual-luciferase assay involving *Nicotiana benthamiana* leaves provided evidence that PpRAP2.4 induces *PpMYB114* promoter activity (Fig. 3F). However, dual-luciferase assay showed that PpRAP2.4 had no effect on the *PpMYB10* promoter activity (Supplemental Fig. S7B). These results indicated that PpRAP2.4 induces *PpMYB114* transcription by binding to the GCC-box of the promoter.

### PpRAP2.4 positively regulates anthocyanin biosynthesis in pear

**Figure 4.**
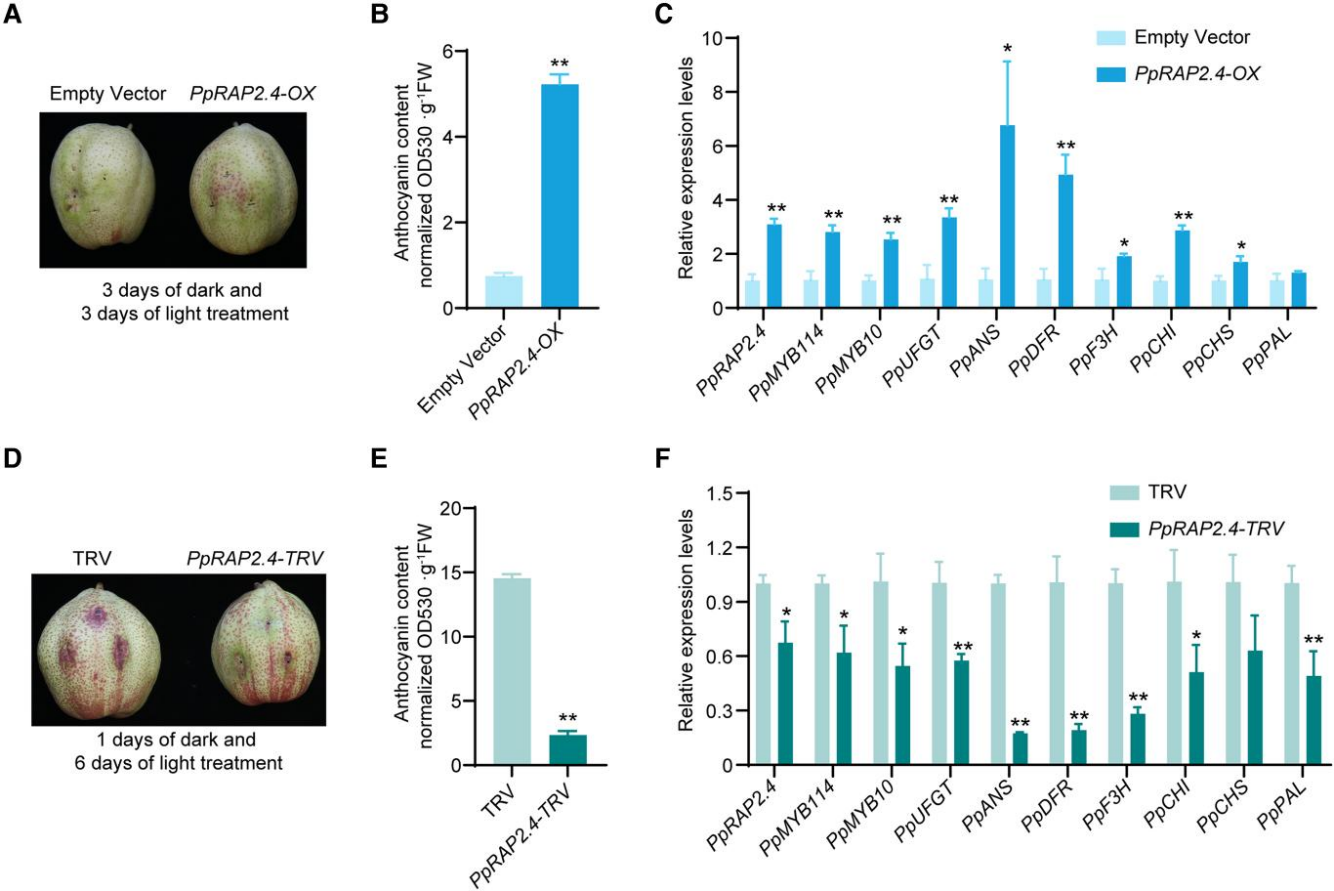
PpRAP2.4 positively regulates anthocyanin biosynthesis in “Hongzaosu” pear fruit. **A)** Transient overexpression of *PpRAP2.4* in fruit. *PpARAP2.4* was cloned into pCAMBIA1301 vector with a 35S promoter, and the empty vector (pCAMBIA1301) was used as the negative control. The plasmids were transformed into *A. tumefaciens* EHA105. After injection, the pear fruits were placed in dark for 3 d and then treated with strong light for 3 d. Then the phenotypes were examined. **B)** Total anthocyanin content in fruit transiently overexpressing *PpRAP2.4*. **C)** Expression patterns of genes related to anthocyanin biosynthesis in fruit transiently overexpressing *PpRAP2.4*. **D)** Transient silencing of *PpRAP2.4* in fruit. The empty vector (pTRV1 + pTRV2) was used as the negative control. Pear fruits were placed in darkness for 1 d and then treated with strong light for 6 d. **E)** Total anthocyanin content in fruit in which *PpRAP2.4* was transiently silenced. **F)** Expression patterns of genes related to anthocyanin biosynthesis in fruit in which *PpRAP2.4* was transiently silenced. Error bars represent the standard deviation of 3 biological replicates. Asterisks indicate significantly different values (*P < 0.05 and **P < 0.01).

To further characterize the PpRAP2.4 function related to anthocyanin biosynthesis, bagged mature “Hongzaosu” pear fruits (with no anthocyanin biosynthesis) were used to perform transient overexpression and VIGS analyses. After a 3-d light treatment, the red coloration of the pear fruit peel surrounding the injection site was more intense for the *PpRAP2.4*-overexpressing (*PpRAP2.4*-OX) fruit than for the control fruit (Fig. 4A). The anthocyanin content was much higher in the *PpRAP2.4*-OX fruit peel than in the control fruit peel (Fig. 4B). The *PpMYB114*, *PpMYB10*, *PpUFGT*, *PpANS*, *PpDFR*, *PpF3H*, *PpCHI*, and *PpCHS* expression levels were significantly higher in the *PpRAP2.4-OX* pear fruit peel than in the control samples (Fig. 4C). Additionally, the silencing of *PpRAP2.4* inhibited the red coloration and anthocyanin biosynthesis of pear fruit (Fig. 4, D and E). Moreover, the *PpMYB114*, *PpMYB10*, *PpUFGT*, *PpANS*, *PpDFR*, *PpF3H*, *PpCHI*, and *PpPAL* expression levels were downregulated in the *PpRAP2.4*-silenced fruit peel (Fig. 4F). These results imply that PpRAP2.4 induces anthocyanin biosynthesis in pear.

**Figure 5.**
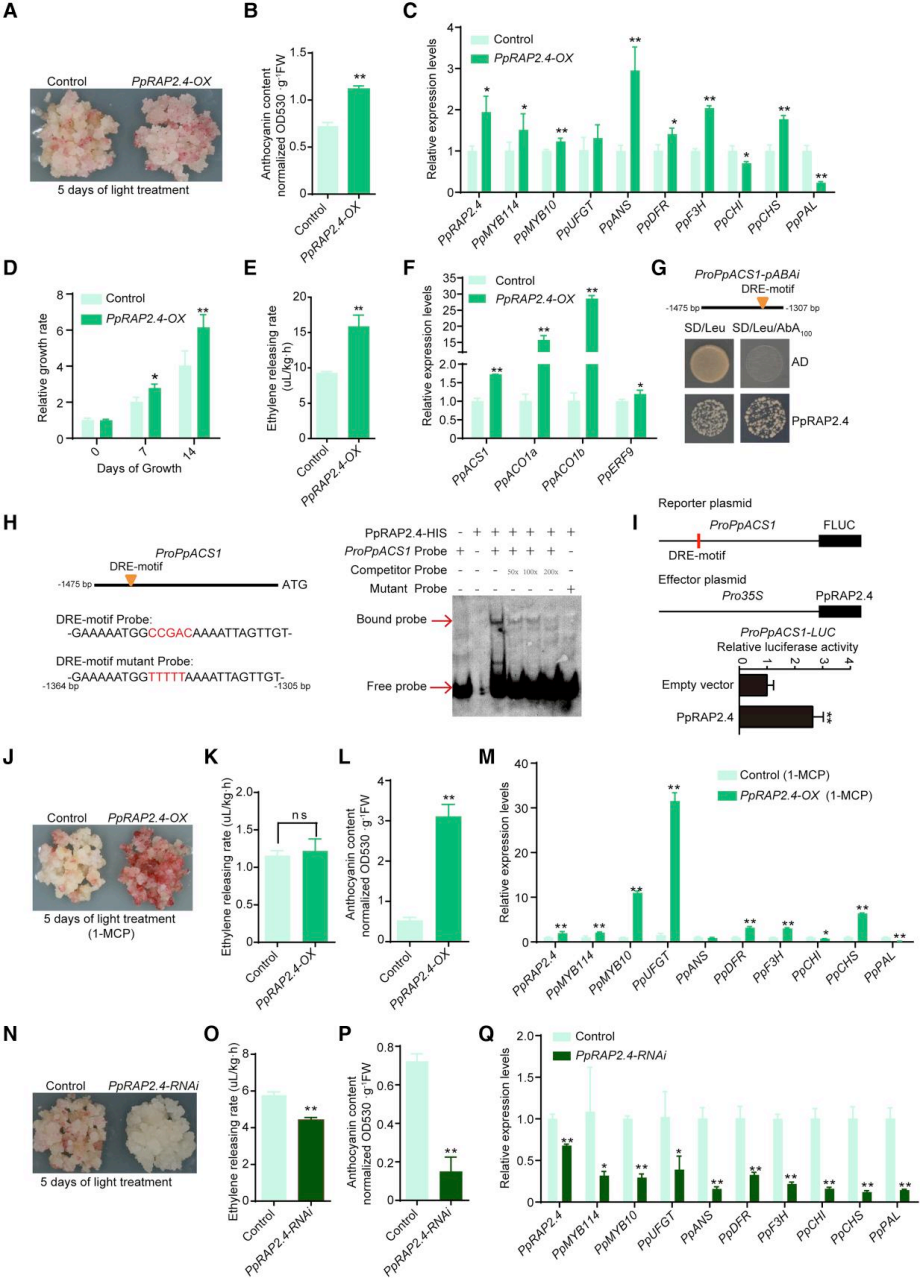
PpRAP2.4 positively regulates anthocyanin and ethylene biosynthesis. **A)** Overexpression of *PpRAP2.4* slightly induced the anthocyanin accumulation in pear calli. The calli transformed with the empty vector (pCAMBIA1301) were used as the negative control. Pear calli were incubated under strong light at 17°C for 5 d. **B)** Anthocyanin contents in *PpRAP2.4*-OX pear calli after light treatment. **C)** Expression levels of anthocyanin-related genes and *PpRAP2.4* in *PpRAP2.4*-OX pear calli after light treatment. **D)** Relative growth rate of *PpRAP2.4*-OX and control pear calli over 2 wk. **E)** Ethylene releasing rate of *PpRAP2.4*-OX and control pear calli. **F)** Expression levels of key ethylene biosynthetic genes (*PpACS1* and *PpACO1*) and *PpERF9* in *PpRAP2.4*-OX and control pear calli. **G)** A Y1H assay revealed that PpRAP2.4 can bind directly to the *PpACS1* promoter. **H)** An EMSA revealed that PpRAP2.4 can bind directly to the DRE motif of the *PpACS1* promoter. The hot probe was a biotin-labeled fragment of the *PpACS1* promoter containing the DRE motif, whereas the competitor probe was an unlabeled probe (50-, 100-, and 200-fold molar excess). The mutant cold probe was the same as the labeled hot probe but with CCGAC mutated to TTTTT. The His-tagged PpRAP2.4 protein was purified. **I)** A dual-luciferase assay indicated that PpRAP2.4 activates the *PpACS1* promoter in vivo. The promoter of *PpACS1* was cloned into the pGreenII 0800-LUC (firefly luciferase) vector, and the full-length CDS of *PpRAP2.4* was cloned into the pGreenII 0029 62-SK vector. The empty vector of pGreenII 0029 62-SK was used as control. The relative luciferase activity was analyzed. **J** and **L)** The 1-MCP treatment significantly induced the **J)** red coloration and increased the **L)** total anthocyanin content in *PpRAP2.4*-OX pear calli. **K)** The 1-MCP treatment decreased the ethylene release rate in *PpRAP2.4*-OX and control pear calli. **M)** Expression levels of anthocyanin-related genes and *PpRAP2.4* in *PpRAP2.4*-OX pear calli after the 1-MCP treatment. **N** to **P)** Silencing of *PpRAP2.4* inhibited the red coloration **N)**, ethylene releasing rate **O)**, and total anthocyanin contents **P)** in pear calli. **Q)** Expression levels of *PpRAP2.4* and anthocyanin-related genes in *PpRAP2.4*-RNAi pear calli and control pear calli (empty vector) after the light treatment. Error bars represent the standard deviation of 3 biological replicates. Asterisks indicate significantly different values (*P < 0.05 and **P < 0.01).

An examination of *PpRAP2.4*-OX transgenic pear calli in a coloration assay revealed that *PpRAP2.4* overexpression only slightly increased the accumulation of anthocyanins and the expression levels of anthocyanin-related genes after a 5-d light treatment (Fig. 5, A to C). Compared with the control calli, the *PpRAP2.4-OX* calli had a higher growth rate and ethylene release rate (Fig. 5, D and E). The expression levels of key ethylene biosynthetic genes (*PpACS1*, *PpACO1a*, and *PpACO1b*) were much higher in *PpRAP2.4-OX* pear calli than in the control calli (Fig. 5F), indicating that PpRAP2.4 induced ethylene production by transcriptionally regulating ethylene biosynthetic genes.

Analysis of the *PpACS1*, *PpACO1a*, and *PpACO1b* promoters detected a DRE motif in the *PpACS1* promoter (Fig. 5G). The Y1H assay and EMSA results demonstrated that PpRAP2.4 can bind to the DRE motif of the *PpACS1* promoter (Fig. 5, G and H). A dual-luciferase assay in *N. benthamiana* leaves verified that PpRAP2.4 can activate the *PpACS1* promoter (Fig. 5I). These results indicate that PpRAP2.4 can enhance the transcription of *PpACS1* by binding to its promoter, ultimately resulting in increased ethylene production. Considering that *PpERF9* was induced by ethylene signaling, we further analyzed the expression of *PpERF9* in *PpRAP2.4-OX* pear calli. The result showed that *PpERF9* was slightly upregulated in *PpRAP2.4-OX* pear calli (Fig. 5F), which repressed the effect of *PpRAP2.4* on promoting anthocyanin biosynthesis.

Considering that PpRAP2.4 induces ethylene biosynthesis, which may in turn inhibit anthocyanin biosynthesis, we treated *PpRAP2.4*-OX pear calli with 1-MCP to suppress ethylene production (Fig. 5J). The ethylene release rate decreased, with no significant difference between the *PpRAP2.4-OX* and control pear calli after the 1-MCP treatment (Fig. 5K). The *PpRAP2.4*-OX pear calli turned red and accumulated large amounts of anthocyanin after a 5-d light treatment, whereas the control pear calli were slightly pale red and accumulated less anthocyanin (Fig. 5, J and L). The *PpRAP2.4*, *PpMYB114*, *PpMYB10*, *PpUFGT*, *PpDFR*, *PpF3H*, and *PpCHS* expression levels were significantly higher in the *PpRAP2.4*-OX pear calli than in the control calli (Fig. 5M). Furthermore, in the *PpRAP2.4*-RNAi pear calli, red coloration was inhibited and anthocyanin biosynthesis-related gene expression as well as the ethylene release rate decreased (Fig. 5 N to Q). These observations suggest that PpRAP2.4 rapidly induces anthocyanin biosynthesis by activating *PpMYB114* expression via binding to the GCC-box of its promoter when ethylene production is inhibited (Figs. 3 to 5).

### PpERF9 inhibits *PpMYB114* expression by transcriptionally repressing *PpRAP2.4*

**Figure 6.**
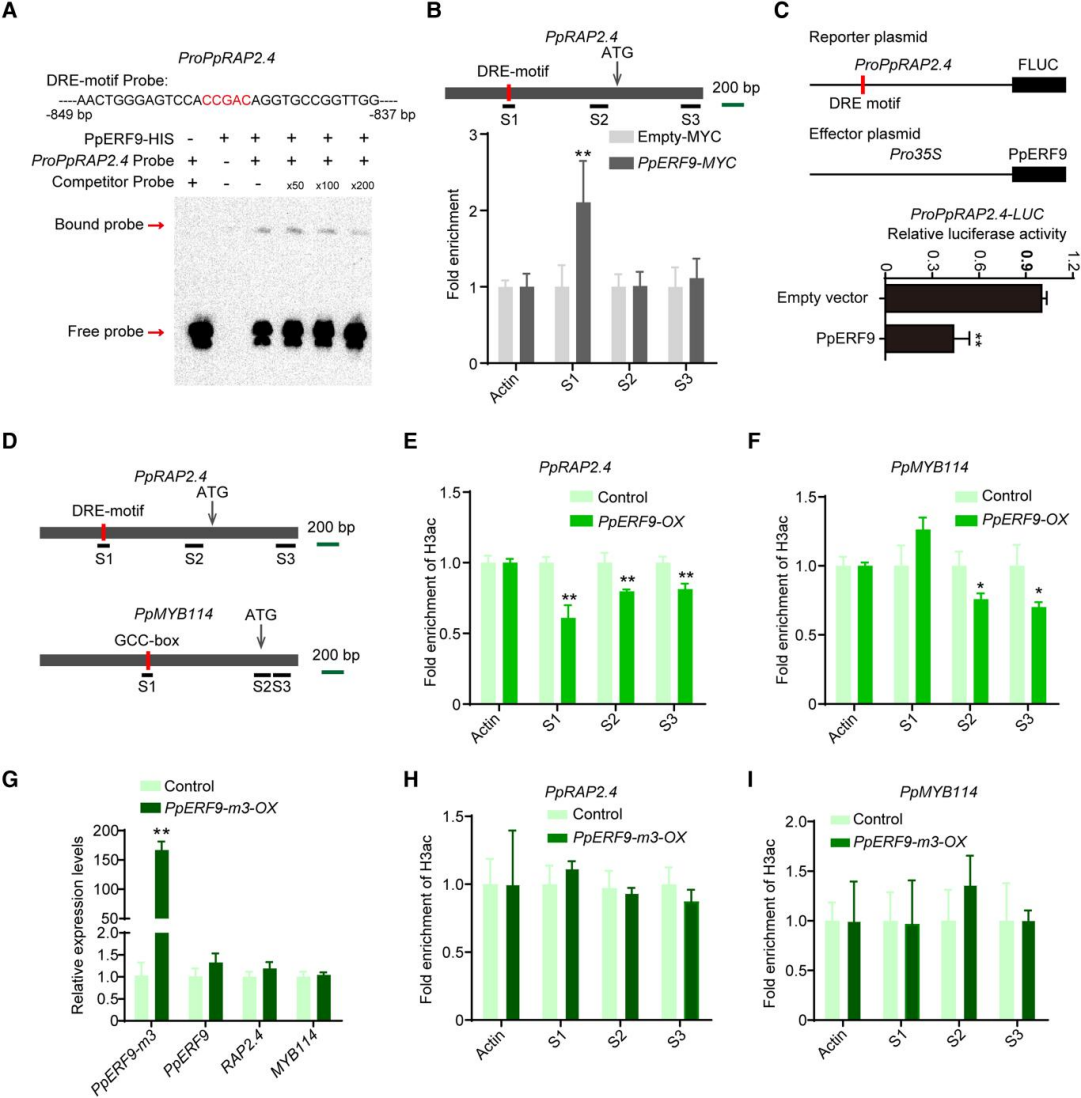
PpERF9 represses *PpRAP2.4* transcription and the suppressive effect of PpERF9 on anthocyanin biosynthesis depends on histone deacetylation. **A)** An EMSA indicated that PpERF9 can bind directly to the *PpRAP2.4* promoter. The hot probe was a biotin-labeled fragment of the *PpRAP2.4* promoter containing the DRE motif, whereas the competitor probe was an unlabeled probe (50-, 100-, and 200-fold molar excess). The His-tagged PpRAP2.4 protein was purified. **B)** A ChIP-qPCR assay revealed the in vivo binding of PpERF9 to the *PpRAP2.4* promoter. Cross-linked chromatin samples were extracted from *PpERF9-MYC*-overexpressing pear calli and then precipitated with an anti-MYC antibody. Eluted DNA was used to amplify the sequences surrounding the binding site by qPCR. Three regions (S1 to S3) were examined. Pear calli overexpressing the empty-MYC sequence were used as the negative control. The ChIP assay was repeated 3 times, and the enriched DNA fragments in each ChIP were used as 1 biological replicate for the qPCR. **C)** A dual-luciferase assay demonstrated that PpERF9 directly inhibits *PpRAP2.4* promoter activity in vivo. **D)** Schematic diagram of the primers used in the ChIP-qPCR assay. **E)** ChIP-qPCR results reflecting the H3ac levels at the *PpRAP2.4* locus in control and *PpERF9-OX* pear calli. **F)** ChIP-qPCR results reflecting the H3ac levels at the *PpMYB114* locus in control and *PpERF9-OX* pear calli. **G)** Expression levels of *PpERF9-m3*, *PpERF9*, *PpRAP2.4*, and *PpMYB114* in *PpERF9-m3*-OX pear calli and control pear calli after light treatment. Pear calli were incubated under strong light at 17°C for 5 d. **H)** ChIP-qPCR results reflecting the H3ac levels at the *PpRAP2.4* locus in control and *PpERF9-m3-OX* pear calli. **I)** ChIP-qPCR results reflecting the H3ac levels at the *PpMYB114* locus in control and *PpERF9-m3-OX* pear calli. Cross-linked chromatin samples were extracted from control, *PpERF9*-OX and *PpERF9-m3-OX* pear calli and then precipitated with an anti-H3ac antibody. Error bars represent the standard deviation of 3 biological replicates. Asterisks indicate significantly different values (*P < 0.05 and **P < 0.01).

We analyzed the potential binding sites of ethylene signaling-related genes and detected a DRE motif in the *PpRAP2.4* promoter (Fig. 6A), suggesting that ethylene might inhibit *PpRAP2.4* expression via repressive ERF TFs. We then investigated whether PpERF9 can repress *PpRAP2.4* promoter activity. The EMSA results indicated that PpERF9 can bind to the DRE motif of the *PpRAP2.4* promoter (Fig. 6A). A ChIP-qPCR assay revealed that PpERF9 significantly increased the PCR-based detection of the S1 region of the *PpRAP2.4* promoter containing the DRE motif, but not the S2 and S3 regions of the promoter; these observations clarified the binding of PpERF9 to the *PpRAP2.4* promoter in vivo (Fig. 6B). Our dual-luciferase assay showed that PpERF9 can substantially inhibit the *PpRAP2.4* promoter activity (Fig. 6C). Furthermore, the expression of *PpRAP2.4* was inhibited in *PpERF9*-OX pear fruit peel and calli (Fig. 2, C and I). These results indicate that PpERF9 inhibits the transcription of *PpRAP2.4* by binding to the DRE motif of its promoter. Thus, PpERF9 can directly inhibit the expression of *PpMYB114* by binding to its promoter, while also inhibit the expression of *PpMYB114* through PpRAP2.4.

### PpERF9 inhibits the expression of *PpRAP2.4* and *PpMYB114* via histone deacetylation

The EAR motif is a typical repressive domain associated with histone deacetylation (Kagale and Rozwadowski 2011). Considering that there are 2 EAR motifs in PpERF9 (Fig. 1D), we hypothesized that the repression of *PpRAP2.4* and *PpMYB114* transcription by PpERF9 involves histone deacetylation. To test this hypothesis, we analyzed the histone acetylation levels of the control and *PpERF9*-OX pear calli (DMSO-treated pear calli) using an antibody specific for acetylated histone H3 (H3ac). The overexpression of *PpERF9* significantly decreased the H3ac level in pear calli (Supplemental Fig. S8, A and B).

Furthermore, treatment with 10 µM trichostatin-A (TSA) (dissolved in 1% DMSO), which is an effective HDAC inhibitor (Van Lint et al. 1996), increased the H3ac level in *PpERF9*-OX pear calli; there was no significant difference between the H3ac levels of the transgenic and control calli (Supplemental Fig. S8B). However, TSA did not affect the H3ac level in the control pear calli (Supplemental Fig. S8B), probably because the control pear calli had a sufficiently high histone acetylation levels unaffected by environmental factors. After the light treatment, the TSA treatment partly recovered the red coloration and anthocyanin accumulation of the *PpERF9*-OX pear calli (Supplemental Fig. S8C). These results suggest that PpERF9 inhibits anthocyanin biosynthesis partly via histone deacetylation.

To further confirm our hypothesis, we examined the histone acetylation levels of *PpRAP2.4* and *PpMYB114* in *PpERF9*-OX pear calli by conducting ChIP-qPCR assays using the anti-H3ac antibody. Previous studies verified that histone acetylation occurs in the proximal promoters and/or exons (Benhamed et al. 2006; Zhou et al. 2013). We analyzed the histone acetylation levels of the regions containing the PpERF9-binding site, the proximal promoters, and the first exon regions (Fig. 6D). The changes in the H3ac levels of the housekeeping gene *PpACTIN* were analyzed as a control. The fold enrichment of H3ac decreased significantly in the binding site, the proximal promoter and first exon region of *PpRAP2.4*, while the fold enrichment of H3ac decreased significantly in the proximal promoter and first exon region of *PpMYB114* in *PpERF9*-OX pear calli (Fig. 6, E and F). These findings verified that PpERF9 modulates the *PpRAP2.4* and *PpMYB114* expression levels via histone deacetylation to inhibit anthocyanin biosynthesis in red pear.

A previous study revealed that EAR motifs are crucial for histone deacetylation (Kagale and Rozwadowski 2011). In the current study, we mutated both EAR motifs in PpERF9 (i.e. PpERF9-m3) and constructed the 35S:PpERF9-m3 overexpression vector (Supplemental Fig. S9A). We transformed the vector into wild-type pear calli to produce *PpERF9-m3*-OX pear calli, and *PpERF9-m3* was upregulated more than 150 folds compared with control pear calli (Fig. 6G). Exposure of *PpERF9-m3*-OX pear calli to strong light with UV-B for 5 d led to the accumulation of slightly more anthocyanin compared with control (Supplemental Fig. S9, A and B). Reverse transcription quantitative PCR (RT-qPCR) analysis indicated that overexpression of *PpERF9-m3* induced the expression of most anthocyanin biosynthetic genes (*PpMYB10*, *PpUFGT*, *PpANS*, *PpDFR*, *PpF3H*, *PpCHI*, and *PpCHS*) (Supplemental Fig. S9C). Meanwhile, the expression of *PpRAP2.4* and *PpMYB114* showed no significant differences compared with control pear calli (Fig. 6G). These results suggest that EAR motifs are required for the inhibition of anthocyanin biosynthesis by PpERF9 in pear.

Furthermore, we also examined the H3ac levels of *PpRAP2.4* and *PpMYB114* in *PpERF9-m3*-OX pear calli by performing ChIP-qPCR assays. The H3ac levels in the *PpRAP2.4* and *PpMYB114* promoters did not differ significantly between the control and *PpERF9-m3*-OX pear calli (Fig. 6, H and I), which indicated that EAR motifs in PpERF9 are necessary for histone deacetylation.

### PpERF9 recruits PpTPL1 via EAR motifs

**Figure 7.**
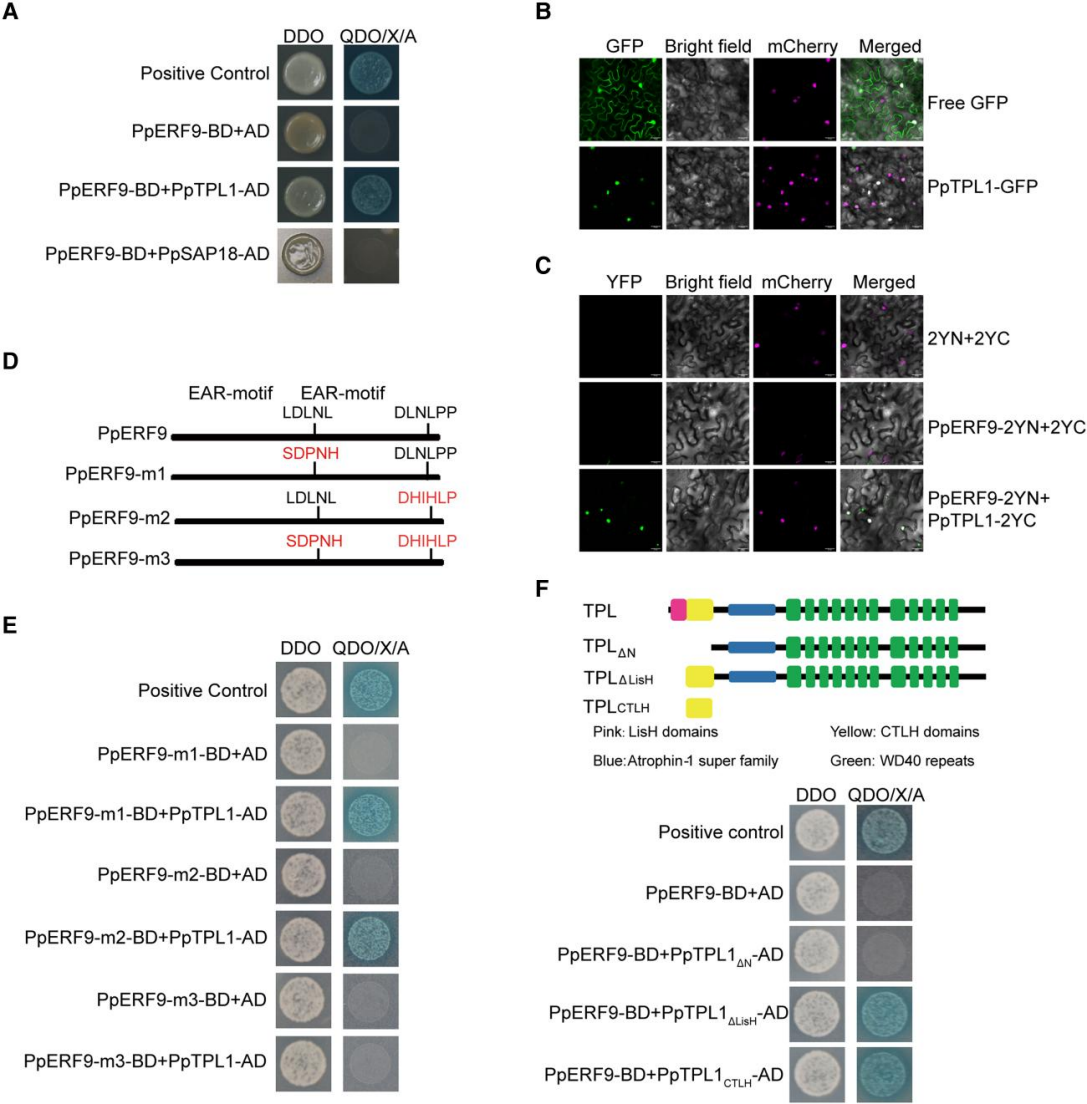
PpERF9 interacts with PpTPL1. **A)** Interaction between PpERF9 and PpTPL1 in a yeast two-hybrid assay, with pGADT7-T and pGBKT7-53 as positive controls. DDO, SD/−Trp/−Leu medium; QDO/X/A, SD/−Trp/−Leu/−Ade/−His medium with X-α-gal and Aureobasidin A. **B)** Subcellular localization of PpTPL1 in *N. benthamiana* leaf cells. **C)** Physical association between PpTPL1 and PpERF9 confirmed by a BiFC assay. Bars = 25 µm. **D)** Schematic diagram of the domain structures of the PpERF9 protein in the wild-type control and in the generated mutants. **E)** Yeast two-hybrid assays indicated that PpTPL1 interacts with either EAR motif in PpERF9. A mutation to only 1 of the EAR motifs in PpERF9 did not affect the interaction between PpERF9 and PpTPL1. However, the interaction was eliminated when both EAR motifs were mutated. **F)** Schematic diagram of the domain structures of the PpTPL1 protein in the wild-type control and in the generated mutants. Yeast two-hybrid assays indicated that PpERF9 interacts with the CTLH domain of PpTPL1.

The EAR motif reportedly mediates transcriptional repression via the recruitment of co-repressors, including TPL and SAP18, which are associated with histone deacetylation (Kagale and Rozwadowski 2011). We cloned the orthologs of *AtTPL1* and *AtASP18* in “Hongzaosu” pear fruit, namely, *PpTPL1* and *PpSAP18*. To confirm whether PpERF9 interacts with these 2 co-repressors, a yeast two-hybrid assay was performed. The assay results revealed that PpERF9 can interact with PpTPL1, but not with PpSAP18 (Fig. 7A). The subcellular localization analysis detected PpTPL1 in the nucleus (Fig. 7B). In the bimolecular fluorescence complementation (BiFC) assay, a strong YFP signal was detected in the nucleus when PpERF9-2YN and PpTPL1-2YC were transiently expressed in *N. benthamiana* leaves (Fig. 7C). Accordingly, our data suggest that PpERF9 interacts directly with PpTPL1.

To determine whether the 2 EAR motifs of PpERF9 are responsible for the interaction with PpTPL1, we analyzed the effects of mutating each EAR motif (Fig. 7D). When one of the EAR motifs was mutated (PpERF9-m1 or PpERF9-m2), PpERF9 was still able to interact with PpTPL1 (Fig. 7E). However, when both EAR motifs were mutated (PpERF-m3) (Fig. 7D), PpERF9 was unable to interact with PpTPL1 (Fig. 7E), suggesting that the interaction between PpERF9 and PpTPL1 depends on either EAR motif.

A structural analysis of PpTPL1 indicated the protein contains a characteristic LisH domain, a C-terminal lissencephaly homology (CTLH) domain, and C-terminal WD40 repeats (Fig. 7F). The deletion of the N-terminal region (containing the LisH and CTLH domains) eliminated the interaction between PpERF9 and PpTPL1 (Fig. 7F). Removing only the LisH domain did not affect the interaction between PpERF9 and PpTPL1, while the CTLH domain alone showed physical interaction with PpERF9 (Fig. 7F), indicating the CTLH domain is sufficient for the interaction between the 2 proteins. Our results indicate that the EAR motif and the CTLH domain are necessary for the interaction between PpERF9 and PpTPL1.

### PpTPL1 negatively regulates anthocyanin biosynthesis in pear calli

**Figure 8.**
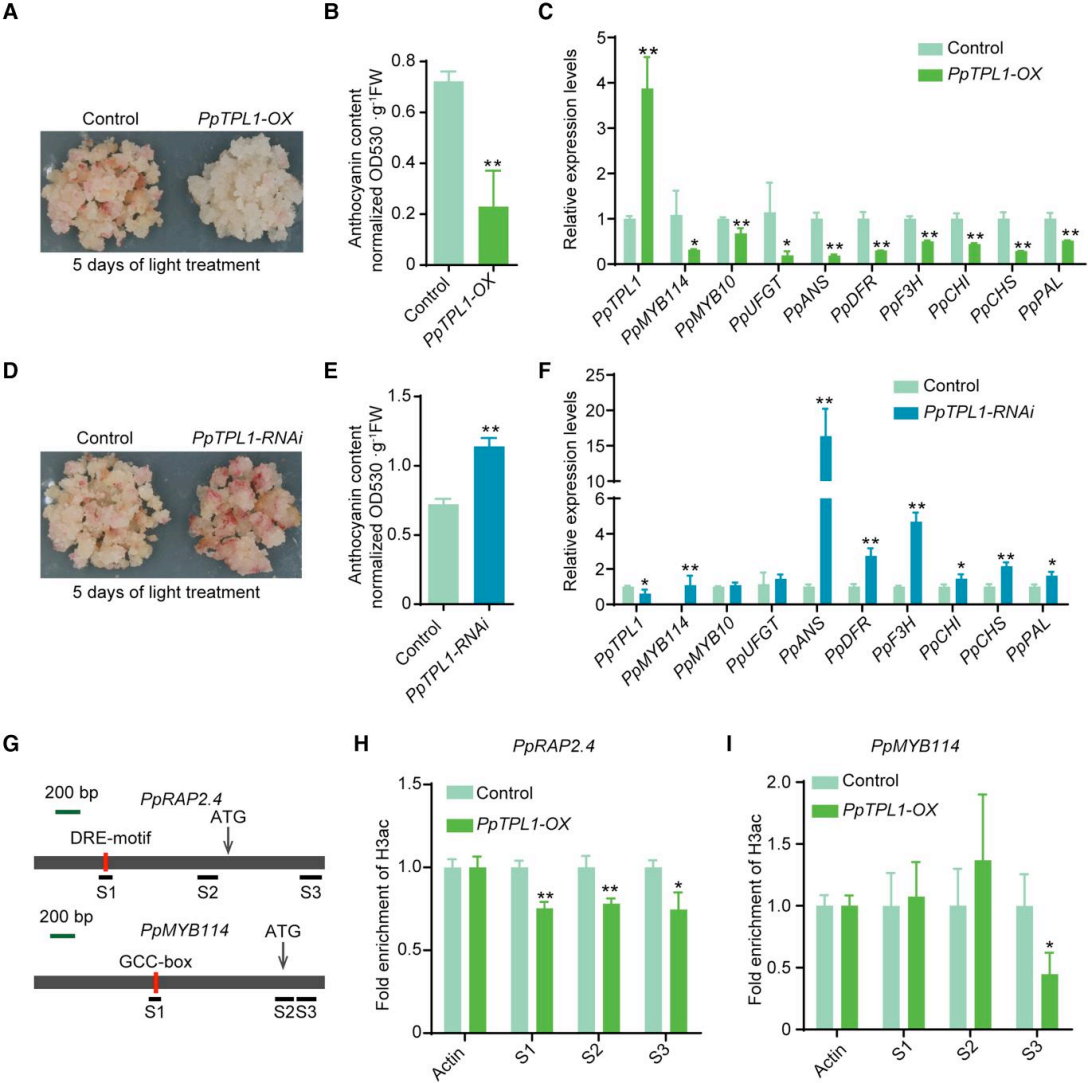
Functional analysis of PpTPL1 in pear calli. **A)** The overexpression of *PpTPL1* significantly inhibited the anthocyanin accumulation in pear calli. The calli transformed with the empty vector (pCAMBIA1301) were used as the negative control. Pear calli were incubated under strong light at 17°C for 5 d. **B)** Total anthocyanin contents in *PpTPL1*-OX pear calli after the light treatment. **C)** Expression levels of *PpTPL1* and anthocyanin-related genes in *PpTPL1*-OX pear calli after the light treatment. **D)** The silencing of *PpTPL1* induced the anthocyanin accumulation in pear calli. **E)** Total anthocyanin contents in *PpTPL1*-RNAi pear calli after the light treatment. **F)** Expression levels of *PpTPL1* and anthocyanin-related genes in *PpTPL1*-RNAi pear calli after the light treatment. **G)** Schematic diagram of the primers used in the ChIP-qPCR assay. **H)** ChIP-qPCR results reflecting the H3ac levels at the *PpRAP2.4* locus. **I)** ChIP-qPCR results reflecting the H3ac levels at the *PpMYB114* locus. Cross-linked chromatin samples were extracted from *PpTPL1*-OX pear calli and then precipitated with an anti-H3ac antibody. Error bars represent the standard deviation of 3 biological replicates. Asterisks indicate significantly different values (*P < 0.05 and **P < 0.01).

To analyze the PpTPL1 function related to pear anthocyanin biosynthesis and coloration, we generated *PpTPL1*-overexpressing (*PpTPL1*-OX) transgenic pear calli. The control pear calli (carrying the empty vector) and the *PpTPL1*-OX pear calli were then exposed to strong white light with UV-B. A 5-d light treatment caused the control pear calli to turn red and accumulate large amounts of anthocyanin, whereas anthocyanins accumulated at lower levels in the *PpTPL1*-OX pear calli (Fig. 8, A and B). A qPCR analysis indicated that the overexpression of *PpTPL1* downregulated the expression of *PpMYB114*, *PpMYB10*, *PpANS*, *PpDFR*, *PpF3H*, *PpCHI*, *PpCHS*, and *PpPAL* (Fig. 8C). Furthermore, we generated *PpTPL1*-RNAi pear calli to verify PpTPL1 function (Fig. 8D). The results demonstrated that silencing *PpTPL1* induced the red coloration and anthocyanin biosynthesis of pear calli in response to a 5-d light treatment (Fig. 8, D and E). According to a gene expression analysis, silencing *PpTPL1* upregulated the expression of *PpMYB114*, *PpANS*, *PpDFR*, *PpF3H*, *PpCHI*, *PpCHS*, and *PpPAL* (Fig. 8F). These findings reflected the negative effects of PpTPL1 on the anthocyanin biosynthesis and red coloration of pear.

We further analyzed the histone acetylation levels of *PpRAP2.4* and *PpMYB114* in *PpTPL1*-OX pear calli by conducting ChIP-qPCR assays using the anti-H3ac antibody. The fold enrichment of H3ac decreased significantly in the binding site region (DRE motif region), the proximal promoter, and the exon region of *PpRAP2.4* in *PpTPL1*-OX pear calli (Fig. 8, G and H). Regarding *PpMYB114*, the fold enrichment of H3ac decreased significantly in the first exon region (Fig. 8, G and I). These results indicate that PpTPL1 inhibits the expression of *PpRAP2.4* and *PpMYB114* via histone deacetylation, suggesting that PpERF9 represses *PpRAP2.4* and *PpMYB114* expression by interacting with PpTPL1.

### Ethylene inhibits the histone acetylation of *PpMYB114* in the fruit peel

**Figure 9.**
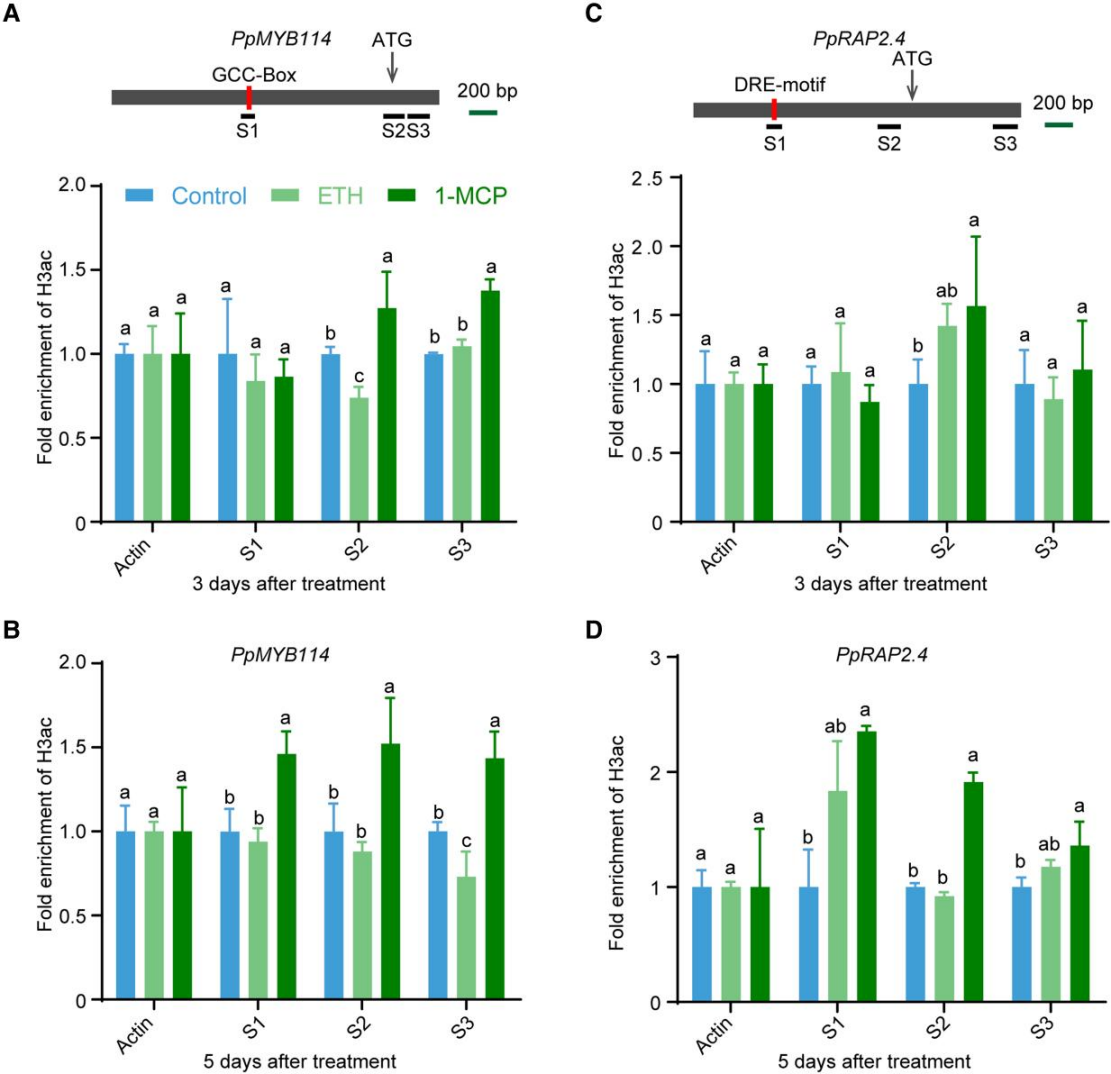
Ethylene inhibits pear anthocyanin biosynthesis via histone deacetylation. **A** and **B)** ChIP-qPCR results reflecting the H3ac levels at the *PpMYB114* locus in “Hongzaosu” pear fruit treated with ethephon and 1-MCP for 3 d **A)** and 5 d **B)**. **C** and **D)** ChIP-qPCR results reflecting the H3ac levels at the *PpRAP2.4* locus in “Hongzaosu” pear fruit treated with ethephon and 1-MCP for 3 d **C)** and 5 d **D)**. Cross-linked chromatin samples were extracted from “Hongzaosu” pear fruit peels treated with ethephon and 1-MCP and then precipitated with an anti-H3ac antibody. Error bars represent the standard deviation of 3 biological replicates. Different lowercase letters above the error bars indicate significant differences according to a one-way ANOVA with Tukey's test (*P* < 0.05).

Finally, we examined the H3ac levels of *PpMYB114* and *PpRAP2.4* in the fruit peel treated with ethephon and 1-MCP by performing a ChIP-qPCR assay involving the anti-H3ac antibody. In the *PpMYB114* locus, after a 3-d treatment, the H3ac level decreased in the S2 region of the ethephon-treated pear fruit peel but increased in the S2 and S3 regions of the 1-MCP-treated pear fruit peel (Fig. 9A). Following a 5-d treatment, the H3ac level decreased in the S3 region of the ethephon-treated pear fruit peel but increased in the S1 to S3 regions of the 1-MCP-treated pear fruit peel (Fig. 9B). Furthermore, in *PpRAP2.4* locus, the H3ac level showed no significant difference in the analyzed regions of the ethephon-treated pear fruit peel compared with control after 3-d and 5-d treatment but increased in the S2 region of the 1-MCP-treated pear fruit peel after a 3-d treatment and increased in the S1 to S3 regions of the 1-MCP-treated pear fruit peel after a 5-d treatment (Fig. 9, C and D). These results indicate that ethylene inhibits the expression of *PpMYB114* via histone deacetylation.

## Discussion

Anthocyanins are crucial for plant defenses against various biotic and abiotic stresses. In fruit, anthocyanins play important roles in signaling, such as serving as visual signals for seed dispersers in ripe fruits (Zoratti et al. 2014). However, most previous anthocyanin-related studies focused on the transcriptional activation of anthocyanin biosynthetic genes; there has been relatively little related research on transcriptional repression. We previously confirmed that ethylene inhibits anthocyanin biosynthesis of pear fruit with different genetic backgrounds (Ni et al. 2020), which is opposite with that in most fruit species, including apple, plum, and strawberry (Faragher and Brohier 1984; Villarreal et al. 2010; Cheng et al. 2016). However, the molecular mechanism underlying the transcriptional repression associated with ethylene-suppressed anthocyanin biosynthesis in pear has remained elusive. Here, we demonstrate that PpERF9, an EAR motif-containing AP2/ERF TF, which was induced by ethylene signaling, acts as a repressor of anthocyanin biosynthesis by forming a repressor complex with PpTPL1 to negatively regulate the transcription of anthocyanin biosynthetic key genes *PpRAP2.4* (a transcriptional activator of *PpMYB114*) and *PpMYB114* in pear.

### Ethylene-induced PpERF9 negatively regulates anthocyanin biosynthesis in pear fruit by inhibiting *PpRAP2.4* and *PpMYB114* transcription

In Arabidopsis, ethylene inhibits anthocyanin accumulation by suppressing the expression of *AtPAP1*, which positively regulates anthocyanin biosynthesis, and inducing the expression of the negative regulator *AtMYBL2* through the ethylene signaling pathway (Jeong et al. 2010). However, how ethylene signaling inhibits *AtPAP1* expression and induces *AtMYBL2* expression remains unknown. Similarly, we revealed that ethylene inhibits red pear anthocyanin biosynthesis by inducing the expression of *PpMYB140* via PpERF105 (Ni et al. 2021) and suppressing the expression of *PpMYB10* and *PpMYB114* (Supplemental Fig. S1D), which encode positive regulators of anthocyanin biosynthesis in pear (Feng et al. 2010; Yao et al. 2017). These findings suggest that the regulatory effects of ethylene on anthocyanin biosynthesis may be similar in Arabidopsis and pear fruit.

The ERF TFs are positive regulators of anthocyanin biosynthesis in various plant species. In apple, MdERF1B, MdERF3, and MdERF38 positively regulate the red coloration of fruit under various conditions (An et al. 2018b; Zhang et al. 2018b; An et al. 2020). In pear, PyERF3, Pp4ERF24, and Pp12ERF96 are positive modulators of anthocyanin biosynthesis (Yao et al. 2017; Ni et al. 2019). All of these ERF TFs promote anthocyanin biosynthesis by interacting with MYB TFs. However, there is relatively little available information regarding the repressive effects of ERF TFs on anthocyanin biosynthesis.

In the current study, we identified a repressive ERF TF (PpERF9) that responds positively to ethylene signaling (Fig. 1A). Additionally, PpERF9 inhibited the expression of *PpMYB114* by binding directly to the GCC-box of the promoter (Fig. 1, D to G). Moreover, PpRAP2.4 induced the expression of *PpMYB114* by binding to the GCC-box of its promoter (Fig. 3, D to F), whereas PpERF9 inhibited *PpRAP2.4* expression by binding to the DRE motif of its promoter (Fig. 6, A to C), indicative of an indirect regulatory pathway involving PpERF9, PpRAP2.4, and PpMYB114. These results reflect the varying regulatory effects (activation or repression) of different ERF TFs on anthocyanin biosynthesis. It is worth noting that PpERF9 and PpRAP2.4 functioned as a repressor and an activator of *PpMYB114* by binding to the same *cis*-element (GCC-box) in its promoter to regulate its expression. Furthermore, *PpERF9* and *PpRAP2.4* were positively and negatively regulated by ethylene signaling, respectively. When large amount of ethylene is present, the expression of *PpRAP2.4* is repressed by PpERF9, eliminating the competitive binding of PpERF9 and PpRAP2.4 to the *PpMYB114* promoter. Thus, ethylene signaling can negatively regulate the transcription of *PpMYB114* efficiently through PpERF9-PpMYB114 or PpERF9-PpRAP2.4-PpMYB114 pathways.

In this study, *PpRAP2.4*-OX pear calli had a significantly higher growth rate and ethylene release rate than the control calli (Fig. 5, D to F). Subsequent analyses showed that PpRAP2.4 can induce ethylene production by binding directly to the *PpACS1* promoter (Fig. 5, G to I). The ERF TFs are involved in the regulation of the ethylene biosynthesis pathway in many plant species. In apple, MdERF2 and MdERF3 negatively and positively, respectively, regulate ethylene biosynthesis by binding directly to the *MdACS1* promoter (Li et al. 2016). In banana (*Musa acuminata*), MaERF9 and MaERF11 bind directly to the *MaACO1* promoter to regulate gene expression (Xiao et al. 2013; Han et al. 2016). Hence, ethylene inhibits *PpRAP2.4* expression, but PpRAP2.4 induces ethylene biosynthesis by activating the *PpACS1* promoter, which forms a negative feedback regulatory pathway. Moreover, PpRAP2.4 induces anthocyanin biosynthesis by regulating *PpMYB114* transcription. This regulatory effect may prevent the over-accumulation of anthocyanins in the absence of ethylene and balance the production of secondary metabolites.

### The EAR motif-containing Tf-TPL complex is involved in the repression of gene expression associated with different physiological processes

In plants, there is a balance between the activation and repression of gene expression mediated by most plant hormones (including ethylene) (Zhang et al. 2018a). To the best of our knowledge, the mechanisms for gene activation are better characterized than the mechanisms for gene repression. In the ethylene signaling pathway, ERF TFs can function as transcriptional activators or repressors (Fujimoto et al. 2000). Generally, the repressive ERF TFs contain EAR motifs, which are responsible for suppressing gene expression (Ohta et al. 2001; Kagale and Rozwadowski 2011).

In Arabidopsis, Class II ERFs, which are characterized by their EAR motifs (Ohta et al. 2001), are involved in the responses to pathogens, ethylene, JA, ABA, and leaf senescence (McGrath et al. 2005; Nasir et al. 2005; Song et al. 2005; Yang et al. 2005; Koyama et al. 2013). Additionally, EAR motif-mediated transcriptional repression is associated with co-repressors, including TPL, SAP18, and HDAC. In Arabidopsis, AtERF3 and AtERF4, which are active repressors, physically interact with AtSAP18, which in turn interacts with AtHDA19 to form a repression complex (Song and Galbraith 2006). Furthermore, AtERF7, which is another EAR motif-containing Class II ERF, recruits AtHDA19 by interacting with AtSIN3, thereby decreasing the sensitivity of guard cells to ABA and increasing transpirational water loss (Song et al. 2005). In apple, MdERF4 interacts with MdTPL4 to inhibit fruit ripening and ethylene production, and the transcriptional repression by MdERF4 is modulated by MdTPL4 (Hu et al. 2020). In banana, MaERF11 recruits the HDAC MaHDA1 to repress the expression of *MaACO1* and *expansins* to regulate fruit ripening (Han et al. 2016).

In the current study, we revealed that PpERF9 physically interacts with PpTPL1, but not with PpSAP18 (Fig. 7). Mutations to both EAR^PpERF9^ motifs completely abolished the interaction between PpERF9 and PpTPL1 (Fig. 7, D and E), indicating that the EAR^PpERF9^ motifs are necessary and sufficient for the interaction between the co-repressors, which is consistent with the results of a previous study (Pauwels et al. 2010). Earlier research confirmed that the CTLH domain is also necessary and sufficient for the interaction involving the EAR motif (Szemenyei et al. 2008). Our results were in accordance with this finding (Fig. 7F). Furthermore, PpERF9 clustered together with AtERF3/4/7/8/9/10/11/12 and MdERF4 in the phylogenetic tree (Supplemental Fig. S3), indicating that the interaction between EAR motif-containing TFs and co-repressors like TOPLESS is conserved among plant species.

In Arabidopsis, TPL negatively regulates JA-induced anthocyanin accumulation (Li et al. 2020). When the JA level is low, JAZ6/8 interacts with ECAP, which binds to TOPLESS-RELATED 2 (TPR2) to form the JET transcriptional repressor complex. The JET complex is further recruited by PAP1 or TT8/EGL3 to decrease the histone acetylation levels of anthocyanin biosynthetic structural genes, ultimately leading to inhibited anthocyanin biosynthesis (Li et al. 2020). Zheng et al. (2019) reported that in Arabidopsis, HAT1 can interact with PAP1, while also recruiting the TPL co-repressor to decrease the histone acetylation levels of anthocyanin biosynthetic structural genes, thereby inhibiting anthocyanin biosynthesis. However, whether the anthocyanin regulatory genes are affected by histone modifications remains unclear.

In the current study, PpERF9 was observed to bind directly to the *PpMYB114* and *PpRAP2.4* promoters (Fig. 1, D to G, Fig. 6, A to C) and recruit PpTPL1 to form a histone modification complex that suppresses the histone H3 acetylation of *PpRAP2.4* and *PpMYB114* (Figs. 6 to 8). The resulting inhibited expression of these genes contributes to the suppression of anthocyanin biosynthesis in the pear fruit peel. These findings indicate that EAR motif-containing TFs along with the TPL repressive complex have a universal role related to the repression of gene expression and physiological responses.

### Histone deacetylation contributes to ethylene-induced transcriptional repression

Ethylene is a gaseous plant hormone that regulates diverse growth and developmental processes, including fruit ripening, senescence, and coloration. A previous study indicated that of the ethylene-regulated genes, approximately half are downregulated and half are upregulated (Chang et al. 2013). Earlier research also showed that histone acetylation and deacetylation are critical for ethylene responses.

Histone acetylation is always involved in ethylene-induced responses. For example, HDAC19 (AtRPD3A) positively regulates the expression of ethylene-responsive genes (*AtERF1* and ethylene-regulated *PATHOGENESIS-RELATED* genes) in Arabidopsis (Zhou et al. 2005). Furthermore, Wang et al. (2021) reported that EIN2-EIN3 increases the H3K14ac and H3K23ac levels to activate transcription. In contrast, histone deacetylation participates in ethylene-repressive responses. In the presence of ethylene, ENAP1 recruits 2 HDACs (STR1 and STR2) to remove the acetyl group from K9 on H3 tails and then maintain a low level of H3K9ac in the promoter regions of the ethylene-downregulated genes to repress their expression (Zhang et al. 2018a).

In the current study, we observed that ethylene signaling decreased the H3ac level in the proximal promoter and transcriptional start site of *PpMYB114* (Fig. 9). Additionally, the overexpression of *PpERF9* and *PpTPL1* decreased the H3ac levels at these loci (Figs. 6 and 8). Mutations to both EAR^PpERF9^ motifs eliminated the inhibition of anthocyanin biosynthesis as well as the histone deacetylation effect (Fig. 6, G to I). Furthermore, a TSA treatment partly recovered the anthocyanin accumulation of *PpERF9*-OX pear calli (Supplemental Fig. S8). These results indicate that the inhibition of pear anthocyanin biosynthesis mediated by ethylene is partly dependent on histone deacetylation. Thus, our data may be useful for further clarifying the histone modification-related ethylene response in plants.

**Figure 10.**
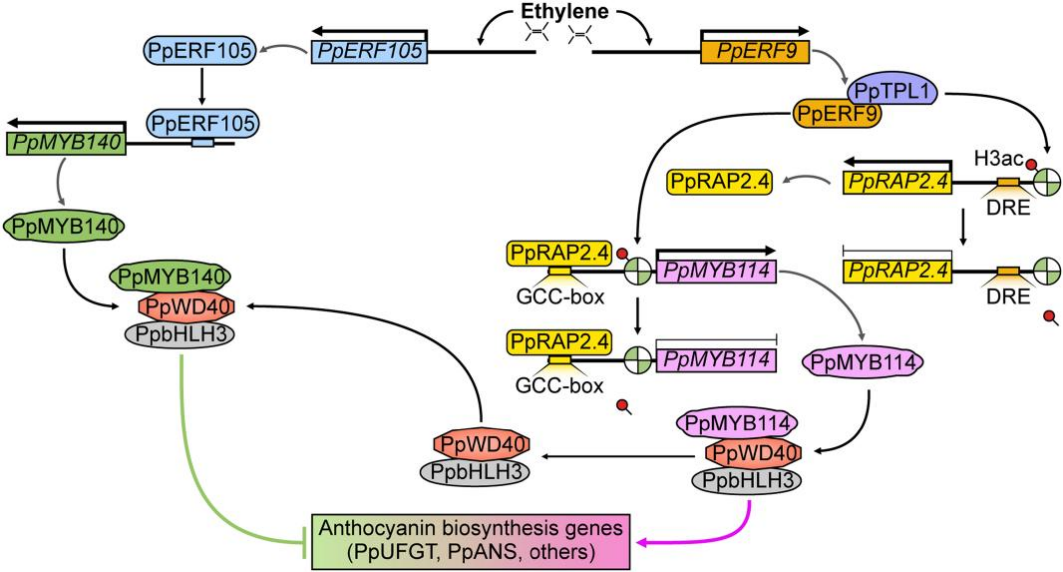
Proposed model for the ethylene-inhibited anthocyanin biosynthesis via the PpERF9-PpTPL1 co-repressor complex-mediated histone deacetylation in pear fruit. In the presence of ethylene, the expression of *PpERF9* is induced. Additionally, PpERF9 interacts with the PpTPL1 co-repressor to form a complex that removes the acetyl group on histone H3 and maintains a low H3ac level in the promoter regions of PpERF9-targeted genes (*PpRAP2.4* and *PpMYB114*) to repress their expression. On the one hand, PpERF9 binds to the *PpMYB114* promoter and inhibits its expression via PpERF9-PpTPL1-mediated histone deacetylation, directly inhibiting anthocyanin biosynthesis in pear fruit. On the other hand, PpERF9 inhibits the expression of *PpRAP2.4* via histone deacetylation. PpRAP2.4 induces the expression of *PpMYB114* by binding to its promoter. Thus, a PpERF9-PpRAP2.4-PpMYB114 regulatory pathway inhibits anthocyanin biosynthesis in pear fruit. Furthermore, ethylene signaling also induces the expression of *PpERF105*, which then activates *PpMYB140*, a transcriptional repressor of anthocyanin biosynthesis by competing with *PpMYB114* to form the MBW complex, which finally inhibits anthocyanin biosynthesis in pear fruit.

In summary, we revealed a PpERF9-TPL complex-mediated regulatory mechanism underlying ethylene-inhibited anthocyanin biosynthesis in pear fruit. In the presence of ethylene, the expression of *PpERF9* is induced. Additionally, PpERF9 interacts with the PpTPL1 co-repressor to form a complex that removes the acetyl group on histone H3 and maintains a low H3ac level in the promoter regions of PpERF9-targeted genes (*PpRAP2.4* and *PpMYB114*) to repress their expression. On the one hand, PpERF9 binds to the *PpMYB114* promoter and inhibits its expression via PpERF9-PpTPL1-mediated histone deacetylation, directly inhibiting anthocyanin biosynthesis in pear fruit. On the other hand, PpERF9 represses the expression of *PpRAP2.4* via histone deacetylation. PpRAP2.4 induces the expression of *PpMYB114* by binding to its promoter. Thus, the PpERF9-PpRAP2.4-PpMYB114 regulatory pathway inhibits anthocyanin biosynthesis in pear fruit. In conjunction with our previous study (Ni et al. 2021), ethylene signaling also induces the expression of *PpERF105*, which then activates *PpMYB140*, a transcriptional repressor of anthocyanin biosynthesis by competing with *PpMYB114* to form the MBW complex, which ultimately inhibits anthocyanin biosynthesis in pear fruit (Fig. 10). Our findings provide insight into the role of the ethylene signaling pathway in the regulation of anthocyanin biosynthesis. In addition, our data may shed light on the link between the chromatin state and hormone signaling and explain how plants respond to ethylene signals in various pathways.

## Materials and methods

### Plant materials and treatment

“Hongzaosu” (also called “Red Zaosu”) pear fruit (*Pyrus pyrifolia* × *Pyrus communis*) was collected from the orchard at the Institute of Horticulture, Henan Academy of Agricultural Sciences, Henan, China. All fruits were covered with lightproof double-layered paper bags at 15 d after full bloom (in March 2020). The bagged fruit was harvested about 140 d after full bloom (in July 2020) and immediately transported to the laboratory. The bagged fruit was randomly divided into 3 groups. The fruit was treated as previously described (Ni et al. 2020). Briefly, the fruit in 1 group was treated with 0.5 µL/L 1-MCP (SmartFresh) in sealed buckets for 16 h. The fruit in the second group were treated with 2 mM ethephon prepared in a solution containing 0.1% Tween-20 to promote absorption. The fruit in the third group served as the controls. The treated and control fruits were incubated for 7 d under continuous LED white light (60 µmol·m^−2^·s^−1^) in a growth chamber set at 17°C and 80% relative humidity. After measuring the fruit color, ethylene release rate, TSS, and firmness of each fruit, the exposed side of the fruit peel was collected, immediately frozen in liquid nitrogen, and stored at −80°C until used.

### Measurement of the total anthocyanin content

Anthocyanins were extracted from 0.1 g pear fruit peel or calli using a methanol:HCl (99:1, v/v) solution as previously described (Ni et al. 2017). The absorbance at 530, 620, and 650 nm was measured using the DU800 spectrophotometer (Beckman Coulter, Fullerton, CA, USA). The anthocyanin content was calculated using the following formula: [(A_530_ − A_650_) − 0.2 × (A_650_ − A_620_)]/0.1.

### RNA extraction and reverse transcription quantitative PCR

Total RNA was extracted from individual fruit peel samples according to a modified CTAB method (Zhang et al. 2012). First-strand cDNA was synthesized from 1 µg RNA using the PrimeScript RT reagent Kit with gDNA Eraser (Takara, Dalian, China). The qPCR analysis was conducted using TB Green Premix Ex Taq (Tli RNaseH Plus) (Takara) and the CFX Connect real-time PCR system (Bio-Rad, https://www.bio-rad.com/). The qPCR primers were designed using an online tool (https://www.ncbi.nlm.nih.gov/tools/primer-blast/) (Supplemental Table S1). The relative transcript levels were determined according to the 2^−ΔΔCt^ method (Livak and Schmittgen 2001). The pear *PpACTIN* gene (accession number: JN684184) was used for normalizing data.

### Sequence analysis and phylogenetic analysis

Protein sequence alignments were performed using MEGA version 5.0 software with default parameters. The phylogenetic tree was constructed via the maximum-likelihood method in MEGA version 5.0 software. The protein sequence alignments used to construct the phylogenetic tree are provided in Supplemental File S1, and the tree files are provided in Supplemental File S2.

### Subcellular localization analysis

The full-length complete coding sequences (CDSs) (without termination codons) of the target genes (*PpERF9*, *PpTPL1*, and *PpRAP2.4*) were independently cloned into the pCAMBIA1300 vector, which contained the CaMV 35S promoter and GFP gene, resulting in fusion genes driven by the 35S promoter. The intactness of the fusion protein is verified by immunoblotting. *Agrobacterium tumefaciens* EHA105 cells harboring the vectors were independently infiltrated into the leaves of *N. benthamiana* transgenic lines containing red fluorescent protein in the nuclei. The empty vector of pCAMBIA1300 was used as the negative control. The fluorescence was observed by confocal laser scanning microscopy (A1, Nikon, Tokyo, Japan).

### Trans-activation activity assay

A trans-activation activity assay was performed as previously described (Bai et al. 2019b). Briefly, the CDS of each gene (*PpERF9* and *PpRAP2.4*) was fused with the VP16 fragment and inserted into the pGBKT7 (BD) vector (gene-VP16-BD). The resulting vector and the positive (VP16-BD) and negative (BD) controls as well as gene-BD were independently transformed into yeast strain AH109 cells. Positive transformants were selected on a selective synthetic dextrose medium lacking tryptophan and histidine (SD/−Trp/−His). The β-galactosidase activity was measured as previously described (He et al. 2012). The enzyme activity was analyzed in 3 independent experiments, with at least 3 biological replicates per experiment. The primers used for constructing vectors are listed in Supplemental Data Set S1.

### Y1H and two-hybrid assays

Y1H assays were performed using the Matchmaker Gold Yeast One-Hybrid System Kit (Takara) according to the manufacturer's protocol. Briefly, gene (*PpMYB114*, *PpRAP2.4*, and *PpACS1*) promoter fragments were ligated into the pAbAi vector, whereas the TF gene CDSs were cloned into the pGADT7 vector (gene-AD). The gene-AD vectors were then used to transform Y1HGold cells harboring the pAbAi-bait and then screened on SD/−Leu/AbA medium. Yeast two-hybrid assays were performed using the Matchmaker Gold Yeast Two-Hybrid System Kit (Takara) according to the manufacturer's protocol.

### Dual-luciferase assay

The full-length CDS of each gene was cloned into the pGreenII 0029 62-SK vector (gene-SK). The gene (*PpMYB114*, *PpRAP2.4*, and *PpACS1*) promoter sequence was inserted into the pGreenII 0800-LUC vector (promoter-LUC). Both constructs were used to transform *A. tumefaciens* strain GV3101 cells harboring the pSoup vector. The firefly luciferase and *Renilla* luciferase activities were analyzed at 60 h after the infiltration using the Dual-Luciferase Reporter Assay System (Promega) and the GloMax96 Microplate Luminometer (Promega). Three independent experiments were performed to analyze the relative luciferase activities, with 6 biological replicates per experiment.

### Electrophoretic mobility shift assay

The DNA probes for the EMSA were prepared by annealing complementary oligonucleotides with biotin-labeled 3′ ends (95°C for 5 min and 70°C for 20 min). The EMSA was performed using the LightShift Chemiluminescent EMSA Kit (Thermo Scientific) as previously described (Bai et al. 2019b). The EMSA probes are listed in Supplemental Data Set S1.

### Transient transformation assay using pear fruit

The bagged mature red pear fruits (with no anthocyanin accumulation) were used for infiltrations. For the transient overexpression assay, the full-length CDS of the candidate gene (*PpERF9* and *PpRAP2.4*) was inserted into the pCAMBIA1301 vector for the subsequent expression under the control of the 35S promoter. Pear fruits were infiltrated with *A. tumefaciens* EHA105 cells containing the recombinant plasmid using a needle-free syringe. The pear fruits were then incubated in darkness firstly and then transferred to a growth chamber for an incubation under continuous white light (60 µmol·m^−2^·s^−1^) and UV-B light (1.1 µmol·m^−2^·s^−1^).

Regarding the VIGS assay, the gene fragment (∼200 to 300 bp) was amplified and cloned into the pTRV2 vector (gene-pTRV2). The resulting vector was inserted into *A. tumefaciens* GV3101 cells. The injection solution containing gene-pTRV2 or empty pTRV2 (control) was co-infiltrated with pTRV1 into red pear fruits, which were then incubated in darkness firstly and transferred to a growth chamber for an incubation under continuous white light (60 µmol·m^−2^·s^−1^) and UV-B light (1.1 µmol·m^−2^·s^−1^). For all the transient transformation assay, the regions surrounding the injection areas were collected for pigment and gene expression analyses.

### Genetic transformation of pear calli

Pear calli were transformed as previously described (Bai et al. 2019a). Briefly, pear calli were immersed for 12 min in a solution (Murashige and Skoog (MS) medium supplemented with 30 g/L sucrose) containing *A. tumefaciens* strain EHA105 cells harboring specific vectors (PpRAP2.4-pCAMBIA1301, PpERF9-pCAMBIA1301, or PpTPL1-pCAMBIA1301 for overexpression; empty pCAMBIA1301 as the negative control). The calli were placed on solid MS medium for a 3-d co-culture. The pear calli were screened on solid MS medium supplemented with specific antibiotics at 24°C in darkness for 1 mo. The transformed calli were subcultured every 2 to 3 wk. For the light treatment, freshly subcultured calli were exposed to continuous light in a growth chamber and examined daily. Regarding the 1-MCP treatment, freshly subcultured calli were placed in a clean sealed box containing 0.5 µL/L 1-MCP gas for 16 h. For the TSA treatment, TSA (dissolved in DMSO) was added to solid MS medium for a final concentration of 10 µmol/L. As a control, DMSO was added to the solid MS medium. Because TSA is unstable under light, fresh pear calli were placed on solid MS medium supplemented with TSA or DMSO and incubated under dark for 7 d, after which they were placed under light for an analysis of coloration.

### Measurement of the relative growth rate

To measure the pear calli growth rate, 1 g freshly subcultured *PpRAP2.4*-OX pear calli was used. An equal amount of pear calli transformed with the empty pCAMBIA1301 vector was used as the negative control. The pear calli were placed on solid MS medium supplemented with specific antibiotics and allowed to grow. The pear calli weight was measured every 7 d. Additionally, the calli were transferred to fresh MS medium weekly. The relative growth rate was calculated. The experiment was performed twice, with 3 biological replicates each time.

### ChIP assay

For the ChIP assays, pear calli were transformed with the recombinant PpERF9-MYC construct or the empty construct (MYC alone). Regarding the histone deacetylation analysis, *PpERF9*-OX and *PpTPL1*-OX (pCAMBIA1301) pear calli were used. The ChIP assays were performed as previously described, with some modifications (Han et al. 2016). For the cross-linking step, pear calli or fruit peels were treated with 1% formaldehyde (v/v) for 10 min. Next, the chromatin was extracted via sucrose gradient centrifugation. The chromatin DNA was sonicated for 30 min at 0°C (30 s with 30 s intervals) using the Bioruptor Plus device (Diagenode) to generate 200- to 500-bp fragments. The chromatin DNA fragments were incubated overnight with Anti-MYC (Sigma-Aldrich, 05-724) or anti-H3ac antibodies (Millipore, Catlog # 06-599/Lot # 2842168), after which the amount of immunoprecipitated chromatin was determined by qPCR. Each ChIP assay was repeated 3 times, and the enriched DNA fragments in each ChIP sample were used as 1 biological replicate for the qPCR analysis. The *PpACTIN* gene was used as the internal control for normalizing the ChIP enrichment signal in pear calli and fruit peels. The primers used for the ChIP-qPCR analysis are listed in Supplemental Table S2.

### Bimolecular fluorescence complementation assay

The full-length *PpTPL1* and *PpERF9* CDSs were inserted into the p2YN and p2YC vectors. *A. tumefaciens* GV3101 cells were transformed with the resulting plasmids according to a freeze–thaw method. Equal volumes of the different combinations were mixed for the infiltration of *N. benthamiana* leaves using a needle-free syringe. Leaves were co-infiltrated with PpERF9-2YN and 2YC as the negative control. The YFP signal was detected using a confocal laser scanning microscope (Nikon, Japan) 48 h later.

### Statistical analysis

Student's *t*-test and a one-way ANOVA (Tukey) were completed using the Statistical Product and Service Solutions program (SPSS Inc., Chicago, IL, USA). Detailed statistical analysis data are shown in Supplemental Data Set S2.

### Accession numbers

Sequence data from this article can be found in an online pear genome database (http://peargenome.njau.edu.cn): Pbr000398.1 (*PpERF9*), Pbr016049.2 (*PpRAP2.4*), Pbr042132.1 (*PpMYB114*), Pbr001905.1 (*PpACS1*), Pbr018800.1 (*PpSAP18*), Pbr029366.1 (*PpTPL1*), Pbr019531.1 (*PpCHS*), Pbr038148.1 (*PpCHI*), Pbr034840.1 (*PpF3H*), Pbr020145.1 (*PpDFR*), Pbr001543.2 (*PpANS*), Pbr039986.1 (*PpUFGT*), Pbr017379.1 (*PpbHLH3*), and Pbr016663.1 (*PpMYB10*).

## Supplementary Material

koad077_Supplementary_Data

## Supplemental data

The following materials are available in the online version of this article.

**Supplemental Figure S1.** Phenotypes of “Hongzaosu” pear fruit following ethephon and 1-MCP treatments and effects of ethephon and 1-MCP treatments on the ethylene release rate and expression levels of anthocyanin biosynthetic genes.

**Supplemental Figure S2.** Expression patterns of *PpMYB10*, *PpMYB114* and candidate *AP2/ERF* genes identified on the basis of RNA-seq data in “Hongzaosu” pear fruit after ethephon and 1-MCP treatments.

**Supplemental Figure S3.** Phylogenetic analysis of candidate ERF TFs from pear, apple, strawberry and Arabidopsis.

**Supplemental Figure S4.** Alignment of the cloned sequence and genomic sequence of Pbr000396.1.

**Supplemental Figure S5.** Comparison of protein sequences and domains of Pbr000396.1 in genome data and our cloned results and protein sequence and EAR motifs of PpERF9 (Pbr000398.1).

**Supplemental Figure S6.** Subcellular localization of PpERF9 in *N. benthamiana* leaf cells.

**Supplemental Figure S7.** A dual-luciferase assay demonstrated that PpERF9 and PpRAP2.4 have no effect on *PpMYB10* promoter activity.

**Supplemental Figure S8.** The suppressive effect of PpERF9 on anthocyanin biosynthesis depends on histone deacetylation.

**Supplemental Figure S9.** Functional analysis of PpERF9-m3 in “Clapp's Favorite” pear calli.

**Supplemental Table S1**. Primers for qPCR analysis in this study.

**Supplemental Table S2**. Primers for ChIP-qPCR analysis in this study.

**Supplemental Data Set S1**. Primers used for constructing vectors or EMSA in this study.

**Supplemental Data Set S2**. Summary of statistical tests.

**Supplemental File S1**. Protein sequences used to generate phylogenetic tree.

**Supplemental File S2**. Phylogenetic tree of ERF proteins in Newick format.
