# Supplementary material for: The ethylene-responsive transcription factor PpERF9 represses PpRAP2.4 and PpMYB114 via histone deacetylation to inhibit anthocyanin biosynthesis in pear
Source: Plant Cell. 2023 Mar 14;35(6):2271–92. doi: 10.1093/plcell/koad077 (PMC10226596; doi:10.1093/plcell/koad077)
Supplement: koad077_Supplementary_Data [file koad077_supplementary_data.zip › Supplemental Figures and Tables.pdf]

SUPPLEMENTAL DATA

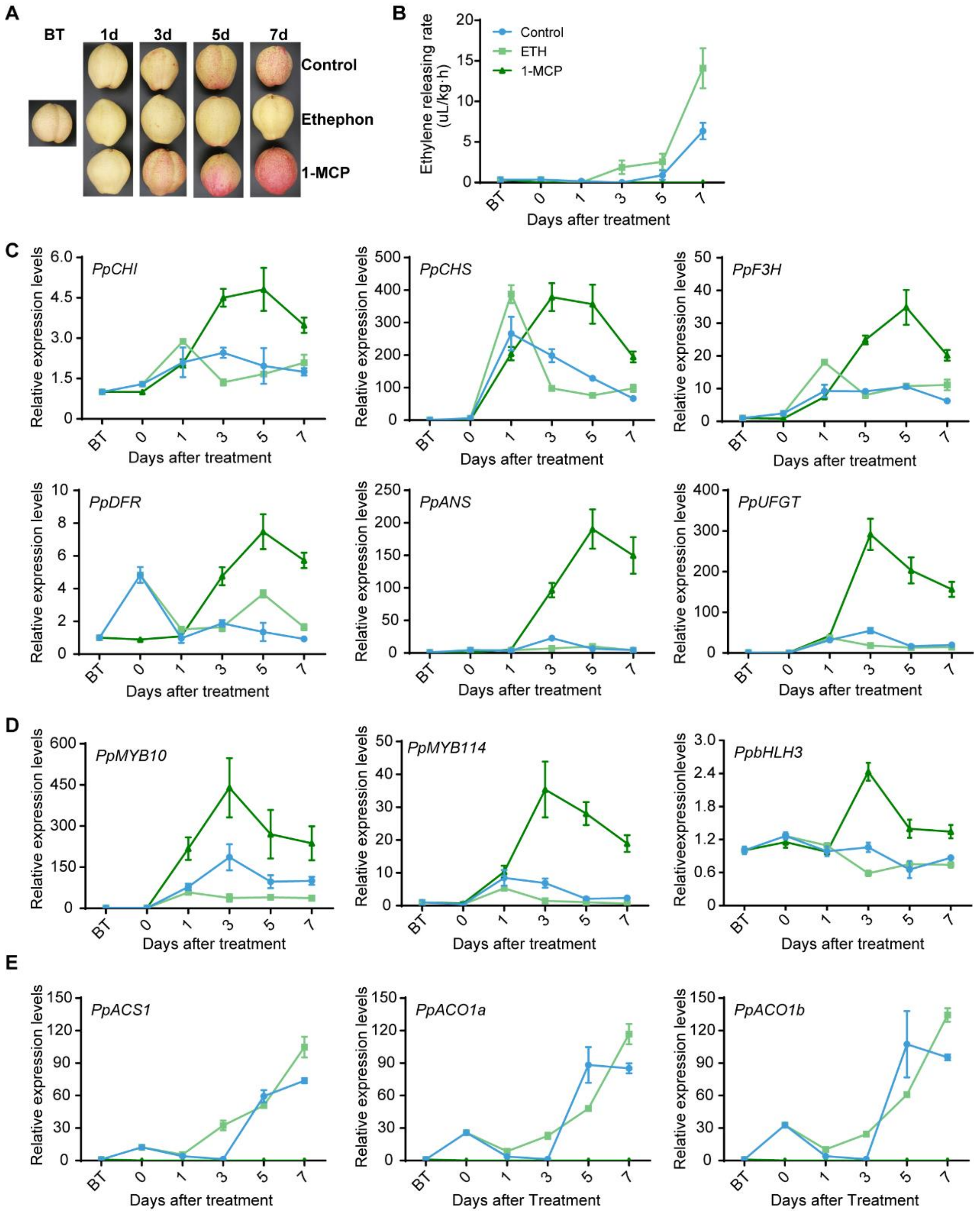

Supplemental Figure S1 (Supports Figures 1 and 3). (A) Phenotypes of 'Hongzaosu' pear fruit following ethephon and 1-MCP treatments. (B) Effects of ethephon and 1-MCP treatments on the ethylene releasing rate. (C-E) Relative expression levels of anthocyanin biosynthetic structural genes, anthocyanin biosynthetic regulatory genes, and ethylene biosynthetic key genes in 'Hongzaosu' pear fruit after ethephon and 1-MCP treatments as determined by RT-qPCR. The pear *PpACTIN* gene (Accession number: JN684184) was used for normalizing the expression data. The mean values ± SD are shown from three biological replicates. BT, before treatment.

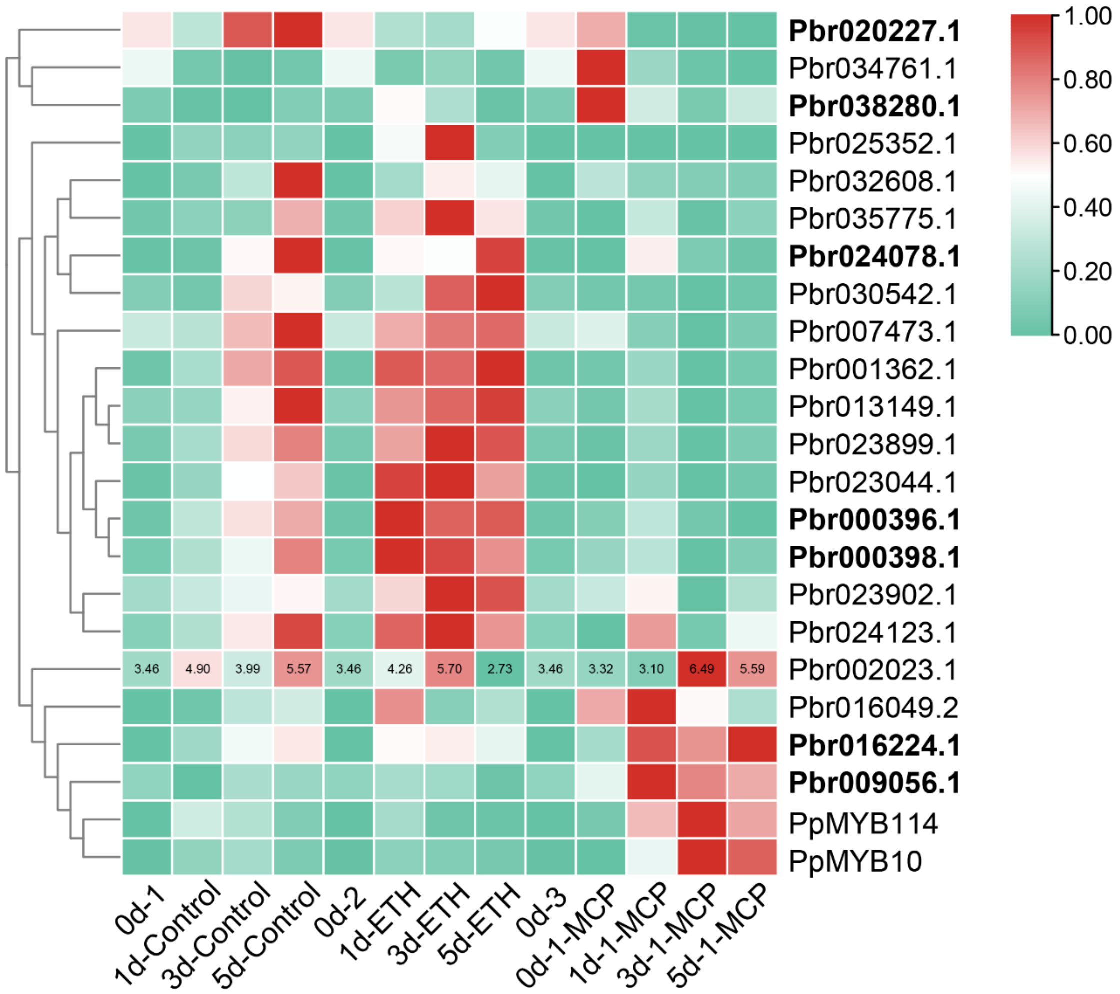

Supplemental Figure S2 (Supports Figures 1 and 3). Expression patterns of *PpMYB10*, *PpMYB114* and candidate *AP2/ERF* genes identified on the basis of RNA-seq data in 'Hongzaosu' pear fruit after ethephon and 1-MCP treatments. The heatmap was plotted by using the FPKM values normalized with row scale from zero to one. The ERF genes with bold font are predicted transcriptional repressors with EAR motifs. The number in the heatmap stands for the FPKM value of Pbr002023.1.

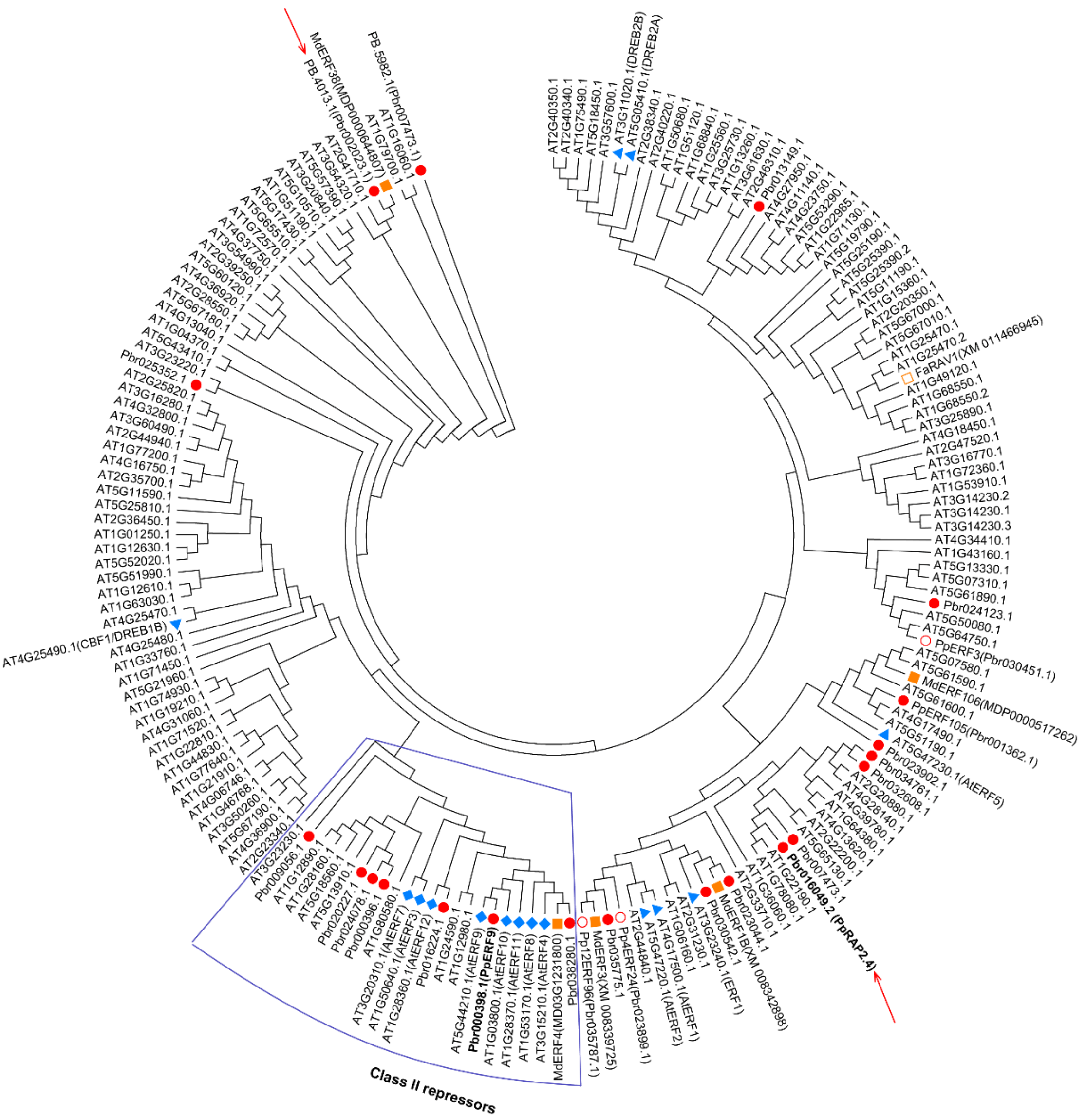

Supplemental Figure S3 (Supports Figures 1 and 3). Phylogenetic analysis of candidate ERF TFs from pear, apple, strawberry and Arabidopsis. The protein sequences from pear (Pbr or PB, the ERF TFs highlighted with red dots were identified from previous transcriptome data, the ERF TFs highlighted with red circles were reported in previous studies which regulated anthocyanin biosynthesis in pear), apple (Md, highlighted with orange square), strawberry (Fa, highlighted with orange hollow square) and Arabidopsis (AT: activators highlighted with blue triangle, repressors highlighted with blue rhombus). The phylogenetic tree was constructed using the Maximum-likelihood method of the MEGA 5.0 program.

```
Pbr000396.1-clone     ATGGGGCCGGTACGCCGCAGAAATCCGGGACCCATGGAAG     40
Pbr000396.1-genome    ATGGGGCCGGTACGCCGCAGAAATCCGGGACCCGTGGAAG     40

Pbr000396.1-clone     AAGACGAGGGTCTGGCTCGGCACATTCG............     68
Pbr000396.1-genome    AAGACGAGGGTCTGGCTCGGCACATTCGCCGCGCCTCCTG     80

Pbr000396.1-clone     .............................ACACGCCGGAG     79
Pbr000396.1-genome    CGCCGCCCGTCTCTGGCTCGGCACATTCGACACGCGGGAG    120

Pbr000396.1-clone     GAAGCCGCCCTCGCCTACGACGGCGCCGCCAGGACGCTCC    119
Pbr000396.1-genome    GAAGCCGCCCTCGCCTACGACGGCGCCGCCAGGACGCTCC    160

Pbr000396.1-clone     GCGGCGCCAAGGCCAAGACCAACTTCCCTGCGCCGATCCC    159
Pbr000396.1-genome    GCGGCGCCAAGGCCAAGACCAACTTCCCTGCGCCGATCCC    200

Pbr000396.1-clone     CAGCGGCATATCCCTCGACCTCAACGCTCCCTCCGCCGCC    199
Pbr000396.1-genome    CGGCGGCATATCCGTCGACCTCAACGCTCCCTCCGCCGCC    240

Pbr000396.1-clone     CACTGGGCCACTCACTCCGGCCGCCTCGTCGAATTCCTCC    239
Pbr000396.1-genome    CACTGGGCCACTCACTCCGGCCGCCTCGTCGAATTCCTCC    280

Pbr000396.1-clone     ACACCGGCGTCCTCAACGACATGGGCAGCGGCACTTCCAC    279
Pbr000396.1-genome    ACACCGGCGTCCTCAACGACATGGGCAGCGGCACTTCCAC    320

Pbr000396.1-clone     GAATGATACGCTGCCGTTGATGGCGAGGAGGGAACCTCAG    319
Pbr000396.1-genome    GAATGATACGCTGCCGTTGATGGCGAGGAGGGAACCTCAG    360

Pbr000396.1-clone     CAAACTCCAGTTGGTGTGGCGGGAAATGTCGGCGGCGGCG    359
Pbr000396.1-genome    CAAACTCCAGTTGGTGTGGCGGGAAATGTCGGCGGCGGCG    400

Pbr000396.1-clone     GTAATGCCACGGTCGCGGAAGGTTCGGGTTTCGGCAGTAC    399
Pbr000396.1-genome    GTAATGCCACGGTCGCGGAAGGTTCGGGTTTCGGCAGTAC    440

Pbr000396.1-clone     GGCGTCTTTTCTGGGACTGGTCCGGCGTGGCTTGCCGATT    439
Pbr000396.1-genome    GGCGTCTTTTCTGGGACTGGTCCGGCGTGGCTTGCCGATT    480

Pbr000396.1-clone     GATCTCAATGAGCCTCCTCCCTTGTGGCTGTG            471
Pbr000396.1-genome    GATCTCAATGAGCCTCCTCCCTTGTGGCTGTG            512
```

Supplemental Figure S4 (Supports Figure 1). Alignment of the cloned sequence and genomic sequence of Pbr000396.1.

A Translation of

> Pbr000396.1-clone

MGPVRRRNPGPMEEDEGLARHIRHAGGSRPRLRRRRQDAPRRQGQDQLPCADPQRHIPRP
QRSLRRPLGHSLRPPRRIPPHRRPQRHGQRHFHE*YAAVDGEEGTSANSSWCGGKCRRRR*CH
GRGRFGFRQYGVFSGTGPAWLAD*SQ*ASSLVAVRIHRARA

AP2 superfamily

>Pbr000396.1-genome

MGPVRRRNPGPVEEDEGLARHIRRASCAARLWLGTFDTREEAALAYDGAARTLRGAKAKTNF
PAPIPGGISVDLNAPSAAHWATHSGRLVEFLHTGVLNDMGSGTSTNDTLPLMARREPQQTPVG
VAGNVGGGGNATVAEGSGFGSTASFLGLVRRGLPIDLNEPPPLWL*

EAR motif

B Translation of

>PpERF9 (Pbr000398.1)

MAPREKTAAAAVRMNGNGNVKEVHFRGVRKRPWGRYAAEIRDPGKKSRVWLGTFDTAEEAAR
AYDAAAIEFRGAKAKTNFPQPSENVSLNVTKNNNNKISSGSNNQSPSQSSTVESSSREPPALMV
ESSPLDLNLAHGVSSGGFGSAPMRFPFQHHQVPGFGAVIGVPSSAPAAANQVLYFDSVFRASV
MKNRQFHPRMRFDHPNFHEPDFHAAGAAQSDSDSSSVVDLNQNDLPRGGGGFDLNLPPPPE
LA

EAR motif EAR motif

Supplemental Figure S5 (Supports Figure 1). (A) Comparison of protein sequences and domains of Pbr000396.1 in genome data and our cloned results. (B) Protein sequence and EAR motifs of PpERF9 (Pbr000398.1).

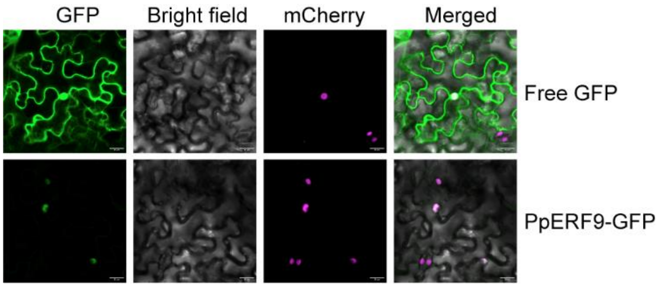

Supplemental Figure S6 (Supports Figure 1). Subcellular localization of PpERF9 in *N. benthamiana* leaf cells.

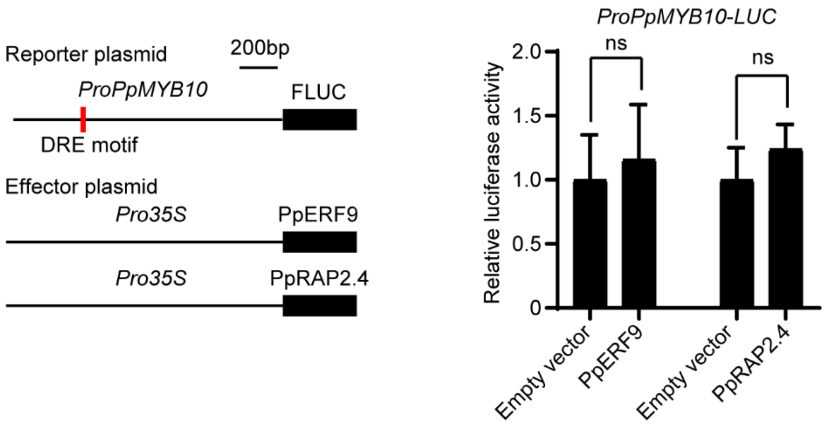

Supplemental Figure S7 (Supports Figures 1 and 3). A dual-luciferase assay demonstrated that PpERF9 and PpRAP2.4 have no effect on *PpMYB10* promoter activity. The promoter of *PpMYB10* was cloned into the pGreenII 0800-LUC (firefly luciferase) vector, and the full length CDS of *PpERF9* and *PpRAP2.4* were cloned into the pGreenII 0029 62-SK vector, respectively. The empty vector of SK was used as control. The relative luciferase activity was analyzed. Error bars represent the standard deviation of six biological replicates. The ‘ns’ indicates no significance.

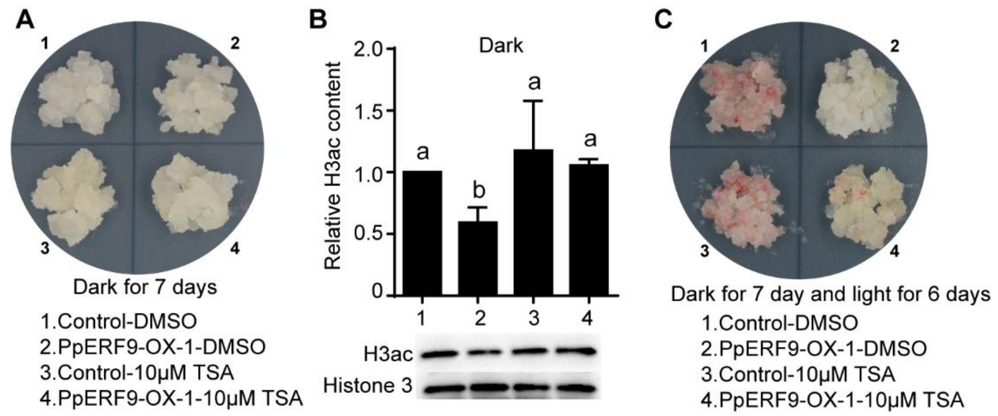

Supplemental Figure S8 (Supports Figure 6). The suppressive effect of PpERF9 on anthocyanin biosynthesis depends on histone deacetylation. (A, B) PpERF9 decreased the histone H3 acetylation (H3ac) level in pear calli. A trichostatin-A (TSA) treatment recovered the H3ac level in *PpERF9*-OX-1 pear calli. Trichostatin-A is an effective histone deacetylase inhibitor. The TSA treatment involved the addition of 10 μM TSA (dissolved in 1% DMSO) to the pear calli growth medium. A 1% DMSO solution was added to the growth medium as the negative control. Pear calli were treated for 7 days in darkness and sampled for an analysis of H3ac levels. (C) The TSA treatment partly recovered the red coloration of *PpERF9*-OX pear calli. Pear calli were treated with 10 μM TSA for 7 days and then exposed to light for 6 days. Error bars represent the standard deviation of three biological replicates. Different lowercase letters above the error bars indicate significant differences according to a one-way ANOVA with Tukey's test ($P < 0.05$).

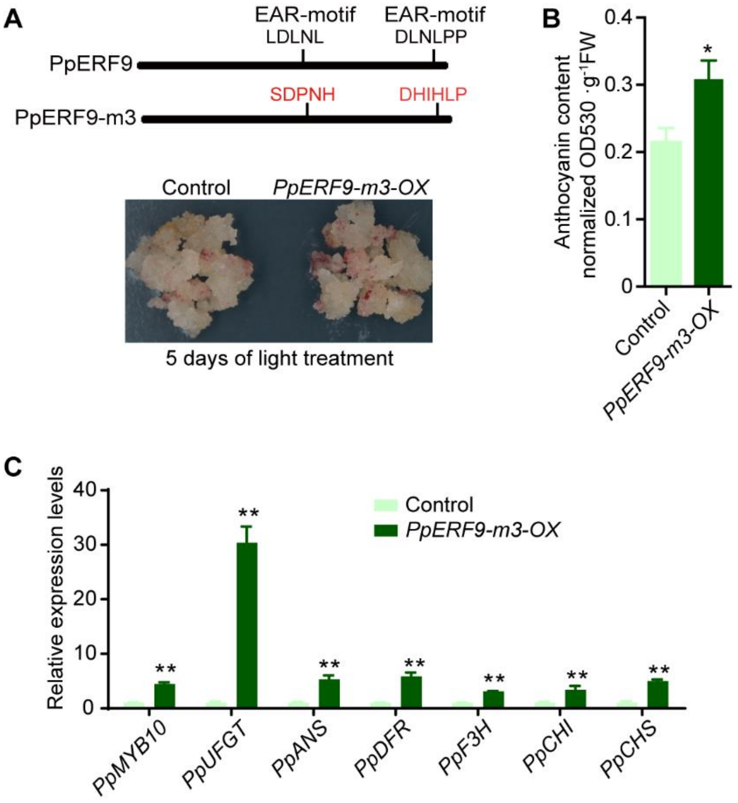

Supplemental Figure S9 (Supports Figure 6). Functional analysis of PpERF9-m3 in 'Clapp's Favorite' pear calli. (A) Overexpression of *PpERF9-m3* induced anthocyanin accumulation in pear calli. The calli transformed with the empty vector (pCAMBIA1301) were used as the negative control. Pear calli were incubated under strong light at 17 °C for 5 days. (B) Anthocyanin contents in *PpERF9-m3*-OX pear calli after the light treatment. (C) Expression levels of the anthocyanin-related genes in *PpERF9-m3*-OX pear calli after the light treatment. Error bars represent the standard deviation of three biological replicates. Asterisks indicate significantly different values (*P < 0.05 and **P < 0.01).

Supplemental Table S1. Primers for qPCR analysis in this study.

Gene	Forward primer (5'to 3')	Reverse primer (5'to 3')
PpPAL	TCTGCCAGGGAAAGATTATCG	TGAAGTTGAATGGAATGGAATGC
PpCHS	GGGTGTACTCTTCGGATTTGG	AAAGGCGGAAACAATACATATACG
PpCHI	GAACGGGTGCAAGGAATCTA	AACAGGAGTCCCTCCCAAGT
PpF3H	GGAGAAAGACAAAGTGGAGATAAAGC	ACAAGAAGTGGAAAGGCAAAGTTAC
PpDFR	ACTGAGGCTGCTGAGGAGAG	TCAAATCCAAGCTGGTAAATGT
PpANS	AGTTGTTCAGGAAAAGCCAAGAGG	ACAAAGCAGGCAGATAGGAGTAGC
PpUFGT	GGTCAAACAACTGGCAGTAGAG	TGTCCGCCCGTAGATGGTA
PpMYB10	CAGCAGAAGATTTAAGTACGCCATC	TTCTAACAAGGTCTCCCACCAATC
PpMYB114	GCCACATCCGTCATAAGACCTC	GCCACTCATGTGTAACCCTTC
PpbHLH3	TATGGTGACGGGGACTTCTC	CCACCGTGGTCTCAATTCTT
PpbHLH33	CTGATAGAGATGAGATGCCCTTACC	GGATGGATGATTGGACCGAGTG
PpRAP2.4	AGCCCAGTTTCTACGACGAC	TGCTGGTGGTGCTTCTCAAAT
PpERF9	CCGCGAATCAGGTGCTCTAT	AAATCGGGCTCGTGGAAGTT
PpERF9-m3	CAGAGCCAGCGTGATGAAGA	CGGTGGTGGAAGATGGATGT
PpActin	CCATCCAGGCTGTTCTCTC	GCAAGGTCCAGACGAAGG

Supplemental Table S2. Primers for ChIP-qPCR analysis in this study.

Gene	Forward primer (5'to 3')	Reverse primer (5'to 3')
PpMYB114-ChIP-S0	ATATTGCCGCCATTTTCTTG	TCGCATGACTTGCATATAATACAC
PpMYB114-ChIP-S1	CTTGCCGTCTTTGTCGTCTC	CTGTGGTTTTGGAGGAGAGC
PpMYB114-ChIP-S2	ACGTCGGTGGCCTTATGAT	GTGCCACTTTCCATCTCCAT
PpMYB114-ChIP-S3	GTTCCTCGCGAAACAGGTAA	ACCACCTCTGTCTGCAGCTT
PpRAP2.4-ChIP-S1	GGTGTGAACGCGTCATAAAA	GCAGACACCTGTCCTTTTCC
PpRAP2.4-ChIP-S2	GGGGGTTTGGAAAAATCAGT	AAAGAACAGGAAGCGGGTTT
PpRAP2.4-ChIP-S3	GGCTCCCTAAGAACCGTACC	AGAGGCTTGTACTCGCCAAA
PpActin	CCATCCAGGCTGTTCTCTC	GCAAGGTCCAGACGAAGG
