# Supplementary material for: The ethylene-responsive transcription factor PpERF9 represses PpRAP2.4 and PpMYB114 via histone deacetylation to inhibit anthocyanin biosynthesis in pear
Source: Plant Cell. 2023 Mar 14;35(6):2271–92. doi: 10.1093/plcell/koad077 (PMC10226596; doi:10.1093/plcell/koad077)
Supplement: koad077_Supplementary_Data [file tpc22.01128.PeerReviewReport.pdf › tpc22.01128.PeerReviewReport.pdf]

The ethylene-responsive transcription factor PpERF9 represses *PpRAP2.4* and *PpMYB114* expression via histone deacetylation to inhibit anthocyanin biosynthesis in pear

Junbei Ni, Simai Wang, Wenjie Yu, Yifei Liao, Chen Pan, Manman Zhang, Ruiyan Tao, Jia Wei, Yuhao Gao, Dongsheng Wang, Songling Bai, Yuanwen Teng

Corresponding authors: Yuanwen Teng, ywteng@zju.edu.cn, and Songling Bai, songlingbai@zju.edu.cn

Review timeline:

**TPC2021-RA-00714**	Submission received:	Aug. 18, 2021
	1st Decision:	Sept. 18, 2021 *manuscript declined*
**TPC2022-RA-01128**	Submission received:	Nov. 18, 2022
	1st Decision:	Jan. 9, 2023 *revision requested*
**TPC2022-RA-01128R1**	1st Revision received:	Jan. 24, 2023
	2nd Decision:	Feb. 6, 2023 *accept with minor revisions*
**TPC2022-RA-01128R2**	2nd Revision received:	Feb. 8, 2023
	3rd Decision:	Feb. 13, 2023 *acceptance pending, sent to science editor*
	Final acceptance:	Mar. 2, 2023

REPORT: (The report shows the major requests for revision and author responses. Minor comments for revision and miscellaneous correspondence are not included. The original format may not be reflected in this compilation, but the reviewer comments and author responses are not edited, except to correct minor typographical or spelling errors that could be a source of ambiguity.)

TPC2021-RA-00714 1st Editorial decision – *declined* Sept. 18, 2021

Thank you for choosing to send your manuscript entitled "Ethylene-responsive PpERF9 represses PpRAP2.4 and PpMYB114 expression via histone deacetylation to inhibit pear anthocyanin synthesis" for consideration at The Plant Cell. Your submission has been evaluated by members of the editorial board as well as expert reviewers in your field, and we regret to inform you that we are not able to recommend publication of this manuscript in its current form. We have not made this decision lightly. We have had input from multiple scientists, and we have solicited post-review comments as well. Our present policy is to offer streamlined decisions and to not advise on the direction of the work by requesting extensive modifications or substantial additional experiments.

That said, we would be willing to re-consider a new manuscript that fully addressed the concerns raised during this review process. If you decide to resubmit to The Plant Cell, it will be evaluated as a new submission subject to full assessment by the editorial board, and if sent for external review, a new set of reviewers might be chosen. Reviewers will be asked to assess your work as a new manuscript (i.e. are the claims fully supported by the data and do the results presented move the field forward?), and not only whether previous reviewer comments have been addressed.

The most important comments in guiding our decision included concerns regarding how some key experiments were performed and interpreted, and failing to put into context the current study with previous studies, for example, related to the role of MYB10 in the regulation of anthocyanins.

In addition to the concerns raised by the reviewers regarding experimental aspects of the study, we would like to invite you to consider the following issues:

-) Supplemental Fig 1C: The term relative intensity is not appropriate to describe the gene expression results. Also, the legend needs to indicate whether these are RT-qPCR experiments, what was used for normalization, the use of replicates, etc.

-) Line 145: The ethephon treatment induces first the expression of several of the genes mentioned to then repress them - the text in the manuscript does not reflect this. The same is true for MYB114, which is not what we understand from the text.

-) Supplemental Fig. 2C: It does not show a co-expression analysis but a heat map of the expression of a subset of genes. Details on the co-expression analysis are lacking.

-) Supplemental Fig. 3: The legend lacks details on how the phylogenetic tree was constructed

-) Figure 1F: Please include a bar to indicate the size and relative distance to each other of the fragments used in the ChIP-PCR. It is unclear if the replicates were done on three different calli (unclear in the legend and in the text on what tissue ChIP were done), or if the same chromatin was used for the ChIP-PCR.

-) Figure 1G: Include in the legend information on what SK means. What tissue was used for this? Why is the tissue expressing high levels of MYB114 before transformation?

-) Figure 2: Unclear what the error bars are in Fig. 2B - the legend should explain what the numbers mean

-) The gel shift in your figures (panel E) is sub-optimal and may need to be repeated:

The mutant probe is in lower amounts.

10x competitor gives stronger shift than no competitor.

The legend does not indicate how much recombinant protein was used.

-) Fig. 6B - you say in line 290 "ChIP-qPCR assay revealed that PpERF9 greatly increased the PCR-based detection of the S1 region of the PpRAP2.4 promoter that ERF9" - the increase is ~ 2 fold, which is far from a great increase. Once again, what is the distance between the probes used?

-) Abstract: What is called an indirect regulatory pathway is generally known as a coherent feedforward loop (https://pubmed.ncbi.nlm.nih.gov/14530388/)

-) Secondary metabolites are better known these days as specialized metabolites

It will be important to convince the editors and reviewers that the study adds significant new understanding(s) of mechanisms or processes, or otherwise represents a significant advance in the field, and that the major claims made are fully justified by the data presented. This includes careful consideration and explanation of the various controls used in experiments, the extent and manner of replication, and the statistical analyses used. Sampling methods and the nature of "biological replicates" should be described precisely (i.e. different plants, parts of plants, pooled tissue, independent pools of tissue, sampled at different times, etc.), along with a clear description of and rationale for any statistical analyses conducted. The reader should know exactly what was sampled; what forms the basis of the calculation of any means and other statistical variables and parameters reported. This is also necessary to ensure that proper statistical analysis was conducted.

-- Reviewer comments:

TPC2022-RA-01128 Submission received **Nov. 18, 2022**

Reviewer comments on previously declined manuscript and **author responses:**

<u>Editors:</u>

The most important comments in guiding our decision included concerns regarding how some key experiments were performed and interpreted, and failing to put into context the current study with previous studies, for example, related to the role of MYB10 in the regulation of anthocyanins.

Thank you for your comments and suggestions.

Firstly, according to the editors' and reviewers' comments, we have repeated all the transient overexpression and

VIGS assays in 'Hongzaosu' pear fruit this year. We modified the transient overexpression and VIGS assays to get better effects. For transient overexpression assays, we cloned the CDS of genes into pCAMBIA1301 vector with a 35S promoter and used the empty vector of pCAMBIA1301 as the negative control instead of pGreenII 0029 62-SK vector. Furthermore, we transformed these plasmids into *Agrobacterium Tumefaciens* EHA105 which owned a better infective effect instead of GV3101 (Figure 2A-C, Figure 4A-C). For the VIGS assays, we prolonged the infection time to 6 or 7 days instead of 3 days to get a better effect (Figure 2D-F, Figure 4D-F). We also used a pair of PCR primers which are located in the pTRV2 vector to check whether the vector was expressed in the pear fruit peel (Responding Fig. 3, the figures in the responding letter were named as Responding Figure here). Furthermore, a small RNA sequencing was performed to confirm whether small RNA was generated in the target gene fragment in the VIGS assay (Responding Fig. 4). The repeated results also confirmed that PpERF9 inhibited anthocyanin biosynthesis while PpRAP2.4 induced anthocyanin biosynthesis in pear fruit, which were consistent with our previous results. These results indicated that our transient overexpression and VIGS assays in pear fruit were reliable and stable. The details will be presented in the reply to editors' and reviewers' comments.

Secondly, according to the editors' and reviewers' suggestion, we further analyzed the promoter of *PpMYB10*, and found an ERF-binding site (DRE motif) in that (Supplemental Figure S7). We performed the dual-luciferase assay to analyze the potential regulatory pattern of PpRAP2.4 and PpERF9 on *PpMYB10*. The results showed that both PpRAP2.4 and PpERF9 had no effect on the *PpMYB10* promoter activity (Supplemental Figure S7). So, we think that *PpMYB10* might be regulated by ethylene signaling in another pathway. We added these results in lines 195-199, 225- 227 and 285-288 of revised manuscript. Furthermore, considering that reviewer 2 mentioned whether PpMYB114 transcriptionally regulates *PpMYB10*, we also further performed a dual-luciferase assay to analyze whether PpMYB114 can activate *PpMYB10* promoter. The result showed that PpMYB114 promoted the activity of *PpMYB10* promoter in tobacco leaves (Responding Fig. 1), indicating that PpMYB114 might transcriptionally regulate *PpMYB10* expression. This finding provides us a really good idea for further studies. However, in this study, we mainly focused on the regulation of ethylene-responsive ERF on *MYB* genes. In the future, we will do a large amount of experiments including molecular biological verification and genetic verification of the transcriptional regulation of PpMYB114 on *PpMYB10*.

Thirdly, we also revised the manuscript based on new results of additional experiments suggested by the editors and reviewers. The details will be presented in the replies to editors' and reviewers' comments.

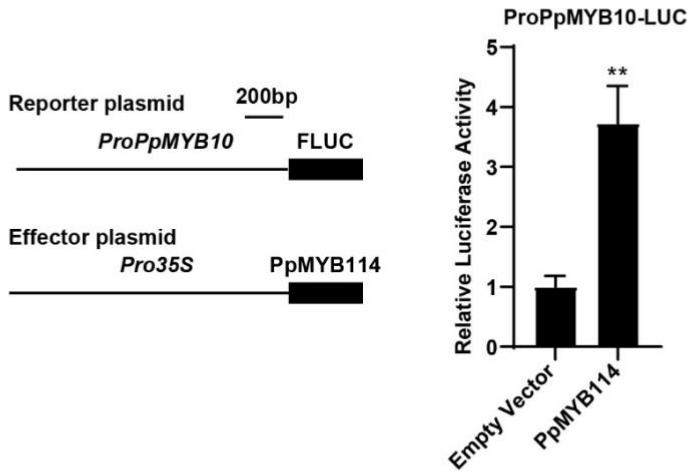

Responding Fig. 1 A dual-luciferase assay demonstrated that PpMYB114 activates *PpMYB10* promoter activity. Error bars represent the standard deviation of six biological replicates. The 'ns' indicates no significance. The promoter of *PpMYB10* was cloned into the pGreenII 0800-LUC (firefly luciferase) vector, and the full length CDS of *PpMYB114* was cloned into the pGreenII 0029 62-SK vector, respectively. The empty vector of SK was used as control. The relative luciferase activity was analyzed.

Supplemental Fig 1C: The term relative intensity is not appropriate to describe the gene expression results. Also, the legend needs to indicate whether these are RT-qPCR experiments, what was used for normalization, the use of replicates, etc.

Thank you for your suggestion. We have changed the "relative intensity" into "relative expression level" to describe the gene expression results. Furthermore, these are results of RT-qPCR experiments and *PpACTIN* was used for normalization. Three biological replicates were used in this experiment. We have added this information into the figure legends.

Line 145: The ethephon treatment induces first the expression of several of the genes mentioned to then repress them - the text in the manuscript does not reflect this. The same is true for MYB114, which is not what we understand from the text

Thank you for your comments. In our manuscript, we mainly describe comparison of the expression levels of genes between treatments and control. The expression level of *PpMYB114* was induced 1 day after ethylene treatment, but actually ethylene inhibited its expression compared with control at the same time. But the expression levels of several structural genes were firstly induced and then inhibited by ethylene treatment compared with control. Thus, we have changed the description in lines 178-188 to make it more accurate. The description is as follows: "The expression levels of anthocyanin biosynthetic structural genes (*PpCHI*, *PpCHS*, *PpF3H*, *PpDFR*, *PpANS*, and *PpUFGT*) were significantly upregulated 3 days after a 1-methylcyclopropene (1-MCP) treatment, while the expression levels of *PpANS* and *PpUFGT* were repressed 3 days after an ethephon treatment compared with control (Supplemental Figure S1C). Furthermore, an ethephon treatment firstly slightly induced and then repressed the expression levels of *PpCHI*, *PpCHS* and *PpF3H* compared with control. Notably, expression levels of *PpMYB10* and *PpMYB114* increased more than 400-fold and 30-fold after the 1-MCP treatment (relative to the control level), respectively, whereas their expression levels were repressed significantly following the ethephon treatment compared with control (Supplemental Figure S1D)."

Supplemental Fig. 2C: It does not show a co-expression analysis but a heat map of the expression of a subset of genes. Details on the co-expression analysis are lacking.

Thank you for your comments. In Supplemental Figure S2, we showed the expression pattern of a subset of genes. We have reported the co-expression analysis results in our previous study (Ni et al. 2020) (Responding Fig. 2). In that paper, we found that 21 ERF TFs were positively or negatively correlated with the expression of *PpMYB114* and *PpMYB10*. Thus, we didn't show the co-expression analysis in this paper. To make it clearer, we changed the sentences as shown in lines 199-210: "From previously identified 21 ERF TFs (Ni et al. 2020), which were positively or negatively correlated with anthocyanin content as well as *PpMYB10* and *PpMYB114* expression, we further screened two ERF TFs (Pbr000396.1 and Pbr000398.1) which positively responded to ethylene signaling and were clustered into class II with EAR-motif (Supplemental Figure 2 and 3). Then we cloned the CDS of these two ERF genes. However, we found that Pbr000396.1 had a 41-bp deletion compared with the genome sequence (Supplemental Figure S4), which led to a frameshift mutation as well as the missing of AP2 superfamily domain and EAR motif (Supplemental Figure S5). In addition, there are two EAR motifs in PpERF9, one of which (DLNxxP-type) is located in the C- terminal region, whereas the other (LxLxL-type) is located in the middle part of the protein (Figure 1B), implying that PpERF9 is a transcriptional repressor." Furthermore, we added PpMYB114 and PpMYB10 in the heatmap in Supplemental Figure S2.

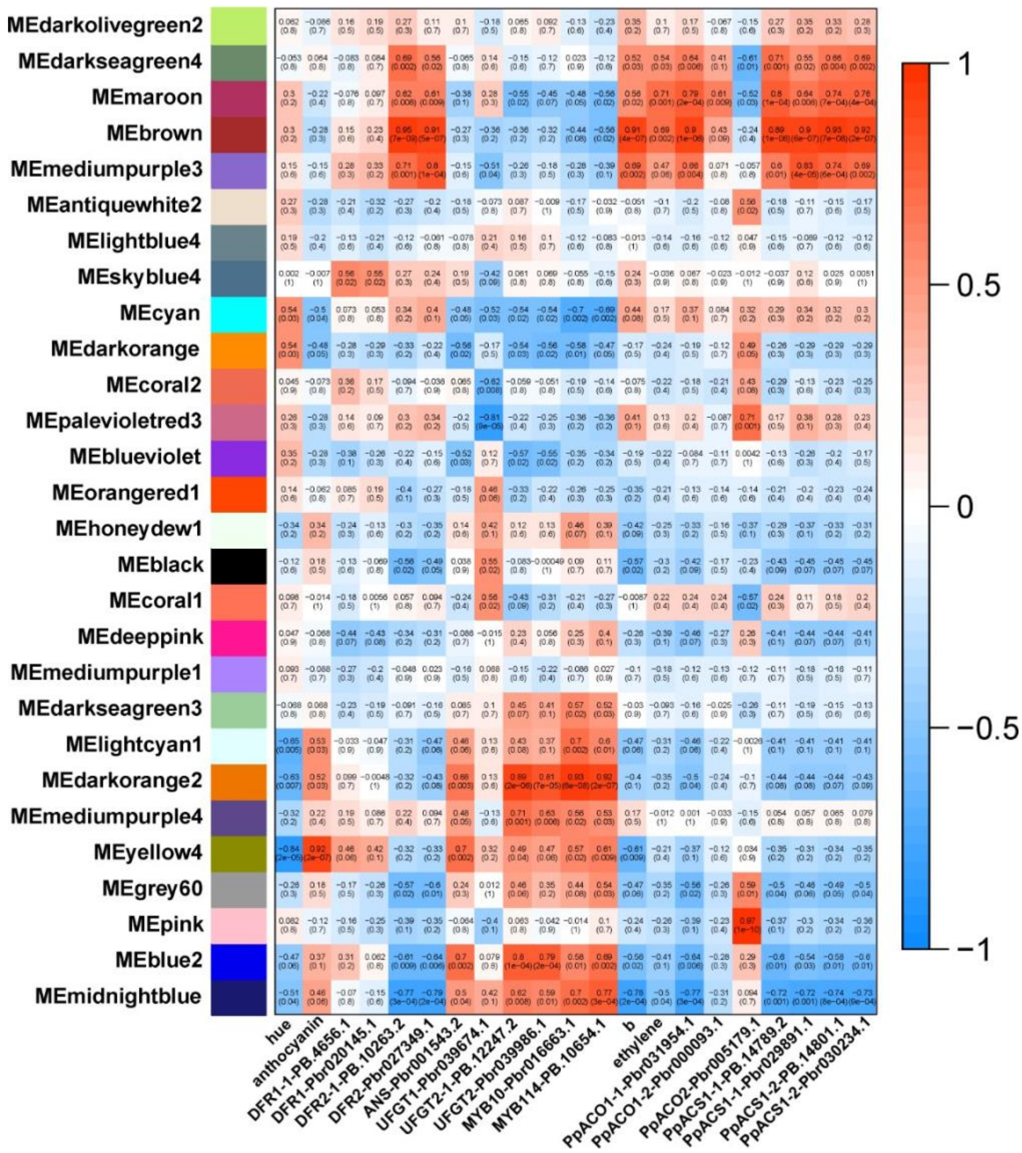

Responding Fig. 2. Identification of transcripts by the weighted gene co-expression network analysis (WGCNA) of hormone-treated 'Hongzaosu' pear fruit peels. Module– trait correlations and the corresponding *P*-values are in parentheses. The left panel presents the 28 modules. The color scale on the right presents the module–trait correlations from −1 (blue) to 1 (red). The panels "hue", "anthocyanin", "b", and "ethylene" represent the hue angles, anthocyanin contents, *b values, and ethylene-release rates, respectively. The other panels represent the changes in gene expression levels (Ni et al. 2020).**

References:

Ni J, Zhao Y, Tao R, Yin L, Gao L, Strid Å, Qian M, Li J, Li Y, Shen J, Teng Y, Bai S (2020) Ethylene mediates the branching of the jasmonate-induced flavonoid biosynthesis pathway by suppressing anthocyanin biosynthesis in red Chinese pear fruits. Plant Biotechnology Journal 18 (5):1223-1240

Supplemental Fig. 3: The legend lacks details on how the phylogenetic tree was constructed

Thank you for your suggestions and comments. Based on the second reviewer's and your suggestions. We added the ERF TFs which positively or negatively regulate anthocyanin biosynthesis or respond to ethylene signaling mentioned in introduction and discussion in the phylogenetic tree. And we also added the details in the figure

legend as following: "Phylogenetic analysis of candidate ERF TFs from pear, apple, strawberry and Arabidopsis. The protein sequences from pear (Pbr or PB, the ERF TFs highlighted with red dots were identified from previous transcriptome data, the ERF TFs highlighted with red circles were reported in previous studies which regulated anthocyanin biosynthesis in pear), apple (Md, highlighted with orange square), strawberry (Fa, highlighted with orange hollow square) and Arabidopsis (AT: activators highlighted with blue triangle, repressors highlighted with blue rhombus). The phylogenetic tree was constructed using the Maximum-likelihood method of the MEGA 5.0 program."

Figure 1F: Please include a bar to indicate the size and relative distance to each other of the fragments used in the ChIP-PCR. It is unclear if the replicates were done on three different calli (unclear in the legend and in the text on what tissue ChIP were done), or if the same chromatin was used for the ChIP-PCR.

Thank you for your suggestion. We have added a bar in Fig. 1F to indicate the size and relative distance of the fragments used in the ChIP-PCR. For the ChIP-PCR assay in Fig. 1F, we used the *PpERF9*-MYC overexpressing pear calli to analyze the binding of PpERF9 to *PpMYB114* promoter. Pear calli overexpressing the MYC protein were used as a negative control. Cross-linked chromatin samples were extracted from PpERF9- MYC-overexpressing pear calli and pear calli as negative control with three biological replicates and precipitated with an anti-MYC antibody. We added the detailed information in the figure legend to make it clear.

Figure 1G: Include in the legend information on what SK means. What tissue was used for this? Why is the tissue expressing high levels of MYB114 before transformation?

SK means the empty vector of pGreenII 0029 62-SK. We have added the detailed information in the figure legend. We used tobacco (*Nicotiana benthamiana*) leaves to perform the dual luciferase assay. In this assay, we used the promoter of *PpMYB114* to ligase with the LUC reporter gene. The *PpMYB114* promoter could be activated by light signaling. In this experiment, we put the tobacco under white light condition, so we think that the *PpMYB114* promoter might be regulated by some internal factors in light signaling pathway in tobacco. We added the schematic diagram of the vectors in Figure 1G to make it easier to be understood.

Figure 2: Unclear what the error bars are in Fig. 2B - the legend should explain what the numbers mean

Thank you for your suggestion. The error bars in Figure 2B represent the standard deviation of three biological replicates. In Figure 2A-F, we repeated all the transient overexpression and silencing experiment, so we modified the figure legend.

The gel shift in your figures (Fig. 3 panel E) is sub-optimal and may need to be repeated: The mutant probe is in lower amounts.10x competitor gives stronger shift than no competitor. The legend does not indicate how much recombinant protein was used.

Thank you for your suggestion. We have repeated the EMSA. Furthermore, we used 3 µg PpRAP2.4-His protein. We have added this information in the figure legend.

Fig. 6B - you say in line 290 "ChIP-qPCR assay revealed that PpERF9 greatly increased the PCR-based detection of the S1 region of the PpRAP2.4 promoter that ERF9" - the increase is ~ 2 fold, which is far from a great increase. Once again, what is the distance between the probes used?

Thank you for your suggestion. We have modified the sentence as follows to make it more appropriate: "A ChIP-qPCR assay revealed that PpERF9 significantly increased the PCR-based detection of the S1 region of the *PpRAP2.4* promoter containing the DRE motif" in line 345. Furthermore, we added a bar in Figure 6B and Figure 6D to indicate the size and relative distance of the fragments used in the ChIP-PCR. Furthermore, we also add bars to indicate the distances in Figure 8 and 9.

Abstract: What is called an indirect regulatory pathway is generally known as a coherent feedforward loop (https://pubmed.ncbi.nlm.nih.gov/14530388/)

Thank you for your suggestion. We deleted the "indirect" in the abstract.

Secondary metabolites are better known these days as specialized metabolites.

Thank you for your suggestion. We changed "secondary metabolites" into "specialized metabolites" in the introduction in lines 92.

It will be important to convince the editors and reviewers that the study adds significant new understanding(s) of mechanisms or processes, or otherwise represents a significant advance in the field, and that the major claims made are fully justified by the data presented. This includes careful consideration and explanation of the various controls used in experiments, the extent and manner of replication, and the statistical analyses used. Sampling methods and the nature of "biological replicates" should be described precisely (i.e. different plants, parts of plants, pooled tissue, independent pools of tissue, sampled at different times, etc.), along with a clear description of and rationale for any statistical analyses conducted. The reader should know exactly what was sampled; what forms the basis of the calculation of any means and other statistical variables and parameters reported. This is also necessary to ensure that proper statistical analysis was conducted. The Plant Cell now requires authors to complete and submit an author revisions checklist upon submission of a revised manuscript. The aim of the checklist is to aid authors in preparing a high quality manuscript, facilitate the review and assessment of revised manuscripts, and help to ensure that journal standards are maintained across the board. If your manuscript is accepted, the completed checklist will be published as supplemental material attached to the article online. Please download a copy of the checklist (a fillable PDF form) at this link, for submission with your revised manuscript: https://tpc.msubmit.net/html/Author_Revisions_Checklist.pdf.

Note also that supplemental materials should be restricted to large datasets and tables, presentation of replicates, and validation of reagents, methods, or genotypes. Any data that are used to support the main claims must be in the main manuscript. Supplemental figure legends must indicate what figure in the main manuscript is supported by the supplemental data presented.

Thank you for your comments. We have checked our manuscript and revised it according to your comments and reviewers' suggestions. We also submitted the revisions checklist upon submission of our revised manuscript.

Reviewer #1:

My opinion and overall rating of this manuscript is primarily based on following points: (1) Importance of the findings and scientific merit (2) Quality of experiments, and (3) Clarity of presentation. Regulation of anthocyanin biosynthesis has been widely studied and regulatory genes involved in this process have been identified in numerous plant species, including pear. Additionally, phytohormones such as jasmonic acid (JA) and ethylene (ET) are known regulators anthocyanin. However, molecular mechanism of ET-mediated regulation anthocyanin biosynthesis is not well studied. The authors identified an ET-responsive ERF (ERF9) that directly represses the expression of two regulatory genes (MYB114 and RAP2.4) and inhibits anthocyanin biosynthesis in pear. Overexpression and VIGS of these regulatory genes in pear fruits or callus have complementary effects on gene expression and anthocyanin biosynthesis. More importantly, ERF9 interacts with TPL-like protein to affect the histone acetylation status in the promoter regions of MYB114 and RAP2.4. This manuscript provides significant new information on the molecular mechanism of ET-mediated regulation of anthocyanin biosynthesis in an important fruit crop. Although the results support the claim made in this manuscript, I am wondering about the soundness of some of the key results, especially VIGS and overexpression in callus or fruits. The authors should repeat some of these experiments and provide reasonable explanation to the queries below to support their claims.

Thank you for your comments. We have repeated all the transient overexpression and VIGS assays in 'Hongzaosu' pear fruit this year. We modified the transient overexpression and VIGS assays to get better effects. For transient overexpression assays, we cloned the CDS of genes into pCAMBIA1301 vector with a 35S promoter and used the empty vector of pCAMBIA1301 as the negative control instead of pGreenII 0029 62-SK vector. Furthermore, we transformed these plasmids into Agrobacterium Tumefaciens EHA105 which owned a better infective effect instead of GV3101 (Figure 2A-C, Figure 4A-C). For the VIGS assays, we prolonged the infection time to 6 or 7 days instead of 3 days to get a better effect (Figure 2D-F, Figure 4D-F). We also used a pair of PCR primers which are located in the pTRV2 vector to check whether the vector was expressed in the pear fruit peel (Responding Fig. 3). Furthermore, a small RNA sequencing was performed to confirm whether small RNA was generated in the target gene fragment in the VIGS assay (Responding Fig. 4). The repeated results also confirmed that PpERF9 inhibited anthocyanin biosynthesis while PpRAP2.4 induced anthocyanin biosynthesis in pear fruit, which were consistent with our previous results. These results indicated that our transient overexpression and VIGS assays in pear fruit were reliable and stable. The details will be presented in the subsequent replies.

Supp. Figure 1C and line 145: ETH significantly induces most of the pathway genes at an earlier time point which is not discussed in the manuscript.

Thank you for your comments. In our manuscript, we mainly describe comparison of the expression levels of genes between treatment and control. The expression level of *PpMYB114* was induced 1 day after ethylene treatment, but actually ethylene inhibited its expression compared with control at the same time. But the expression levels of several structural genes were firstly induced and then inhibited by ethylene treatment compared with control. Thus, we have changed the description in lines 178-188 to make it more accurate. The description is as follows: "The expression levels of anthocyanin biosynthetic structural genes (*PpCHI*, *PpCHS*, *PpF3H*, *PpDFR*, *PpANS*, and *PpUFGT*) were significantly upregulated 3 days after a 1-methylcyclopropene (1-MCP) treatment, while the expression levels of *PpANS* and *PpUFGT* were repressed 3 days after an ethephon treatment compared with control (Supplemental Figure S1C). Furthermore, an ethephon treatment firstly slightly induced and then repressed the expression levels of *PpCHI*, *PpCHS* and *PpF3H* compared with control. Notably, expression levels of *PpMYB10* and *PpMYB114* increased more than 400-fold and 30-fold after the 1-MCP treatment (relative to the control level), respectively, whereas their expression levels were repressed significantly following the ethephon treatment compared with control (Supplemental Figure S1D)."

Line146-150: Expression of MYB10 was induced more than 400-fold in response to MCP treatment, why not MYB10, as it also regulates anthocyanin biosynthesis in pear?

Thank you for your comments. We have changed the description in lines 184-189 to make it more accurate: "Notably, expression levels of *PpMYB10* and *PpMYB114* increased more than 400-fold and 30-fold after the 1-MCP treatment (relative to the control level), whereas their expression levels were repressed significantly following the ethephon treatment compared with control (Supplemental Figure S1D). These results indicate that ethylene inhibits red pear anthocyanin biosynthesis by regulating *PpMYB10* and *PpMYB114* transcription."

According to the editors' and reviewers' suggestions, we further analyzed the promoter of *PpMYB10*, and found an ERF-binding site (DRE motif). We firstly performed the dual-luciferase assay to analyze the potential regulatory pattern of PpRAP2.4 and PpERF9 on *PpMYB10*. The results showed that both PpRAP2.4 and PpERF9 had no effect on the *PpMYB10* promoter activity (Supplemental Figure S7). So, we think that *PpMYB10* might be regulated by ethylene via other pathway. We will further analyze the potential regulatory pathway of how ethylene inhibits *PpMYB10* expression in the future. We added these results in lines 195-199, 225-227 and 285-288 of revised manuscript.

Line 160-161: A co-expression network analysis revealed the negative relationships between the PpERF9 and PpMYB114 expression patterns:

I didn't find MYB114 in the co-expression heatmap.

Why was MYB10 not included in the analysis?

Thank you for your comments. In supplemental Figure S2, we showed the expression pattern of a subset of genes. We have reported the co-expression analysis results in our previous study (Ni et al. 2020) (Responding Fig. 2). In that paper, we found that 21 ERF TFs were positively or negatively correlated with the expression of *PpMYB114* and *PpMYB10*. Thus, we didn't show the co-expression analysis in this paper. To make it clearer, we changed the sentence as shown in lines 199-210: "From previously identified 21 ERF TFs (Ni et al. 2020), which were positively or negatively correlated with anthocyanin content as well as *PpMYB10* and *PpMYB114* expression, we further screened two ERF TFs (Pbr000396.1 and Pbr000398.1) which positively responded to ethylene signaling and were clustered into class II with EAR-motif (Supplemental Figure 2 and 3). Then we cloned the CDS of these two ERF genes. However, we found that Pbr000396.1 had a 41-bp deletion compared with the genome sequence (Supplemental Figure 4), which led to a frameshift mutation as well as the missing of AP2 superfamily domain and EAR motif (Supplemental Figure S5). In addition, there are two EAR motifs in PpERF9, one of which (DLNxxP-type) is located in the C- terminal region, whereas the other (LxLxL-type) is located in the middle part of the protein (Figure 1B), implying that PpERF9 is a transcriptional repressor." Furthermore, we added PpMYB114 and PpMYB10 in the heatmap in Supplemental Figure S2.

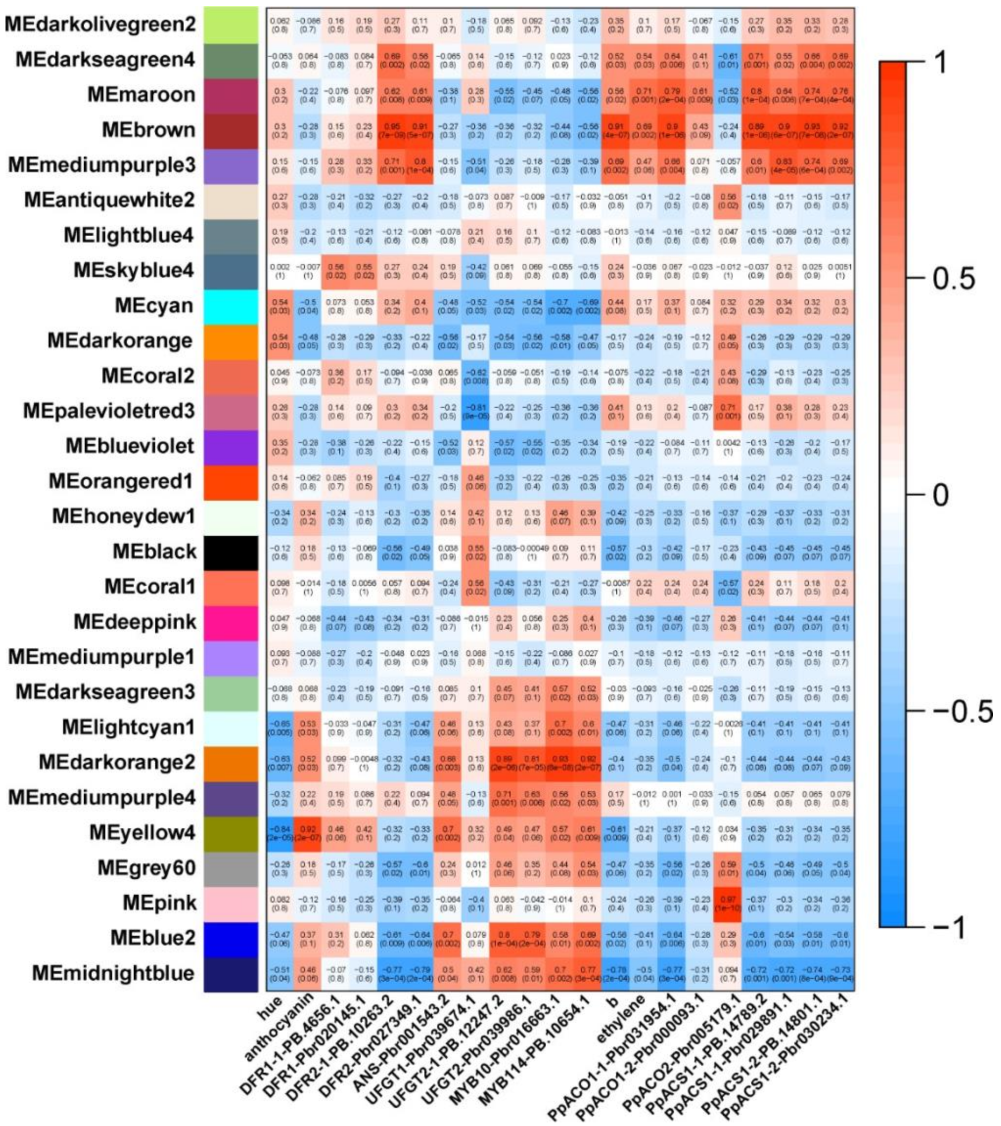

Responding Fig. 2. Identification of transcripts by the weighted gene co-expression network analysis (WGCNA) of hormone-treated 'Hongzaosu' pear fruit peels. Module– trait correlations and the corresponding *P*-values are in parentheses. The left panel presents the 28 modules. The color scale on the right presents the module–trait correlations from −1 (blue) to 1 (red). The panels "hue", "anthocyanin", "b", and "ethylene" represent the hue angles, anthocyanin contents, *b values, and ethylene-release rates, respectively. The other panels represent the changes in gene expression levels (Ni et al. 2020).**

References: Ni J, Zhao Y, Tao R, Yin L, Gao L, Strid Å, Qian M, Li J, Li Y, Shen J, Teng Y, Bai S (2020) Ethylene mediates the branching of the jasmonate-induced flavonoid biosynthesis pathway by suppressing anthocyanin biosynthesis in red Chinese pear fruits. Plant Biotechnology Journal 18 (5):1223-1240

Line 192-194 and Figure 2B, 2C: It is interesting to see that ERF9 overexpression significantly reduces anthocyanin accumulation; however, it has no effect on two key late anthocyanin pathway genes (DFR and ANS). UFGT expression is reduced by approx. 20%, whereas MYB114 expression reduced significantly.

Thank you for your comments. We have repeated the transient overexpression of *PpERF9* in 'Hongzaosu' pear fruit to analyze the result again. We modified the transient overexpression assay to get a better effect as follows. We cloned *PpERF9* into pCAMBIA1301 vector with a 35S promoter and used the empty vector of pCAMBIA1301 as the

negative control instead of pGreenII 0029 62-SK vector. Furthermore, we transformed the plasmid into *Agrobacterium Tumefaciens* EHA105 which owned a better infective effect instead of GV3101. After injection, the pear fruit were placed in dark for 1 day and then treated with strong light for 6 days to promote the accumulation of anthocyanin in the 'Hongzaosu' pear fruit peel. The results showed that the red coloration and anthocyanin content were all repressed in *PpERF9-OX* pear fruit peel compared with the fruit peel injected with empty vector (Figure 2A and B). The expression of *PpERF9* was significantly induced in *PpERF9-OX* pear fruit peel compared with control (Figure 2C). Furthermore, the expression levels of *PpRAP2.4*, *PpMYB114*, *PpMYB10*, *PpUFGT*, *PpANS*, *PpDFR*, *PpF3H*, *PpCHI*, *PpCHS* and PpPAL were all significantly down-regulated in PpERF9-OX pear fruit peel compared with control (Figure 2C). Due to the modification of the transient overexpression assay, the results showed a little bit difference with the previous study. But the phenotype in pear fruit and the overall expression trends of the anthocyanin biosynthesis-related genes were consistent with the previous results. Thus, we think that our transient overexpression is relatively stable and our results are convincing. Based on the repeated results, we updated the description in results in lines 237-239.

Line 197-198: silencing of PpERF9 significantly induced the expression of PpMYB114, PpMYB10, PpUFGT, PpANS, PpDFR, PpF3H, PpCHI, and PpCHS.

Figure 2F - VIGS of ERF9 in pear fruit: Expression of ERF9 appears to be only 15-20% less than that of the control. I am wondering about the soundness of this data and the tiny error bars? Really surprised to see a nominal change in ERF9 expression has significant effect on pathway genes.

Thank you for your comments. Actually, the expression of PpERF9 in the *PpERF9- TRV* fruit sample was 30% less than the control in the previous result. To get a more convincing results, we have repeated the transient silencing of *PpERF9* in 'Hongzaosu' pear fruit again with some modifications. We prolonged the infection time to 6 days instead of 3 days to get a better effect. Furthermore, we performed the PpERF9- silencing and control injection in the same pear fruit in the previous experiment, while we performed the PpERF9-silencing and control injection in different pear fruit in the repeated experiment. As a result, the average relative expression level of *PpERF9* was approximately 0.599 compared with control. The expression levels of *PpMYB114*, *PpMYB10*, *PpUFGT* and *PpANS* were all up-regulated in *PpERF9*-silencing fruit pear peel (Figure 2D-F). To confirm whether the pTRV vector in VIGS assay expressed in the fruit peel, a pair of PCR primers which are located in the pTRV2 vector were used to run the PCR using fruit peel cDNA (Responding Fig. 3A). The fruit peel without any injection was used as negative control. The PCR results showed that the fragment of pTRV2 was not expressed in the fruit peel without any injection, while the fragment of pTRV2 was expressed in the fruit peel which was injected with empty vector as well as PpERF9-TRV vector (Responding Fig. 3B). These results indicated that the vector of VIGS assay was indeed expressed in the transient silencing fruit peel. In addition, to further confirm whether our VIGS assay generated the corresponding small RNA in the target silencing fragment of *PpERF9* gene, we performed a small RNA sequencing to compare the number of generated small RNA mapped to the target silencing fragment in the *PpERF9-TRV* and the control pear fruit peel samples. The result showed that much more corresponding small RNA was generated in *PpERF9-TRV* pear fruit peel sample than that in control pear fruit peel sample, which further indicated that our VIGS assay was successful and convincing (Responding Fig. 4). Based on the repeated results, we updated the description in results in lines 241-243. In addition, we referred to some papers published in The Plant Cell in recent years and found the transient silencing of genes in apple fruit (Li et al., 2017; Ma et al., 2021; Wang et al., 2022) also showed approximately 0.5 to 0.7 down-regulated changes, which are similar to our results.

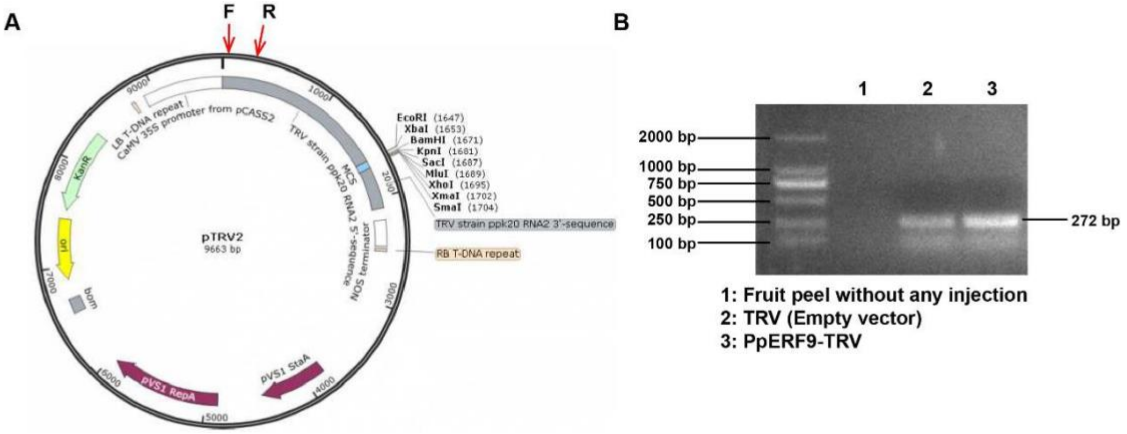

Responding Fig. 3. (A)The schematic diagram of the primers, which are located in the pTRV2 vector, used to verify the expression of the pTRV2 vector; (B) PCR results showed that the fragment of pTRV2 was expressed in the fruit peel which was injected with empty vector as well as PpERF9-TRV, while pTRV2 was not expressed in the fruit peel without any injection. The cDNA of the corresponding fruit peel was used as cloning template in this assay.

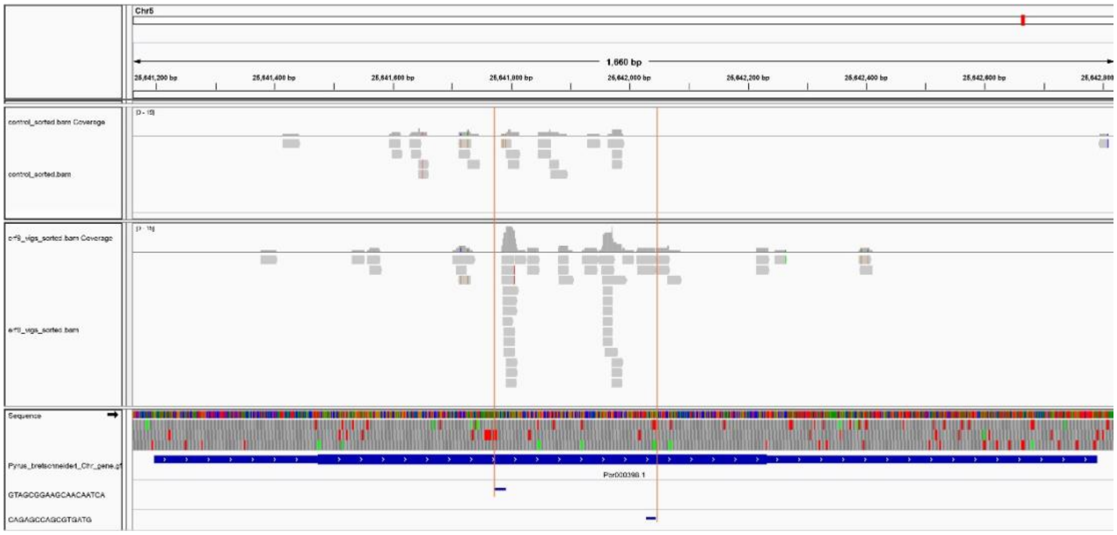

Responding Fig. 4. The generated small RNA mapped to the target fragment of *PpERF9* gene in *PpERF9-TRV* and control pear fruit peel samples. The orange lines indicate the initial and terminal points of the silencing fragment of *PpERF9*.

References:

Li T, Xu Y, Zhang L, Ji Y, Tan D, Yuan H, Wang A (2017) The jasmonate-activated transcription factor MdMYC2 regulates ETHYLENE RESPONSE FACTOR and ethylene biosynthetic genes to promote ethylene biosynthesis during apple fruit ripening. The Plant Cell 29:1316-1334

Ma H, Yang T, Li Y, Zhang J, Wu T, Song T, Yao Y, Tian J (2021) The long noncoding RNA MdLNC499 bridges MdWRKY1 and MdERF109 function to regulate early-stage light-induced anthocyanin accumulation in apple fruit. The Plant Cell 33:3309-3330

Wang S, Wang T, Li Q, Xu C, Tian J, Wang Y, Zhang X, Xu X, Han Z, Wu T (2022) Phosphorylation of MdERF17 by MdMPK4 promotes apple fruit peel degreening during light/dark transitions. The Plant Cell 34:1980-2000

Figure 2I - ERF9-overexpressing callus: Although the phenotypes of the control and OE calli are distinct, level of overexpression is less than two-fold (these are stably transformed callus). Such a marginal overexpression has significant effect on pathway gene expression. The fruit and callus overexpression data are inconsistent, leading to me to question the soundness of the experiment. For instance, expression of DFR and ANS was not affected in fruit (Figure 2C) but was significantly down in callus with marginal overexpression of ERF9 (Figure 2I). Why was CHS not included in Fig. 2I (down in ERF9-OE fruits, Figure 2C)?

Thank you for your comments. We are sorry for the confusion. Firstly, we used a pair of qPCR primer of which the forward primer is located in the CDS of *PpERF9* gene and the reverse primer is located in the pCAMBIA1301 vector to check whether the *35S::PpERF9* vector was inserted into the genome and overexpressed in the pear calli. The results showed that the CT number of qPCR was 'NA' in control pear calli sample, while the CT number was about 21 in *PpERF9-OX* pear calli, indicating that 3*5S::PpERF9* was indeed inserted into the genome and overexpressed in the pear calli (Responding Fig. 5A). Secondly, for the *PpERF9*-overespressing pear calli, we showed the expression level of *PpERF9* after 5 days of light treatment. Actually, we also checked the expression level of *PpERF9* in the *PpERF9-OX* pear calli in the dark condition. The overexpression level is about 9 folds (Responding Fig. 5B). We think that probably because light treatment induced the expression of *PpERF9* in the control pear calli, but the overexpression of *PpERF9* by 35S promoter was invariable after light treatment. Thus, the relative overexpression level of *PpERF9* decreased after light treatment compared with control. We then compared the expression level of *PpERF9* in control and *PpERF9-OX* pear calli under both dark and light conditions. The results showed that a 5-day light treatment indeed up-regulated the expression of *PpERF9* in control pear calli (Responding Fig. 5B). But the *PpERF9* expression level in *PpERF9- OX* pear calli showed no significant difference in both dark and light-treated condition (Responding Fig. 5B). To make the results clearer, we added the expression of *PpERF9* in control and *PpERF9-OX* pear calli in dark condition in Figure 2I. Furthermore, we also added the expression of *PpCHS* in Figure 2I.

Furthermore, we have repeated the transient overexpressing assay of *PpERF9* in 'Hongzaosu' pear fruit again to analyze the results. We modified the transient overexpression assay to get a better effect. We cloned *PpERF9* into pCAMBIA1301 vector with a 35S promoter and used the empty vector of pCAMBIA1301 as the negative control instead of pGreenII 0029 62-SK vector. Furthermore, we transformed these plasmids into *Agrobacterium Tumefaciens* EHA105 which owned a better infective effect instead of GV3101. After injection, the pear fruit were placed in dark for 1 day and then treated with strong light for 6 days to promote the accumulation of anthocyanin in the 'Hongzaosu' pear fruit peel. The expression of *PpERF9* was significantly induced in *PpERF9-OX* pear fruit peel compared with control (Figure 2C). Furthermore, the expression levels of *PpRAP2.4*, *PpMYB114*, *PpMYB10*, *PpUFGT*, *PpANS*, *PpDFR*, *PpF3H*, *PpCHI*, *PpCHS* and *PpPAL* were all significantly down- regulated in *PpERF9-OX* pear fruit peel compared with control (Figure 2C). Due to the modification of the transient overexpression assay, the results showed a little bit difference with the previous results. But the phenotype in pear fruit and the overall expression trends of the anthocyanin biosynthesis-related genes were consistent with the previous results. Thus, we think that our transient overexpression is relatively stable and our results are convincing. In the repeated results, we found that the expression levels of *PpANS* and *PpDFR* were all down-regulated in PpERF9-oeverepressing pear fruit peel, which is consistent with that in pear calli (Figure 2C and I).

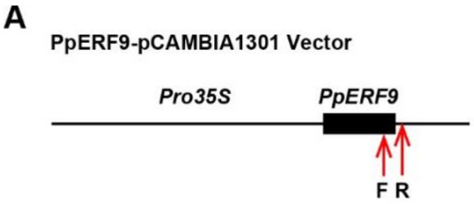

	PpActin	PpERF9
Control	18.7	NA
	18.69	NA
	18.58	NA
PpERF9-OX	18.91	20.91
	18.47	20.71
	19.2	21.19

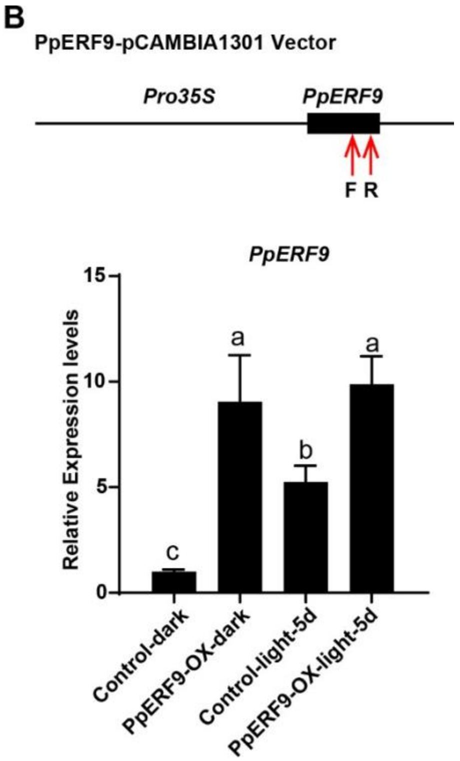

Responding Fig. 5. (A)The schematic diagram of the primers used and the CT number of *PpERF9* and *PpActin* genes in pear calli; (B) The schematic diagram of the primers used and the expression level of *PpERF9* in pear calli under dark and light treatment condition.

Line 220-221: First, because MYB114 is not included in the heatmap, it is not possible to see the expression correlation. Second, expression of 002023 is too low to see any change in response to ET or MCP.

Thank you for your suggestions. Firstly, we have added *PpMYB114* and *PpMYB10* in the heatmap in Supplemental Figure S2. Secondly, in the original version of Supplemental Figure S2, we plotted the heatmap by using the FPKM values normalized with log2, so the changes caused by ETH or 1-MCP treatment were not obvious. We now plotted the heatmap by using the FPKM values normalized with row scale from zero to one, which showed the expression change trends of every gene better. We also add the FPKM value of Pbr002023.1 in the heatmap in Supplemental Figure S2 to make it clear to see the expression levels.

Line 221-222: A phylogenetic analysis indicated that Pbr002023.1 and Pbr016049.2 might be transcriptional activators (Supp. Fig. 3). One sentence about the tree is not enough. It is really difficult to find out these two factors in the phylogenetic tree. Please highlight it with a different color.

Thank you for your suggestions. We have added descriptions about Pbr002023.1 and Pbr016049.2 in lines 262-274 as follows. "A co-expression network analysis using our previous transcriptome data (Ni et al. 2020) identified four AP2/ERF TF genes, Pbr009056.1, Pbr016224.1, Pbr002023.1 and Pbr016049.2, which had expression patterns that were positively related with *PpMYB114* and *PpMYB10* expression (Supplemental Figure S2). A phylogenetic analysis indicated that Pbr009056.1 and Pbr016224.1 were class II repressors, while Pbr002023.1 and Pbr016049.2 were homologous genes of AT2G41710.1 (AtSMOS1) and AT1G22190.1 (AtRAP2.4), respectively (Supplemental Figure S3). AtSMOS1 and AtRAP2.4 were reported to be transcriptional activators in previous studies (Nomoto et al. 2022; Li et al. 2022). Thus, Pbr002023.1 and Pbr016049.2 might also be transcriptional activators. However, Pbr002023.1 was expressed at very low levels in all samples (Supplemental Figure S2). Thus, Pbr016049.2, which was named *PpRAP2.4* according to the name of the homologous gene in Arabidopsis, was selected for further analyses." Furthermore, we highlighted these two genes by red arrows in Supplemental Figure S3.

Line 225: PpRAP2.4 according to the names of the homologous genes in Arabidopsis. Please include the Atg number in the text.

Thank you for your suggestions. We have added the Atg number in the text in line 268.

Figure 3C: The third panel (BD-RAP2.4) shows that RAP2.4 is an activator. Is it necessary to fuse it with a VP16 activation domain?

Thank you for your comments. In Figure 3C, RAP2.4-BD shows that RAP2.4 is an activator. So, there is no necessary to fuse it with a VP16 activation domain. We have deleted the results of VP16 and RAP2.4-VP16-BD.

Figure 3F: Although RAP2.4 binds the MYB114 promoter (possibly a weak binding), marginal activation (less than two-fold) suggests this is NOT the major regulator.

Thank you for your comments. In our luciferase assay, we found that PpRAP2.4 can activate *PpMYB114* promoter, the relative luciferase activity is about 2 folds. In Y1H and EMSA assays, we also confirmed that PpRAP2.4 can bind to *PpMYB114* promoter through the GCC-box. Furthermore, our results also showed that *PpMYB114* was transcriptionally regulated by PpERF9. Thus, we think that PpMYB114 might be regulated by various regulators. In our study, PpRAP2.4 could be a minor regulator in regulating PpMYB114 compared with PpERF9.

Figure 4D: Can we say that the expression of ANS in RAP2.4-OE fruit is significantly different than control? Is it average of three replicates? Expression of key pathway genes is repressed in RAP2.4-OE fruits. Is it expected?

Thank you for your comments. We have repeated the transient overexpression of *PpRAP2.4* in 'Hongzaosu' pear fruit to analyze the result again. We modified the transient overexpression assay to get a better effect. We cloned *PpARAP2.4* into pCAMBIA1301 vector with a 35S promoter and used the empty vector of pCAMBIA1301 as the negative control instead of pGreenII 0029 62-SK vector. Furthermore, we transformed these plasmids into *Agrobacterium Tumefaciens* EHA105 which owned a better infective effect instead of GV3101. After injection, the pear fruit were placed in dark for 3 day and then treated with strong light for 3 days. Thus, the infection time was prolonged from 2 days to 6 days to get a better infective effect. The results showed that the red coloration and anthocyanin content were all improved in *PpRAP2.4-OX* pear fruit peel compared with the fruit peel injected with empty vector (Figure 4A and B). The expression of *PpRAP2.4* was significantly induced in *PpRAP2.4-OX* pear fruit peel compared with control (Figure 4C). Furthermore, the expression levels of *PpMYB114*, *PpMYB10*, *PpUFGT*, *PpANS*, *PpDFR*, *PpF3H*, *PpCHI* and *PpCHS* were all significantly up-regulated in *PpRAP2.4-OX* pear fruit peel compared with control (Figure 4C). Due to the modification of the transient overexpression assay, the results showed a little bit difference with the previous study. But the phenotype in pear fruit and the overall expression trends of the anthocyanin biosynthesis-related genes were consistent with the previous results. Thus, we think that our transient overexpression is relatively stable and our results are convincing. Furthermore, three replicates were applied in this experiment. The error bars represent the standard deviation of the three biological replicates. Asterisks indicate the significantly different values (*P < 0.05 and ** P < 0.01). Based on the repeated results, we updated the description in results in lines 297-299.

Figure 4H: Surprisingly a small change (approx. 25%) in RAP2.4 expression has significant effects on some regulatory (MYB114) and pathway genes (UFGT and ANS).

Thank you for your comments. Actually, the expression of *PpRAP2.4* in the *PpRAP2.4-TRV* fruit sample was about 35% less than the control in the previous result. To get a more convinced results, we have repeated the transient silencing of *PpRAP2.4* in 'Hongzaosu' pear fruit again. We performed the *PpRAP2.4*-silencing and control injection in the same pear fruit in the previous experiment. In the repeated experiment, we performed the *PpRAP2.4*-silencing and control injection in different pear fruit. The results were overall consistent with the previous version, indicating that our VIGS assay is relatively stable and our results are convincing. The average relative expression level of *PpRAP2.4* was approximately 0.674 compared with control. The expression levels of *PpMYB114*, *PpMYB10*, *PpUFGT*, *PpANS*, *PpDFR*, *PpF3H*, *PpCHI* and *PpPAL* were all down-regulated in *PpRAP2.4*-silencing fruit pear peel. We think that the down- regulation of both *PpMYB114* and *PpMYB10* might further repressed the expression of anthocyanin biosynthetic pathway genes. Based on the repeated results, we updated the description in results in lines 301-303. As we have replied to question 6, we referred to some papers published in The Plant Cell in recent years and found the transient silencing of genes in apple fruit (Li et al., 2017; Ma et al., 2021; Wang et al., 2022) also showed approximately 0.5 to 0.7 down-regulated changes, which is similar to our results.

References:

Li T, Xu Y, Zhang L, Ji Y, Tan D, Yuan H, Wang A (2017) The jasmonate-activated transcription factor MdMYC2

regulates ETHYLENE RESPONSE FACTOR and ethylene biosynthetic genes to promote ethylene biosynthesis during apple fruit ripening. The Plant Cell 29:1316-1334

Ma H, Yang T, Li Y, Zhang J, Wu T, Song T, Yao Y, Tian J (2021) The long noncoding RNA MdLNC499 bridges MdWRKY1 and MdERF109 function to regulate early-stage light-induced anthocyanin accumulation in apple fruit. Plant Cell 33:3309-3330

Wang S, Wang T, Li Q, Xu C, Tian J, Wang Y, Zhang X, Xu X, Han Z, Wu T (2022) Phosphorylation of MdERF17 by MdMPK4 promotes apple fruit peel degreening during light/dark transitions. Plant Cell 34:1980-2000

Light treatment of fruits or callus: Why did the light treatment vary in each experiment (from 2 days to 6 days)?

Thank you for your comments. Light treatment itself could promote the anthocyanin biosynthesis and red coloration of peat fruit. In our previous studies, light treatment for 3 days can slightly promote anthocyanin biosynthesis, while light treatment for 6 days can obviously promote anthocyanin biosynthesis in pear (Supplemental Figure S1a- control). Thus, light treatment for 5-7 days is the most suitable time to obverse the inhibition effects of genes on anthocyanin biosynthesis, while light treatment for 3 days is the most suitable time to obverse the promotion effects of genes on anthocyanin biosynthesis. So, the light treatments vary in each experiment.

Figure 5C: RAP2.4 overexpression induces F3H and CHS expression in callus but represses in fruits (Figure 4D). Why is it different in different tissues?

Thank you for your comments. We have repeated the transient overexpression of *PpRAP2.4* in 'Hongzaosu' pear fruit to analyze the result again. We modified the transient overexpression assay to get a better effect. We cloned *PpARAP2.4* into pCAMBIA1301 vector with a 35S promoter and used the empty vector of pCAMBIA1301 as the negative control instead of pGreenII 0029 62-SK vector. Furthermore, we transformed these plasmids into *Agrobacterium Tumefaciens* EHA105 which owned a better infective effect instead of GV3101. After injection, the pear fruit were placed in dark for 3 days and then treated with strong light for 3 days. Thus, the infection time was prolonged from 2 days to 6 days to get a better infective effect. In addition, we used the same pear fruit to perform the injection work to avoid the individual differences between pears in previous experiment, while we used different pear fruit in the repeated experiment to avoid the potential effect of adjacent injection. The results showed that the expression of *PpRAP2.4* was significantly induced in *PpRAP2.4-OX* pear fruit peel compared with control (Figure 4C). Furthermore, the expression levels of *PpF3H* and *PpCHS* were also significantly up-regulated in *PpRAP2.4-OX* pear fruit peel compared with control (Figure 4C). The repeated experiment showed the similar results in *PpRAP2.4-OX* pear calli and in *PpRAP2.4- OX* pear fruit skin. We think that the modification of our transient overexpressing assay process might lead to the differences of the expression levels of *PpF3H* and *PpCHS* between the previous and the repeated results.

Figure 5F: RAP2.4-OE induces the expression of ETH biosynthetic pathway genes and increases ETH release. Did the authors check the expression of ERF9?

Thank you for your suggestion and comment. We have checked the expression of *PpERF9* in the *PpRAP2.4-OX* pear calli. The expression of *PpERF9* was slightly induced in *PpRAP2.4-OX* pear calli probably due to the promotion of the increasing release of ethylene. This can further clarify that overexpression of *PpRAP2.4* in pear calli only slightly induced anthocyanin biosynthesis. We have added this results in Figure 5F and in lines 319-323 in the manuscript.

Figure 5M: It seems anthocyanin accumulation in MCP-treated callus is a consequence of the increased expression of MYB10 as expression of MYB114 is least affected.

Thank you for your comments. *PpMYB10* is an important and positive regulator of anthocyanin biosynthesis in red pear. Overexpression of *PpRAP2.4* in pear calli indeed increased the expression of *PpMYB10*. According to the editors' and reviewers' suggestion, we analyzed the promoter of *PpMYB10*, and found another ERF-binding site (DRE motif) (Supplemental Figure S7). We performed a dual-luciferase assay to analyze the potential regulatory pattern of PpRAP2.4 on *PpMYB10*. The results showed that PpRAP2.4 had no direct effect on the *PpMYB10* promoter activity (Supplemental Figure S7). So, we think that PpRAP2.4 might affect some other factors which can directly regulate *PpMYB10* expression. We will further explore how *PpMYB10* is regulated by ethylene in the future. We added the results of dual-luciferase assay in lines 285-288.

Figure 5Q: A nominal change (approx. 20-25%) in the expression level of RAP2.4 has a huge impact on anthocyanin accumulation and expression of regulatory and structural genes. This is not often observed in silencing experiments either using a stable or transient system.

Thank you for your comments. We checked our original data and found that the expression level of *PpRAP2.4* was 0.679 in *PpRAP2.4-RNAi* pear calli compared with control pear calli. The significance analysis showed that P value was less than 0.01. So we think our results is credible. The RNAi of genes in apple fruit or calli (Li et al., 2017; Liu et al., 2022; Ma et al., 2021; Wang et al., 2022), strawberry (Zhang et al., 2020) also showed an approximately 0.5 to 0.7 down-regulated changes. The efficiency of genes silencing in fruit might be lower than that in model plants such as tomato or Arabidopsis.

References:

Liu Y, Zhang XW, Liu X, Zheng PF, Su L, Wang GL, Wang XF, Li YY, You CX, An JP (2022) Phytochrome interacting factor MdPIF7 modulates anthocyanin biosynthesis and hypocotyl growth in apple. Plant Physiology 188:2342-2363

Ma H, Yang T, Li Y, Zhang J, Wu T, Song T, Yao Y, Tian J (2021) The long noncoding RNA MdLNC499 bridges MdWRKY1 and MdERF109 function to regulate early-stage light-induced anthocyanin accumulation in apple fruit. Plant Cell 33:3309-3330

Wang S, Wang T, Li Q, Xu C, Tian J, Wang Y, Zhang X, Xu X, Han Z, Wu T (2022) Phosphorylation of MdERF17 by MdMPK4 promotes apple fruit peel degreening during light/dark transitions. Plant Cell 34:1980-2000

Zhang Z, Shi Y, Ma Y, Yang X, Yin X, Zhang Y, Xiao Y, Liu W, Li Y, Li S, Liu X, Grierson D, Allan AC, Jiang G, Chen K (2020) The strawberry transcription factor FaRAV1 positively regulates anthocyanin accumulation by activation of FaMYB10 and anthocyanin pathway genes. Plant Biotechnology Journal 18:2267-2279

Line 282: PpERF9 indirectly inhibits PpMYB114 expression by transcriptionally repressing PpRAP2.4. Both ERF9 and RAP2.4 binds the same cis-elements in MYB114 promoter to regulate its activity (repress or activate). Is it indirect? It seems to me there are two different mechanisms.

Thank you for your comments. In this study, we found that PpERF9 could inhibit *PpMYB114* expression through two pathways. The first pathway is that PpERF9 directly binds to the promoter of *PpMYB114* to inhibit its expression. The second pathway is that PpERF9 binds to the promoter of *PpRAP2.4* to inhibit its expression; therefore, the promotion of PpMYB114 by PpRAP2.4 is blocked. According to the editor's and your suggestion, we deleted the "indirectly" in line 339.

Line 294-295: While expression of RAP2.4 is repressed by 20-25% in ERF9- OE fruits or callus, it doesn't change ERF9 in VIGS fruits (Figure 2). However, the effect of ERF9 is quite significant in the promoter assay (Figure 6). ERF9 binding to the RAP2.4 promoter is not quite clear. Please explain.

Thank you for your comments. We have repeated the transient overexpressing assay and the VIGS assay of *PpERF9* in 'Hongzaosu' pear fruit again to analyze the results. We modified the transient overexpression and VIGS assays to get better effects. For transient overexpression assays, we cloned the CDS of genes into pCAMBIA1301 vector with a 35S promoter and used the empty vector of pCAMBIA1301 as the negative control instead of pGreenII 0029 62-SK vector. Furthermore, we transformed these plasmids into *Agrobacterium Tumefaciens* EHA105 which owned a better infective effect instead of GV3101 (Figure 2A-C). For the VIGS assays, we prolonged the infection time to 6 or 7 days instead of 3 days to get a better effect (Figure 2D-F). To confirm whether the pTRV vector in VIGS assay was expressed in the fruit peel, a pair of PCR primers which are located in the pTRV2 vector were used to run the PCR using fruit peel cDNA (Responding Fig. 3A). The results indicated that the vector of VIGS assay was indeed expressed in the transient silencing fruit peel (Responding Fig. 3B). Furthermore, a small RNA sequencing was performed to confirm that much more small RNA was generated in the target gene fragment in the *PpERF9-TRV* pear fruit peel sample than in control pear fruit peel sample (Responding Fig. 4). In addition, we performed the PpERF9-OX/silencing and control injection in the same pear fruit in the previous experiment, while we performed the PpERF9-OX/silencing and control injection in different pear fruit in the repeated experiment. As a result, the expression levels of *PpRAP2.4* was significantly downregulated in the PpERF9-OX pear fruit peel and significantly up-regulated in *PpERF9*-silencing pear fruit peel (Figure 2C and F), which was consistent with that in the *PpERF9-OX* pear calli (Figure 2I). The repeated results showed that PpERF9 indeed inhibits *PpRAP2.4* expression. In Figure 6, EMSA, ChIP-qPCR and dual-luciferase assays all indicated that PpERF9 can directly bind to the promoter of

PpRAP2.4 **and inhibit its expression. Our repeated transient expression results further confirmed that.**

Line 334-345 and Supplement Figure 6: First, why would mutation of EAR motifs in ERF9 turn it into an activator of anthocyanin pathway? Second, why would overexpression of a mutated ERF9 significantly increase the expression MYB10 and structural genes. Third, if it is true, then this is contrary to their previous data where a nominal increase or decrease in ERF9 expression significantly alters anthocyanin accumulation. Here (Supp. Figure 6C), upregulation of anthocyanin pathway gene has no or minimum impact on pigment accumulation (Supp. Fig. 6B).

Thank you for your comments. Firstly, we performed a dual-luciferase assay to confirm the effect of mutated PpERF9 on *PpMYB114* promoter. The result showed that PpERF9-m3 had no effect on *PpMYB114* promoter (Responding Fig. 6). The result was identical to our qPCR analysis in Figure 6G, of which the expression of *PpMYB114* showed no significant difference in *PpERF9-m3-OX* pear calli and control pear calli. Thus, we speculated that PpERF9-m3 might promote anthocyanin biosynthesis by activating some other anthocyanin-related genes.

Secondly, in previous studies, Li et al. (2020) found that ECAP (EAR motif-Containing Adaptor Protein) is a negative regulator of jasmonate-responsive anthocyanin biosynthesis. To analyze the repressive effect of the EAR motif, the authors also constructed a mutation of ECAP, named ECAP^{mEAR}, of which they mutated the EAR motif in the ECAP protein. ECAP^{mEAR} was then overexpressed in the Arabidopsis. The results showed that overexpression of ECAP^{mEAR} also induced the expression of *ANS* in Arabidopsis compared with the WT Arabidopsis, while the anthocyanin content showed slightly change. So we think mutation of EAR motif in the repressor might cause activation of some genes in its downstream pathway.

Thirdly, as we have known, the up-regulation of anthocyanin structural genes is prior to the anthocyanin accumulation. For example, in our study, the expression of most of the anthocyanin structural genes peaked 3 or 5 days after 1-MCP treatment compared with control, but the anthocyanin content peaked 7 days after 1-MCP treatment (Supplemental Figure S1). We speculated that the overexpression of PpERF9-m3 could lead to the up-regulation of the expression of *MYB10* and structural genes in a slower process than the other genes such as *PpRAP2.4* or silencing of PpERF9. Thus, when we sampled the pear calli of control and *PpERF9-m3-OX* at 5 days of light treatment, the expression levels of *MYB10* and structural genes were at the peak while anthocyanin might accumulate the maximum amount later.

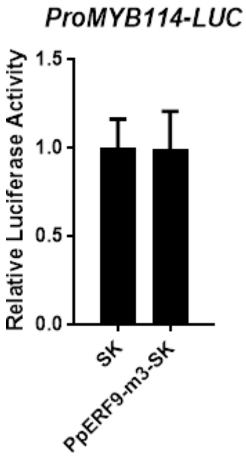

Responding Fig. 6. A dual-luciferase assay demonstrated that PpERF9-m3 had no effect on *PpMYB114* promoter activity.

Reference:

Li C, Shi L, Wang Y, Li W, Chen B, Zhu L, Fu Y (2020) Arabidopsis ECAP Is a new adaptor protein that connects JAZ repressors with the TPR2 co-repressor to suppress jasmonate-responsive anthocyanin accumulation. Mol Plant 13 (2):246-265. doi:10.1016/j.molp.2019.10.014

Figure 8F - TPL RNAi: Similar to the other RNAi results throughout the manuscript, a small change in TPL expression has significant impact on expression of several genes in anthocyanin pathway.

Thank you for your comments. We checked our original data and found that the expression level of *PpTPL1* was 0.594 in *PpTPL1-RNAi* pear calli compared with control pear calli. The significance analysis showed that P value was less than 0.05 (P value = 0.0248). So we think our results is credible. The RNAi of genes in apple fruit or calli (Li et al., 2017; Liu et al., 2022; Ma et al., 2021; Wang et al., 2022), strawberry (Zhang et al., 2020) also showed a down-regulated change with approximately 0.5 to 0.7. The efficiency of genes silencing in fruit might be lower than that in model plants such as tomato or Arabidopsis.

References:

Liu Y, Zhang XW, Liu X, Zheng PF, Su L, Wang GL, Wang XF, Li YY, You CX, An JP (2022) Phytochrome interacting factor MdPIF7 modulates anthocyanin biosynthesis and hypocotyl growth in apple. Plant Physiology 188:2342-2363

Ma H, Yang T, Li Y, Zhang J, Wu T, Song T, Yao Y, Tian J (2021) The long noncoding RNA MdLNC499 bridges MdWRKY1 and MdERF109 function to regulate early-stage light-induced anthocyanin accumulation in apple fruit. Plant Cell 33:3309-3330

Wang S, Wang T, Li Q, Xu C, Tian J, Wang Y, Zhang X, Xu X, Han Z, Wu T (2022) Phosphorylation of MdERF17 by MdMPK4 promotes apple fruit peel degreening during light/dark transitions. Plant Cell 34:1980-2000

Zhang Z, Shi Y, Ma Y, Yang X, Yin X, Zhang Y, Xiao Y, Liu W, Li Y, Li S, Liu X, Grierson D, Allan AC, Jiang G, Chen K (2020) The strawberry transcription factor FaRAV1 positively regulates anthocyanin accumulation by activation of FaMYB10 and anthocyanin pathway genes. Plant Biotechnology Journal 18:2267-2279

Figure 8H: histone acetylation level of RAP2.4 quite significant in TPL-OE lines which is different from ERF9-OE lines. Please explain.

Thank you for your comments. In Figure 6E and Figure 8H, the H3ac level of *PpRAP2.4* locus in *PpERF9-OX* and *PpTPL1-OX* pear calli were similar. But the H3ac level in the S1 fragment just showed slightly decrease in *PpERF9-OX* pear calli, while showed significant decrease in *PpTPL1-OX* pear calli. We checked the previous experiment and original data, we used 3 μL H3ac antibody to perform the ChIP-qPCR assay. Then we repeated the ChIP-qPCR assay twice and modified the process (Responding Fig. 7). We used 4 μL H3ac antibody instead of 3 μL. The results showed that the H3ac level of *PpRAP2.4* locus in *PpERF9-OX* and *PpTPL1-OX* pear calli were consistent. We added the results in Figure 6E and Figure 8H, and changed the description in lines 382-386.

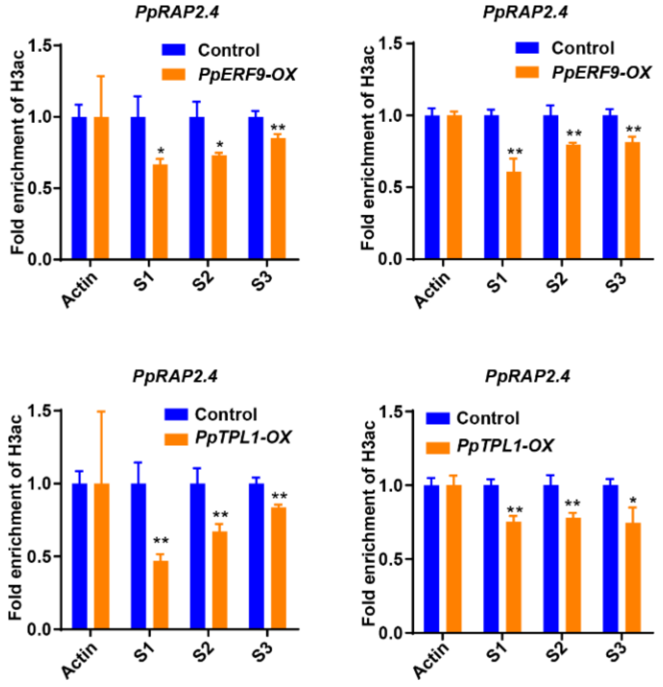

Responding Fig. 7. ChIP-qPCR results reflecting the H3ac levels at *PpRAP2.4* locus in *PpERF9-OX* and *PpTPL1-OX* pear calli.

The first paragraph of this section is merely a repetition of Introduction. Summarize the major findings in few lines.

Thank you for your suggestion and comments. We have summarized the major findings in few lines in the first paragraph of the discussion section in lines 475-490 as following. "Anthocyanins are crucial for plant defenses against various biotic and abiotic stresses. In fruit, anthocyanin play more important roles in signaling, such as serving as visual signals for seed dispersers in ripe fruits (Zoratti et al. 2014). However, most previous anthocyanin-related studies focused on the transcriptional activation of anthocyanin biosynthetic genes; there has been relatively little related research on transcriptional repression. We previously confirmed that ethylene inhibits anthocyanin biosynthesis of pear fruit with different genetic backgrounds (Ni et al. 2020), which is opposite with hat in most fruit species, including apple, plum, and strawberry (Faragher and Brohier 1984; Cheng et al. 2016; Villarreal et al. 2010). But the molecular mechanism underlying the transcriptional repression associated with ethylene-suppressed anthocyanin biosynthesis in pear have remained elusive. Here, we demonstrate that PpERF9, an EAR motif-containing AP2/ERF TF, which was induced by ethylene signaling, acts as a repressor of anthocyanin biosynthesis by forming a repressor complex with PpTPL1 to negatively regulate the transcription of anthocyanin biosynthetic key genes *PpRAP2.4* (a transcriptional activator of *PpMYB114*) and *PpMYB114* in pear."

ERF9 (a repressor) and RAP2.4 (activator) are differentially regulated in response to ETH. Both regulators appear to bind to the same cis-elements in MYB114 promoter to regulate its expression. The significance of this information is not discussed in the manuscript.

Thank you for your suggestion and comments. We have added the discussion of the significance in lines 520-528 as follows: "It is worth noting that PpERF9 and PpRAP2.4 functioned as a repressor and an activator of *PpMYB114* by binding to the same cis- element (GCC-box) in its promoter to regulate its expression. Furthermore, *PpERF9* and *PpRAP2.4* were positively and negatively regulated by ethylene signaling, respectively. When large amount of ethylene is existing, the expression of *PpRAP2.4* is repressed by PpERF9, leading to the banishment of the competitive binding of PpERF9 and PpRAP2.4 in *PpMYB114* promoter. Thus, ethylene signaling can negatively regulate the transcription of *PpMYB114* more efficient through PpERF9-PpMYB114 or PpERF9-PpRAP2.4-PpMYB114 pathways."

Reviewer #2:

In the manuscript entitled "Ethylene-responsive PpERF9 represses PpRAP2.4 and PpMYB114 expression via histone deacetylation to inhibit pear anthocyanin synthesis" by Ni et al. the authors present a well laid out set of results showing an interaction between pear anthocyanin biosynthesis and ethylene repression of this pathway. This is performed by over-expression and knockout of genes in pear callus and pear skin (transiently), and then analysis that utilizes qPCR, CHIP-seq, gel shifts and histone modification state. A model is presented by which this regulatory network functions. The results are relevant to plant biologists interested in the transcriptional regulation of secondary metabolism. The results are generally well presented and well written.

Thank you for your affirmation to our work.

The major issues that need addressing in this paper are contextual. The "other" activator of anthocyanin synthesis (MYB10) in pear is ignored - even though it is even more affected by ethylene and MCP-1 (Supplemental figure 1). Why is this activator ignored? Is it part of the model presented in Fig 9? GCG boxes are found in the promoter of MYB114 - are they absent or present in the promoter of MYB10? Could the effect on MYB10 be via MYB114 activating its expression? Furthermore, MYB10 expression is up when ERF9 is over-expressed (Fig 2I) - why? Wouldn't that encourage anthocyanin accumulation?

Thank you for your comments and suggestions. Firstly, PpMYB10 is an important positive regulator of anthocyanin biosynthesis in pear fruit (Feng et al. 2010). In our study, the expression of *PpMYB10* was significant induced by 1-MCP treatment and inhibited by ethylene treatment as well as *PpMYB114*. Thus, we have changed the description in lines 184-188 to make it more accurate: “Notably, the *PpMYB10* and *PpMYB114* expression levels increased more than 400-fold and 30-fold after the 1-MCP treatment, respectively (relative to the control level), whereas their expression levels were repressed significantly following the ethephon treatment compared with control (Supplemental Figure S1D). These results indicate that ethylene inhibits red pear anthocyanin biosynthesis by regulating *PpMYB10* and *PpMYB114* transcription.”

Secondly, according to the editors’ and reviewers’ suggestion, we further analyzed the promoter of *PpMYB10*, and found another ERF-binding site (DRE motif) (Supplemental Figure S7). We firstly performed the dual-luciferase assay to analyze the potential regulatory pattern of PpRAP2.4 and PpERF9 on *PpMYB10*. The results showed that both PpRAP2.4 and PpERF9 had no effect on the *PpMYB10* promoter activity (Supplemental Figure S7). So, we think that *PpMYB10* was regulated by ethylene in another pathway. We added these results in lines 195-199, 225-227 and 285- 288 of revised manuscript.

Thirdly, PpMYB114 and PpMYB10 are all important regulators of anthocyanin biosynthesis in pear fruit. Among them, PpMYB10 was an ortholog of apple MdMYB10 TF (Feng et al. 2010), while PpMYB114 was identified by linkage to the quantitative trait loci (QTL) for red skin color of Chinese pear (Yao et al., 2017). But the relationship between PpMYB10 and PpMYB114 hasn’t been studied in any previous studies. Based on the reviewer’s suggestion, we performed a dual-luciferase assay to analyze whether PpMYB114 can activate *PpMYB10* promoter. The result showed that PpMYB114 promoted the activity of *PpMYB10* promoter in tobacco leaves (Responding Fig. 1), indicating that PpMYB114 might transcriptionally regulate *PpMYB10* expression. This finding provides us a really good idea for further studies. However, in this study, we mainly focused on the regulation of ethylene-responsive ERF on *MYB* genes. In the future, we will do large amount of experiments including molecular biological verification and genetic verification of the transcriptional regulation of PpMYB114 on *PpMYB10*.

Fourthly, in Figure 2I, the expression of *PpMYB10* was slightly induced in *PpERF9- OX* pear calli. However, there is no significantly difference based on the Student’s *t*- test with the P value 0.051. So we think the insignificantly slight promotion of *PpMYB10* could not promote the accumulation of anthocyanin.

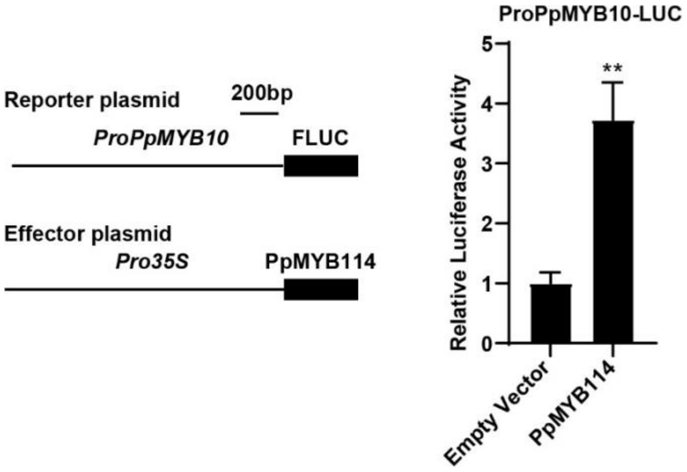

Responding Fig. 1 A dual-luciferase assay demonstrated that PpMYB114 activates *PpMYB10* promoter activity. Error bars represent the standard deviation of six biological replicates. The 'ns' indicates no significance. The promoter of *PpMYB10* was cloned into the pGreenII 0800-LUC (firefly luciferase) vector, and the full length CDS of *PpMYB114* was cloned into the pGreenII 0029 62-SK vector, respectively. The empty vector of SK was used as control. The relative luciferase activity was analyzed.

References:

Feng S, Wang Y, Yang S, Xu Y, Chen X (2010) Anthocyanin biosynthesis in pears is regulated by a R2R3-MYB transcription factor PyMYB10. Planta 232 (1):245-255

Yao G, Ming M, Allan AC, Gu C, Li L, Wu X, Wang R, Chang Y, Qi K, Zhang S, Wu J (2017) Map-based cloning of the pear gene *MYB114* identifies an interaction with other transcription factors to coordinately regulate fruit anthocyanin biosynthesis. The Plant Journal 92 (3):437-451

The other contextual issue is the amount of previous work on the AP2/ERF family. As mentioned in the introduction, and later in the discussion, there are many other members of this family implicated in anthocyanin modulation. This is both via activation and repression. Supplementary figure 3 (phylogeny) should include all these candidates (pear and apple) so the reader can see how similar ERF3 (apple) or pear ERF3 is to ERF9 or PpRAP2.4 etc. ERFs in Ni et al 2021, Zhang et al. 2018b; An et al. 2020; An et al. 2018a, Yao et al. 2017; Ni et al. 2019 etc strawberry work? (e.g. RAV1 in Zhang et al., 2020). Also - in apple MYB10 activates ERF106 (Espley et al., 2019) to elevate ethylene. Line 501 in discussion - apple ERF4 interacts with MdTPL4 - how similar is this? How related are the AP2/ERF family members to published candidates in the anthocyanin/ethylene literature? All these candidates could be shown in the phylogeny and commented on the results/discussion. Just how do the results of this paper fit in or add to these studies can't be ascertained unless we can see how related these genes are.

Thank you for your suggestions. We have added the ERF TFs which positively or negatively regulate anthocyanin biosynthesis or respond to ethylene signaling mentioned in introduction and discussion in the phylogenetic tree (Supplemental Figure S3). We found that AtERF3/4/7/8/9/10/11/12 and MdERF4 were all clustered into class II repressor subgroup together with PpERF9 (Pbr000398.1), while PpERF9 showed the closest relationship with AtERF9 in the phylogenetic tree. Furthermore, MdERF4 showed the closest relationship with Pbr038280.1 in the phylogenetic tree. As we have known, EAR motif-mediated transcriptional repression is associated with co-repressors, including TPL, SAP18, and HDAC. In Arabidopsis, AtERF3, which is an active repressor, physically interacts with AtSAP18, which in turn interacts with AtHDA19 to form a repression complex (Song and Galbraith 2006). AtERF7, which is another EAR motif-containing class II ERF, recruits AtHDA19 by interacting with AtSIN3, thereby decreasing the sensitivity of guard cells to ABA and increasing transpirational water loss (Song et al. 2005). In apple, MdERF4 interacts with MdTPL4 to inhibit fruit ripening and ethylene production, and the transcriptional repression by MdERF4 is modulated by MdTPL4 (Hu et al. 2020). These results further indicated that the interactions between co-repressors and class II ERF repressors are conserved in different plant species. Furthermore, PpRAP2.4 (Pbr016049.2) showed no close relationship with the reported ERF TFs in apple and strawberry, indicated that

RAP2.4 was a novel regulator in controlling anthocyanin biosynthesis. We have added this information in lines 274-277 and 574-577.

References：

Hu Y, Han Z, Sun Y, Wang S, Wang T, Wang Y, Xu K, Zhang X, Xu X, Han Z, Wu T (2020) ERF4 affects fruit firmness through TPL4 by reducing ethylene production. The Plant Journal 103:937-950

Song C-P, Agarwal M, Ohta M, Guo Y, Halfter U, Wang P, Zhu J-K (2005) Role of an Arabidopsis AP2/EREBP-type transcriptional repressor in abscisic acid and drought stress responses. The Plant cell 17:2384-2396

Song C-P, Galbraith DW (2006) AtSAP18, An Orthologue of Human SAP18, is Involved in the Regulation of Salt Stress and Mediates Transcriptional Repression in Arabidopsis. Plant Molecular Biology 60:241-257

Smaller issues - Abstract - line 36 - "...thus forming a PpERF9-PpRAP2.4- PpMYB114 indirect regulatory..." The regulatory module has aspects of direct and indirect. Consider re-wording.

Thank you for your suggestion. According to the editor's and your suggestion. We deleted the "indirect" in the abstract and rewrote as follows (lines 39-42): "Our analyses indicated PpERF9 can directly inhibit *PpMYB114* expression by binding to its promoter. Additionally, PpERF9 inhibits the expression of *PpRAP2.4*, which activates *PpMYB114* expression, by binding to its promoter, thus forming a PpERF9-PpRAP2.4-PpMYB114 regulatory pathway."

In the introduction MYB140 is mentioned - an ethylene induced repressor of MYB114. This adds to the module. ERF105 should be in the phylogeny, and this level of repression (direct protein interaction with MYB114) should be in the model.

Thank you for your suggestion. We have added PpERF105 (Pbr001362.1) which negatively regulate anthocyanin biosynthesis in the phylogenetic tree in Supplemental Figure S3. Furthermore, we also modify the model to add the repressive pathway of PpERF105 in Figure 10. Accordingly, we modified the summary in lines 622-640 based on Figure 10 as follows: "In the presence of ethylene, the expression of PpERF9 is induced. Additionally, PpERF9 interacts with the PpTPL1 co-repressor to form a complex that removes the acetyl group on histone H3 and maintains a low H3ac level in the promoter regions of PpERF9-targeted genes (PpRAP2.4 and PpMYB114) to repress their expression. On the one hand, PpERF9 binds to the PpMYB114 promoter and inhibits its expression via PpERF9-PpTPL1-mediated histone deacetylation, directly inhibiting anthocyanin biosynthesis in pear fruit. On the other hand, PpERF9 represses the expression of PpRAP2.4 via histone deacetylation. PpRAP2.4 induces the expression of PpMYB114 by binding to its promoter. Thus, the PpERF9-PpRAP2.4- PpMYB114 regulatory pathway inhibits anthocyanin biosynthesis in pear fruit. In conjunction with our previous study (Ni et al., 2021), ethylene signaling also induces the expression of PpERF105, which then activates PpMYB140, a transcriptional repressor of anthocyanin biosynthesis by competing with PpMYB114 to form the MBW complex, which ultimately inhibits anthocyanin biosynthesis in pear fruit (Figure 10). Our findings provide new insights into the role of the ethylene signaling pathway in the regulation of anthocyanin biosynthesis. In addition, our data may shed light on the link between the chromatin state and hormone signaling and explain how plants respond to ethylene signals in various pathways."

Discussion "...to accumulate slightly more amount of anthocyanin compared with control..." – reword "accumulates slightly more anthocyanin compared with control..."

Thank you for your suggestion. We changed the sentence in lines 394-396 into "An exposure of *PpERF9-m3*-OX pear calli to strong light with UV-B for 5 days led to the accumulation of slightly more amount of anthocyanin compared with control"

<u>Reviewer #3</u>:

The manuscript describes identification and characterisation of an ethylene induced ERF transcription factor PpERF9 in pear that functions as repressor. PpERF9 binds to promoter region of PpMYB114 anthocyanin regulator or PpRAP2.4, which is activator of PpMYB114 leading to decreased anthocyanin biosynthesis. Also interaction of PpERF with co-repressor PpTPLI via EAR motifs affecting histone deacetylation maintaining low H3ac levels in the promoter regions of PpMYB114 and PpRAP2.4. was presented. The results provide new information on the

mechanism of the inhibition of anthocyanin biosynthesis in pear fruit by ethylene. The work has been performed with adequate methods and the results are plausible. Writing is straightforward and fluent.

We are grateful for your positive comments.

Minor comments:

The layout of figures 5. And 6. made the font so small that they were difficult to read.

Thank you for your suggestion. We have changed the layout of figures 5 and 6 to make the font more suitable to read.

Discussion section begins with the sentence: "Anthocyanins are crucial for plant defences against various biotic and abiotic stresses." I think that in fruit skin anthocyanins have more important roles in signaling, which I would mention here.

Thank you for your comment and suggestion. We really approve your opinion. We added this sentence in the discussion in lines 475-477 as following: "Anthocyanins are crucial for plant defenses against various biotic and abiotic stresses. In fruit, anthocyanin play more important roles in signaling, such as serving as visual signals for seed dispersers in ripe fruits (Zoratti et al. 2014)."

TPC2022-RA-01128 1st Editorial decision – *revision requested* Jan. 9, 2023

We have received reviews of your manuscript entitled "Ethylene-responsive PpERF9 represses PpRAP2.4 and PpMYB114 expression via histone deacetylation to inhibit pear anthocyanin synthesis." On the basis of the advice received, the board of reviewing editors would like to accept your manuscript for publication in The Plant Cell. This acceptance is contingent on revision based on the comments from our reviewers. In particular, please make sure that the figure legends and experimental descriptions are very clear, and that the written part of the results perfectly aligns with what the figures show - this was brought-up a few times by the reviewers.

--- Reviewer comments:

TPC2022-RA-01128R1 1st Revision received Jan. 24, 2023

Reviewer comments on previous submission and **author responses**:

Editors:

Line 99: PAL is not usually considered an anthocyanin biosynthesis enzyme.

Thank you for your suggestion. We have deleted the PAL enzyme.

Reviewer #1:

The authors have addressed my concerns/queries, and the revised manuscript is significantly improved. This manuscript provides novel insights into the molecular mechanism of ET-mediated regulation of anthocyanin biosynthesis in an important fruit crop.

We are grateful to your positive comments. We have carefully revised our manuscript according to the comments kindly from you as follows.

Line 194 ethylene signaling might activate some repressors that...

Thank you for your suggestions. We have revised the sentence in line 194.

Line 209-210 Pbr000398.1 is PpERF9? Good to include ERF9 amino acid sequence with EAR-motif highlighted in the supplement.

Thank you for your comments and suggestions. As you have pointed out, Pbr000398.1 is PpERF9. We added this information in lines 209-210 of revised manuscript as follows: "In addition, there are two EAR motifs in Pbr000398.1

(i.e. PpERF9)…" . In addition, we added the amino acid sequence of PpERF9 in the Supplemental Figure 5B and highlighted the EAR-motif.

Line 575 MaACO1 and expansins to regulate fruit ripening

Thank you for your comment. We have revised "*Expansions*" to "*expansins*" in line 575.

Line 707 Trans-acting activity assay or Transactivation activity assay?

Thank you for your comment. Based on your and the reviewer 4's suggestions. We changed "Trans-acting activity assay" into "trans-activation activity assay" in lines 705- 706.

Reviewer #4:

The manuscript by Ni et al. reported the characterization of PpERF9 as an ethylene-induced transcriptional repressor for anthocyanin biosynthesis in pear. The underlying mechanisms were elucidated by a range of molecular studies along with overexpression and RNA-mediated suppression of candidate genes. PpERF9 suppresses PpMYB114 and PpRAP2.4 expressions through direct promoter binding and the resulting histone deacetylation, with the involvement of PpTPL1 which interacts with the EAR motifs in PpERF9. Compared to transcriptional activation, relatively little is known about transcriptional repression of anthocyanin biosynthesis. This study provides new mechanistic insights into the ethylene-induced inhibition of anthocyanin pigmentation on pear fruit skin. This is particularly interesting as ethylene apparently promotes anthocyanin production in many other fruits. Given the physiological and horticultural importance of anthocyanin pigmentation on pear fruit, the transient expression experiments in pear fruits are particularly relevant.

We are grateful to your positive comments. We have carefully revised our manuscript according to the suggestions and comments kindly from you as follows:

Vector controls showed different pigmentation responses on pear skins. This issue needs to be clarified before interpretation of the results from overexpression and silencing. In Fig 2A, it appears that the pCAMBIA vector were inducing strong anthocyanin biosynthesis around the injection sites, whereas in Fig 2D, the TRV injections only cause very weak pigmentations. Similarly, vector controls showed different responses between Fig. 4A and 4D, but this time the TRV vector, but not the pCAMBIA vector, was inducing strong pigmentations around the injection sites. Meanwhile, the vector control injections showed quite different phenotypes in the same pear cultivar (Hongzaosu) as reported in Fig. 6e of Yao et al. (2017). Did the use of different vectors and/or Agrobacterium strains result in different injection phenotypes on peach skin?

Thank you for your comments. Light treatment itself could promote the anthocyanin biosynthesis and red coloration of peat fruit. In our previous studies, light treatment for 3 days can slightly promote anthocyanin biosynthesis, while light treatment for more than 5 days can obviously promote anthocyanin biosynthesis in pear (Supplemental Figure S1a-control). Thus, we proposed light treatment for 6 days is the most suitable time to observe the inhibition effects of genes on anthocyanin biosynthesis, while light treatment for 3 days is the most suitable time to observe the promotion effects of genes on anthocyanin biosynthesis. Thus, we use 6 d and 3d treatments for anthocyanin repression and promotion genes, separately. So, in Figure 2A and Figure 4D, we preformed the light treatment for 6 days to observe the inhibition effects of PpERF9- OX and PpRAP2.4-TRV. Furthermore, in Figure 2D and Figure 4A, we preformed the light treatment for 3 days to observe the promotion effects of PpERF9-TRV and PpRAP2.4-OX. This is the reason for the different coloration levels of control treatments in different figures, and it is the difference in light duration rather than the different vectors or strains that leads to the different injection phenotypes of pear skin.

Furthermore, in a study by Yao et al. (2017), they used the unbagged fruitlets of 'Hongzaosu' pear to perform the transient transformation. The peel of fruitlets of 'Hongzaosu' pear accumulated high level of anthocyanin and was red. In our experiment, we used bagged mature 'Hongzaosu' pear fruit to perform the transient transformation. Therefore, our fruit samples were colorless before light treatment. Thus, the control injections showed quite different phenotypes in our study and in Yao's study.

What were the rationale behind the use of different dark/light treatments in Fig. 2A vs 2D and Fig. 4A vs 4D?

Thank you for your comments. As mentioned in question 1, light treatment itself could promote the anthocyanin

biosynthesis and red coloration of peat fruit. In our previous studies, light treatment for 3 days can slightly promote anthocyanin biosynthesis, while light treatment for more than 5 days can obviously promote anthocyanin biosynthesis in pear (Supplemental Figure S1a-control). Thus, we proposed light treatment for 6 days is the most suitable time to observe the inhibition effects of genes on anthocyanin biosynthesis, while light treatment for 3 days is the most suitable time to observe the promotion effects of genes on anthocyanin biosynthesis. Thus, we use 6 d and 3d treatments for anthocyanin repression and promotion genes, separately. So, in Figure 2A and Figure 4D, we preformed the light treatment for 6 days to observe the inhibition effects of PpERF9-OX and PpRAP2.4-TRV. Furthermore, in Figure 2D and Figure 4A, we preformed the light treatment for 3 days to observe the promotion effects of PpERF9-TRV and PpRAP2.4-OX.

Which part of the skin is collected for pigment and gene expression analyses? Just the regions surrounding the injection areas? This should be specified in the manuscript

Thank you for your comments and suggestions. The regions surrounding the injection areas were collected for pigment and gene expression analyses. We have added this information in materials and methods in lines 761-762 as follows: "For all the transient transformation assay, the regions surrounding the injection areas were collected for pigment and gene expression analyses."

Lines 463-471: Any data on whether ethylene treatment inhibits histone acetylation of PpPAP2.4 similar to those shown for PpMYB114 in Fig. 9?

Thank you for your comments. We have analyzed the H3ac levels of PpRAP2.4 in the fruit peel treated with ethephon and 1-MCP. The results were added in Figure 9. Furthermore, we added the description of the results in lines 469-481 as follows:

"Finally, we examined the H3ac levels of *PpMYB114* and *PpRAP2.4* in the fruit peel treated with ethephon and 1-MCP by performing a ChIP-qPCR assay involving the anti- H3ac antibody. In *PpMYB114* locus, after a 3-day treatment, the H3ac level decreased in the S2 region of the ethephon-treated pear fruit peel, but increased in the S2 and S3 regions of the 1-MCP-treated pear fruit peel (Figure 9A). Following a 5-day treatment, the H3ac level decreased in the S3 region of the ethephon-treated pear fruit peel, but increased in the S1–S3 regions of the 1-MCP-treated pear fruit peel (Figure 9B). Furthermore, in *PpRAP2.4* locus, the H3ac level showed no significant difference in the analyzed regions of the ethephon-treated pear fruit peel compared with control after 3-day and 5-day treatment, but increased in the S2 region of the 1-MCP-treated pear fruit peel after a 3-day treatment and increased in the S1-S3 regions of the 1-MCP- treated pear fruit peel after a 5-day treatment (Figure 9C and D). These results indicate that ethylene inhibits the expression of *PpMYB114* via histone deacetylation."

The effects of ETH and 1-MCP on ethylene productions should be mentioned early in the Results section before describing their effects on different gene expressions

Thank you for your suggestions. We have added the description in lines 178-180 as follows. "We recently revealed that ethylene inhibits anthocyanin biosynthesis in red pear fruit with different genetic backgrounds (Ni et al. 2020); this finding was further confirmed in this study by treating 'Hongzaosu' pear fruit with ethephon or 1-methylcyclopropene (1-MCP, an ethylene inhibitor). The results showed that ethephon treatment inhibited 'Hongzaosu' red coloration while 1-MCP induced that (Supplemental Figure S1)."

Is anthocyanin accumulation light-dependent under all conditions tested in this study? For example, could 1-MCP treatment or ERF9 suppression induce anthocyanins under the dark?

Thank you for your comments. Anthocyanin accumulation is light-dependent under all the conditions tested in this study. In our previous studies, we found that ethylene inhibits anthocyanin biosynthesis with different genetic background (Ni et al., 2020). And we performed 1-MCP treatment in the dark and found that only 1-MCP without light could not induce anthocyanin biosynthesis in pear fruit (Responding Figure 1). Furthermore, in the process of experiment, we found that PpERF9 suppression and PpRAP2.4 overexpression also could not induce anthocyanin biosynthesis under dark condition.

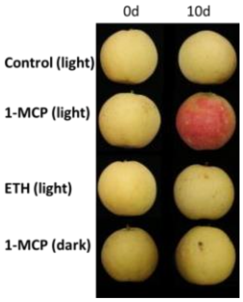

Responding Figure 1. Phenotypes of pear fruit following ethephon and 1-MCP treatments with light treatment and dark.

Reference: Ni J, Zhao Y, Tao R, Yin L, Gao L, Strid A, Qian M, Li J, Li Y, Shen J, Teng Y, Bai S (2020) Ethylene mediates the branching of the jasmonate-induced flavonoid biosynthesis pathway by suppressing anthocyanin biosynthesis in red Chinese pear fruits. Plant Biotechnology Journal 18:1223-1240

Lines 216-217: "A transcriptional activity assay confirmed that PpERF9 is a repressive TF", but in Fig. 1B, C, the figure legends describe that PpERF9 activities transcription.

Thank you for your comments. We are sorry for the mistakes in the figure legends. We have revised the figure legends accordingly.

MYB10 expression is upregulated in Fig 2I but is down-regulated in 2C where PpER9 is over-expressed in both cases. How come? Do the levels of MYB10 expression affect anthocyanin production? Do MYB114 and MYB10 share the same target genes involved in anthocyanin biosynthesis?

Thank you for your comments. Actually, in Figure 2I, the expression of *PpMYB10* showed no significant difference in *PpERF9-OX* pear calli compared with control based on the Student's *t*-test with the P value 0.051 (see Supplemental Data Set S2). We think that the difference of materials (pear fruit and pear calli) might lead to the delicate difference of the expression patterns.

PpMYB10 is also an important transcription factor which positively regulate anthocyanin biosynthesis in pear fruit (Feng et al., 2010). Previous studies showed that PpMYB10 and PpMYB114 both positively regulate the expression of the key anthocyanin biosynthetic gene *PpDFR*, *PpANS* and *PpUFGT* (Bai et al., 2017; Yao et al., 2017). Thus, PpMYB10 and PpMYB114 can all promote the accumulation of anthocyanin in pear fruit peel.

Reference:

Bai S, Sun Y, Qian M, Yang F, Ni J, Tao R, Li L, Shu Q, Zhang D, Teng Y (2017) Transcriptome analysis of bagging-treated red Chinese sand pear peels reveals light-responsive pathway functions in anthocyanin accumulation. Scientific Reports 7:63

Feng S, Wang Y, Yang S, Xu Y, Chen X (2010) Anthocyanin biosynthesis in pears is regulated by a R2R3-MYB transcription factor PyMYB10. Planta 232:245-255

Yao G, Ming M, Allan AC, Gu C, Li L, Wu X, Wang R, Chang Y, Qi K, Zhang S, Wu J (2017) Map-based cloning of the pear gene MYB114 identifies an interaction with other transcription factors to coordinately regulate fruit anthocyanin biosynthesis. The Plant Journal 92:437-451

Lines 340-342: "These observations suggest that PpRAP2.4 rapidly induces anthocyanin biosynthesis by activating PpMYB114 expression when ethylene production is inhibited": Besides Fig. 5, results from Fig. 3 should be mentioned (i.e. RAP2.4 binds to the GCC-box of MYB114 promoter and transactivates their promoter activities in LUC assays) to support this claim.

Thank you for your suggestion. We have revised this sentence in lines 338-341 to mention the results from Figure 3, 4 and 5 as follows. "These observations suggest that PpRAP2.4 rapidly induces anthocyanin biosynthesis by activating *PpMYB114* expression *via* binding to the GCC-box of its promoter when ethylene production is inhibited

(Figures 3-5).”

Figure 10: In the diagram (not legends), it is suggested to indicate that DRE is the binding site for ERF9 in pPpRAP2.4 and that GCC-box is the binding site for ERF9 and RAP2.4 in pPpMYB114 with opposite effects

Thank you for your suggestions and comments. We have modified the proposed model in Figure 10 to indicate the DRE motif is the binding site for ERF9 in pPpRAP2.4 and that GCC-box is the binding site for ERF9 and RAP2.4 in pPpMYB114 with opposite effects.

Lines 402 and 450: Strong (white) light with UV-B: conditions for the UV-B irradiation were not mentioned in Methods

Thank you for your comment. The UV-B light intensity we used is 1.1 μmol·m^{-2}·s^{-1}. We added the information in materials and methods in lines 753-754, 760-761.

Lines 707, 708: "Trans-activation" instead of "trans-acting"

Thank you for your comment. We changed “Trans-acting activity assay” into “trans- activation activity assay” in lines 705-706.

Suppl. F1: What is BT?

Thank you for your comment. BT means “before treatment” in Supplemental Figure S1. We have added this information.

TPC2022-RA-01128R1 2nd Editorial decision – *accept with minor revisions* Feb. 6, 2023

We have reviewed your resubmission of the manuscript entitled "Ethylene-responsive PpERF9 represses PpRAP2.4 and PpMYB114 expression via histone deacetylation to inhibit pear anthocyanin synthesis." based on the satisfactory response to all the concerns raised by the reviewers, the board of reviewing editors would like to accept your manuscript for publication in The Plant Cell. This acceptance is contingent on minor revisions based on the minor comments below:

Abstract

- I suggest you change "thus forming a PpERF9-PpRAP2.4-PpMYB114 regulatory pathway" by "thus forming a PpERF9-PpRAP2.4-PpMYB114 regulatory circuit". Also in lane 82.
- Your results don't show that reduced histone acetylation is responsible for MYB114 and RAP2.4 expression. Thus, I recommend you change the wording to say the following: "Collectively, these results indicate that ethylene inhibits anthocyanin biosynthesis by a mechanism that involves PpERF9-PpTPL1 complex-mediated histone deacetylation of PpMYB114 and PpRAP2.4."
- Lane 51: Change "would" by "will"
- Lane 85: change "which lead to" to "are associated with"

Results

- Lane 178: Change "revealed" by "showed"
- Lanes 191 - 194: Formally the results don't demonstrate inhibition of transcription but reduction in mRNA levels - you may want to soften the statement to keep this possibility in mind
- Lane 215: replace "implying" by "suggesting"
- Legend figure 3: remove "hot"

-- Reviewer comments:

TPC2022-RA-01128R2 2nd Revision received Feb. 8, 2023

Reviewer comments on previous submission and **author responses**:

Editors:

I suggest to change "thus forming a PpERF9-PpRAP2.4-PpMYB114 regulatory pathway" by "thus forming a PpERF9-PpRAP2.4-PpMYB114 regulatory circuit". Also in lane 82.

Thank you for your suggestion. We have changed "pathway" by "circuit" in lines 42 and 82.

Your results don't show that reduced histone acetylation is responsible for MYB114 and RAP2.4 expression. Thus, I recommend to change the wording to say the following: "Collectively, these results indicate that ethylene inhibits anthocyanin biosynthesis by a mechanism that involves PpERF9-PpTPL1 complex-mediated histone deacetylation of PpMYB114 and PpRAP2.4."

Thank you for your suggestion. We have revised the corresponding sentences according to your suggestion.

Lane 51: Change "would" by "will"

Thank you for your suggestion. We have changed "would" by "will" in line 51.

Lane 85: change "which lead to" to "are associated with"

Thank you for your suggestion. We have changed "which lead to" by "which are associated with" in line 85.

Lane 178: Change "revealed" by "showed"

Thank you for your suggestion. We have changed "revealed" by "showed" in line 178.

Lanes 191 - 194: Formally the results don't demonstrate inhibition of transcription but reduction in mRNA levels - you may want to soften the statement to keep this possibility in mind

Thank you for your suggestion. We have changed "transcription" into "mRNA levels" in line 191-194.

Lane 215: replace "implying" by "suggesting"

Thank you for your suggestion. We have changed "implying" to "suggesting" in line 215.

Legend figure 3: remove "hot"

Thank you for your suggestion. We have removed "hot" in the figure legend of Figure 3.

TPC2022-RA-01128R2 3rd Editorial decision – *acceptance pending* **Feb. 13, 2023**

We are pleased to inform you that your paper entitled "Ethylene-responsive PpERF9 represses PpRAP2.4 and PpMYB114 expression via histone deacetylation to inhibit pear anthocyanin synthesis" has been accepted for publication in The Plant Cell, pending a final editorial review by a science editor.

Final acceptance from Science Editor **Mar. 2, 2023**
